# The charge-assisted hydrogen-bonded organic framework (CAHOF) self-assembled from the conjugated acid of tetrakis(4-aminophenyl)methane and 2,6-naphthalenedisulfonate as a new class of recyclable Brønsted acid catalysts

**DOI:** 10.3762/bjoc.16.99

**Published:** 2020-05-26

**Authors:** Svetlana A Kuznetsova, Alexander S Gak, Yulia V Nelyubina, Vladimir A Larionov, Han Li, Michael North, Vladimir P Zhereb, Alexander F Smol'yakov, Artem O Dmitrienko, Michael G Medvedev, Igor S Gerasimov, Ashot S Saghyan, Yuri N Belokon

**Affiliations:** 1 Nesmeyanov Institute of Organoelement Compounds, Russian Academy of Sciences, Vavilov Street 28, 119991 Moscow, Russian Federation; 2 Moscow State University, Faculty of Material Science, Leninskie Gory 1/73, 119991 Moscow, Russian Federation; 3 Department of Inorganic Chemistry, People’s Friendship University of Russia (RUDN University), Miklukho-Maklaya Street 6, 117198 Moscow, Russian Federation; 4 Green Chemistry Centre of Excellence, Department of Chemistry, University of York, Heslington, YO10 5DD, United Kingdom; 5 Siberian Federal University, School of Non-Ferrous Metals and Material Science, 95 Krasnoyarskiy Rabochiy pr., 660025 Krasnoyarsk, Russian Federation; 6 N. D. Zelinsky Institute of Organic Chemistry RAS, Leninsky Prospect, 47, 119991 Moscow, Russian Federation; 7 Institute of Pharmacy, Yerevan State University, 1 Alex Manoogian Str, Yerevan 0025, Armenia

**Keywords:** Brønsted acid catalyst, charge-assisted hydrogen-bonded framework, Diels–Alder, epoxide ring opening, heterogeneous catalyst

## Abstract

The acid–base neutralization reaction of commercially available disodium 2,6-naphthalenedisulfonate (NDS, 2 equivalents) and the tetrahydrochloride salt of tetrakis(4-aminophenyl)methane (TAPM, 1 equivalent) in water gave a novel three-dimensional charge-assisted hydrogen-bonded framework (CAHOF, **F-1**). The framework **F-1** was characterized by X-ray diffraction, TGA, elemental analysis, and ^1^H NMR spectroscopy. The framework was supported by hydrogen bonds between the sulfonate anions and the ammonium cations of NDS and protonated TAPM moieties, respectively. The CAHOF material functioned as a new type of catalytically active Brønsted acid in a series of reactions, including the ring opening of epoxides by water and alcohols. A Diels–Alder reaction between cyclopentadiene and methyl vinyl ketone was also catalyzed by **F-1** in heptane. Depending on the polarity of the solvent mixture, the CAHOF **F-1** could function as a purely heterogeneous catalyst or partly dissociate, providing some dissolved **F-1** as the real catalyst. In all cases, the catalyst could easily be recovered and recycled.

## Introduction

Tremendous successes in homogeneous catalysis are well-known and documented [1–3]. However, problems associated with catalyst recovery limit the application of homogeneous catalysts in industry and sometimes make their heterogenization necessary. Unfortunately, the immobilization of a homogeneous catalyst onto supports, such as polystyrene, silica, glass, and others [4–12] generally leads to a deterioration of the catalytic properties of the initial homogeneous catalyst. This is due to factors including the nonhomogeneous structures of the catalytic centers on the surface of the carrier or inside the polymeric matrix and the low availability of the active sites to the substrates due to diffusion problems. Additionally, the self-association of catalytic centers on flexible polymeric chains may negatively influence the expected activity of the immobilized catalyst. Moreover, the degradation of cross-linked covalent polymeric matrixes or the destruction of catalytic centers during productive cycles can shorten the lives of the catalysts to an extent that makes the immobilization of homogeneous catalysts impractical [7].

In recent decades, novel classes of heterogeneous, porous, crystalline architectures have been discovered, which allow a rigid and uniform distribution of a single well-defined catalytic or precatalytic center within a solid matrix. Of these, metal–organic frameworks (MOFs) [13–18] and covalent organic frameworks (COFs) [19–22] have been the forerunners. The design of MOFs is based on metal nodes linked by organic ligands whilst COFs have ligands joined by organic nodes. Both displayed great catalytic properties, sometimes exceeding those of homogeneous analogs [23–24]. Unfortunately, stability problems, the cost of the initial materials, and the synthetic protocols for the matrix synthesis hamper the routine use of MOFs and COFs in industry, even for the production of high-added-value products.

Recently, new supramolecular porous materials named hydrogen-bonded organic frameworks (HOFs) or supramolecular organic frameworks (SOFs) have been developed [25–36]. Usually, a HOF is built from multitopic tectons that interact with their neighbors by directional hydrogen bonds, disfavoring close packing, and thus generating significant pore volumes within the crystal [25–28]. These heterogeneous, crystalline, supramolecular frameworks may be neutral, for example, those built by mutual interactions of multitopic carboxylic acids [25–29]. Alternatively, they can be constructed from components possessing oppositely charged multitopic tectons, in which case the framework becomes a charge-assisted hydrogen-bonded framework (CAHOF), as was the case when multitopic guanidinium or amidinium cations were combined with polycarboxylates, polysulfonates, or polyphosphonates [30–33]. The synthesis of a HOF or CAHOF consists of simply mixing the two components together [29]. An additional advantage of HOFs and CAHOFs is their self-healing property, as the frameworks can easily self-reassemble after the disassembly induced by an external stimulus [26–27,37].

Although the field of HOF and CAHOF applications is still in its infancy, there are some promising advances in proton conductivity [30–31], gas separation and absorption [28–29], enzyme encapsulation [36], and even asymmetric synthesis (albeit with a framework that contained a transition metal ion) [37]. However, for the HOF and CAHOF catalysts to have a similar appeal to other regular active site distribution materials, such as zeolites, MOFs, or COFs, a broader scope of applications has to be investigated. We thought that the CAHOFs present a very promising material, as they can be considered as heterogeneous ionic liquids with a great potential for becoming efficient heterogeneous, purely organic catalysts. In particular, salts that are insoluble in organic solvents and derived from the neutralization of polyacidic and polybasic tectones could be good candidates for becoming efficient heterogeneous Brønsted acids.

Herein, we report the synthesis of a novel, purely organic, charge-assisted hydrogen-bonded self-assembled organic framework **F-1**. The structure of **F-1** was established by single crystal and powder X-ray diffraction, NMR spectroscopy, and elemental analysis. The morphology of **F-1** was assessed by SEM, and its stability was determined by TGA. We report the use of **F-1** as a heterogeneous, robust, and recoverable catalyst for the Brønsted acid-catalyzed ring opening reactions of epoxides with alcohols and water, with the latter reaction occurring in a three-phase medium. In addition, a Diels–Alder reaction was promoted by **F-1** in heptane.

## Results and Discussion

**Scheme 1:**
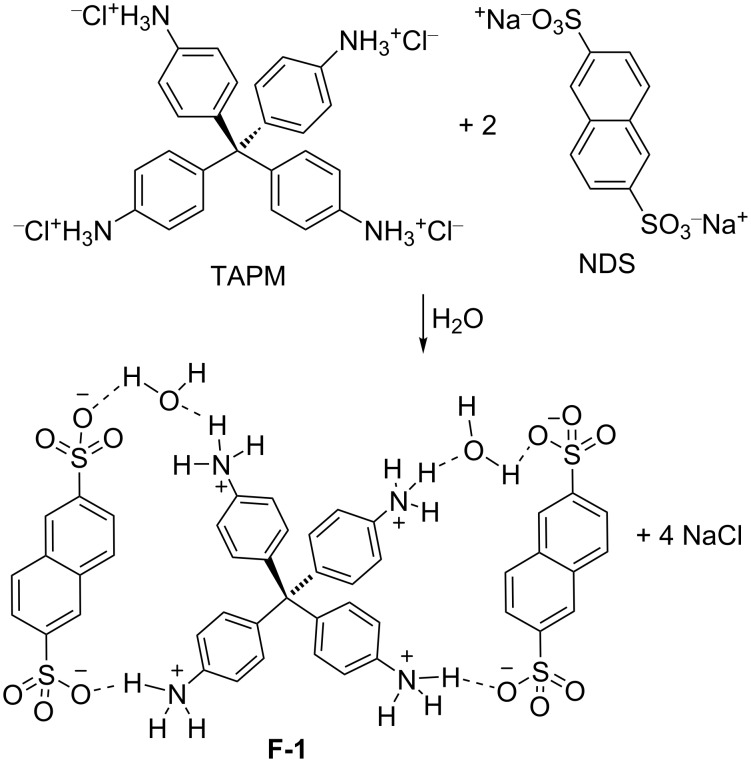
The synthesis of **F-1**.

Mixing together aqueous solutions of two equivalents of NDS and of one equivalent of TAPM at ambient temperature immediately produced **F-1** as a white precipitate (Scheme 1). The p*K*_a_ (in water) of NDS is expected to be −11 to −10, by analogy with the p*K*_a_ of polystyrene sulfonic acids [12]. The four p*K*_a_s of the conjugated acids of TAPM were calculated (see Supporting Information File 1) to be 4.94, 4.46, 4.04, and 3.79. Thus, the difference in the acidity of the NDS and TAPM components was large enough to ensure a complete salt formation.

**Figure 1:**
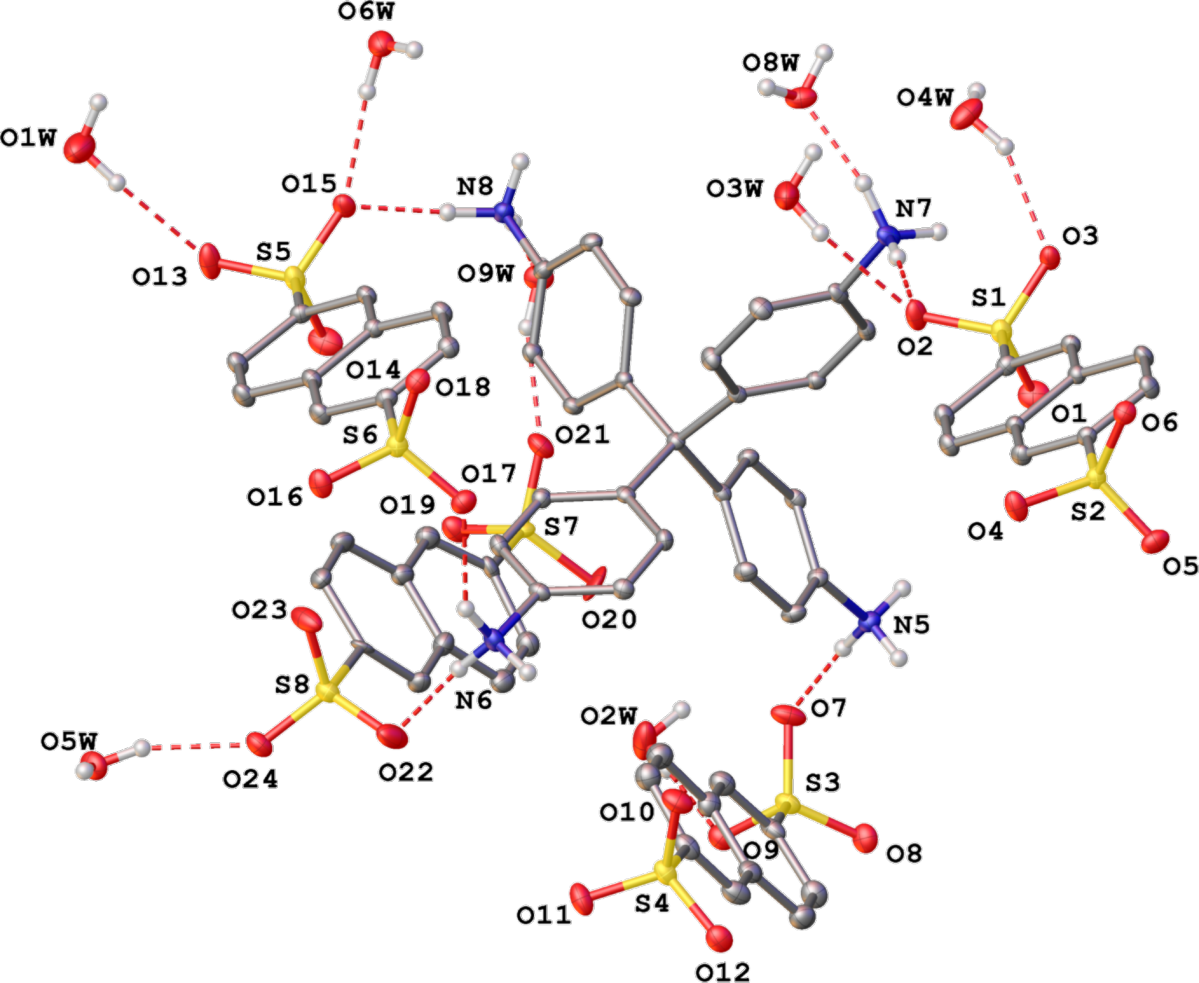
View of the crystal structure of **F-1** (**F-1a** phase), with representation of atoms by thermal ellipsoids at a 30% probability level. The hydrogen atoms, except for those in NH groups and solvate water molecules, were omitted for clarity. Only the labels of symmetry-independent heteroatoms are shown.

Solid **F-1** was practically insoluble in organic solvents with the exception of DMSO. The analytical data supported its structure as depicted in Scheme 1. A crystal of the compound was grown by diffusion of water into a solution of **F-1** in DMSO. The results of the X-ray diffraction analysis are shown in Figure 1. The single crystal material of **F-1** (**F-1a** phase) was in the monoclinic space group *P*2_1_/*c*, with the lattice parameters *a* = 20.6034(8) Å, *b* = 20.1330(8) Å, *c* = 22.4357(8) Å, β = 91.989(1)°, and cell volume = 9300.9(6) Å^3^ at 120 K. An asymmetric part of the unit cell contained two ammonium cations, four sulfonate anions, and nine water molecules, held together by numerous hydrogen bonds (Table S2, Supporting Information File 1), so that the resulting three-dimensional network had no macro- or mesopores (Figure S1, Supporting Information File 1). The volume of the unit cell that was potentially accessible to a solvent was only 29.0 Å^3^, as calculated by PLATON [38].

The same crystal phase (**F-1a**) was present in **F-1** before the crystallization, as confirmed by powder diffraction data collected at room temperature for the white precipitate obtained from the mixed water solutions of TAPM and NDS. Space group *P*2_1_/*c*, *a* = 20.9609(10) Å, *b* = 19.7563(9) Å, *c* = 22.6642(10) Å, β = 92.694(3)°, cell volume = 9375.1(8) Å^3^.

When **F-1** was submitted to vacuum drying at 100 °C for several hours, a sample **F-1b** was obtained. X-ray powder diffraction showed that **F-1b** contained a mixture of unknown phases (Figure S3, Supporting Information File 1). However, after a few hours of being exposed to air, it reverted into the phase **F-1a**, the same phase it had before the drying. Space group *P*2_1_/*c*, *a* = 20.8677(13) Å, *b* = 20.0951(13) Å, *c* = 22.6324(15) Å, β = 92.432(5)°, cell volume = 9482.1(11)Å^3^ (Figures S4 and S5, Supporting Information File 1). The different powder patterns obtained for the initially formed **F-1** and for **F-1b** immediately after vacuum drying suggested that some structural parameters, such as the water content, varied in the two analyses.

**Figure 2:**
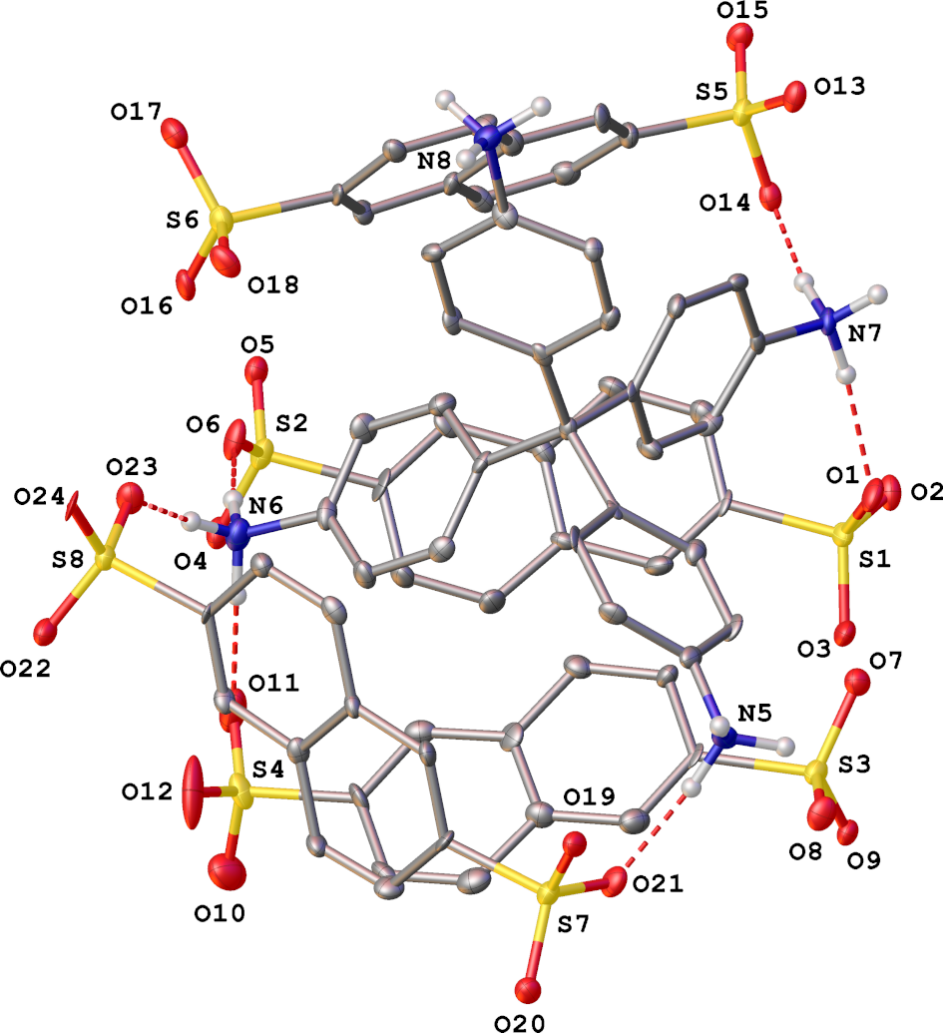
View of the crystal structure of **F-1** (**F-1a’** phase), with representation of the atoms via thermal ellipsoids at a 30% probability level. The hydrogen atoms, except for those in NH groups, were omitted for clarity. Only the labels of symmetry-independent heteroatoms are shown.

The final proof that the phase change was due to some water molecules escaping the crystal, and this proof came from the X-ray diffraction analysis of heated crystals of **F-1** (with **F-1a** phase) that were immediately put into silicon grease and cooled to 120 K at the diffractometer. The data collection revealed a triclinic *P*-1 phase, with the lattice parameters *a* = 13.416(7) Å, *b* = 13.887(7) Å, *c* = 22.730(12) Å, α = 88.564(8)°, β = 87.351(8)°, γ = 89.836(9)°, cell volume = 4229(4) Å^3^. The resulting structure designated as **F-1** (**F-1a’** phase, Figure 2) had two ammonium cations and four sulfonate dianions in the asymmetric part of the unit cell, with no traces of water molecules. Its three-dimensional network is built by charge-assisted hydrogen bonds between the ions (Table S3, Supporting Information File 1), with small voids occurring near the sulfonate and ammonium groups (Figure S2, Supporting Information File 1). The solvent-accessible volume of the unit cell was 105.8 Å^3^, as calculated by PLATON [38]. The dried sample with the **F-1a’** phase readily absorbed water, reverting to the **F-1a** phase with nine molecules of water for every two residues of TAPM within 10–20 minutes of atmospheric exposure.

In addition, **F-1** reversibly took up methanol (1.5 molecules per TAPM moiety), benzene (4–5 molecules per TAPM), and propylene oxide (1.5 molecules per TAPM) in a closed vessel saturated with the vapors of these compounds. The absorbed material changed its PRXD reversibly, returning to its original structure after the absorbed solvent was allowed to evaporate from the sample.

**Figure 3:**
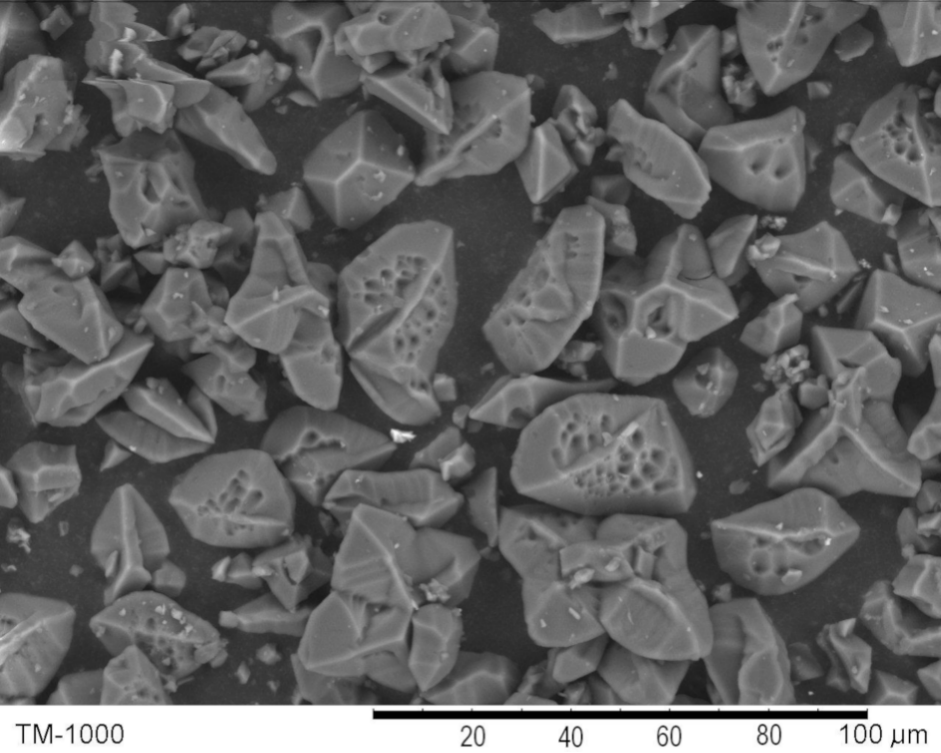
SEM image of **F-1**.

The morphology of uncrystallized **F-1** was studied by scanning electron microscopy (SEM) analysis. It had a “tangerine wedge” morphology (Figure 3 and Figures S9 and S10, Supporting Information File 1), with evident macropores present on the surface of the particles. The size distribution of the **F-1** particles was in the range of 3–5 to 45–50 μm, and most of the particles had a size within a range of 15–30 μm.

**Figure 4:**
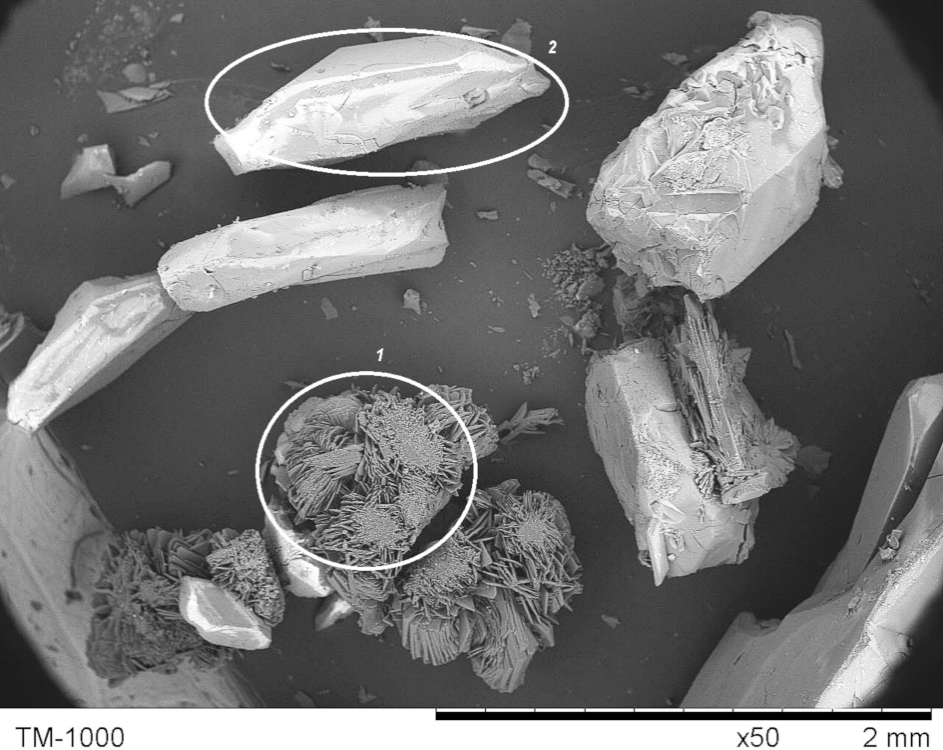
SEM image of **F-1** with an **F-1a** phase.

SEM imaging of crystals of **F-1** in the **F-1a** phase formed from a DMSO/water system indicated the existence of two types of crystals (Figure 4). Type 1 was a set of platelets with heights of 0.7–1 mm, grown from a common planar base with a diameter of 0.1–0.3 mm. In other words, the crystals were a typical druse setup. Type 2 were well-formed, nonisotropic crystals with two parallel planes in varying sizes (0.3 × 0.4 × 1 mm).

**Figure 5:**
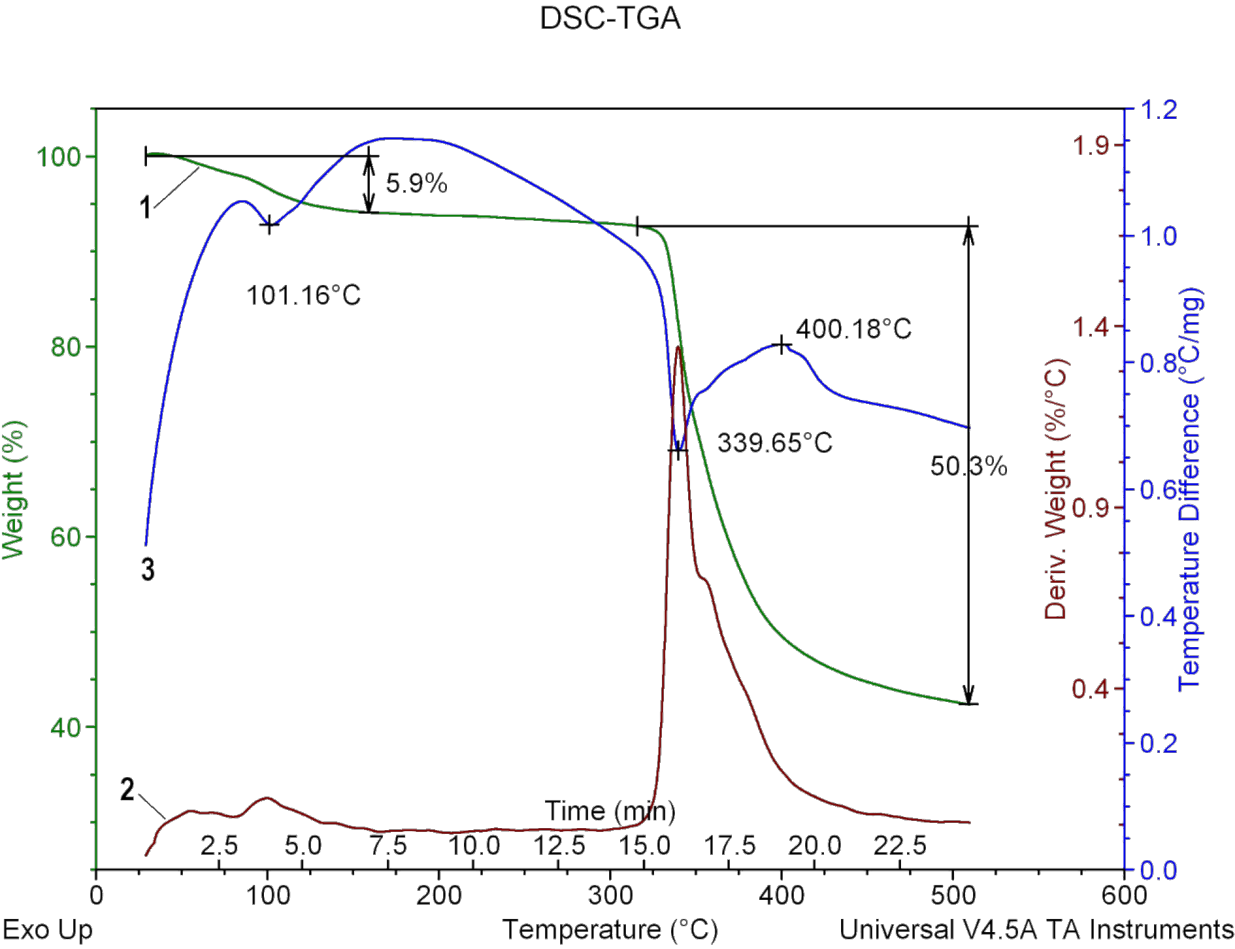
TGA-DSC analysis of a sample of **F-1**. The TGA plot is shown in green, the DSC curve is shown in blue, and the first differential of the DSC curve is shown in red.

The thermogravimetric analysis and differential scanning calorimetry (TGA-DSC) of **F-1** was conducted to examine its thermal properties. The TGA curve of the bulk crystals (Figure 5) reached a plateau at 160 °C after 5.9% of the mass was removed as water. The plateau was maintained until 340 °C, when the sample underwent an endothermic decomposition. The decomposition produced sulfur dioxide, naphthalene, and aniline, according to the infrared spectra of the produced gases (Figures S11 and S12, Supporting Information File 1).

**Scheme 2:**
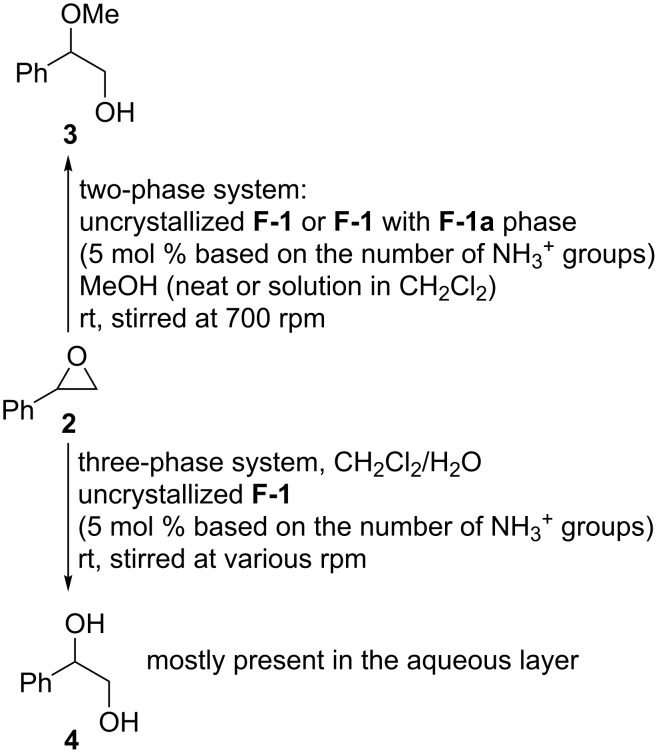
Uncrystallized **F-1** or **F-1** with an **F-1a** phase promoted the two- and three-phase reactions of styrene oxide (**2**).

The CAHOF **F-1** was also analyzed by nitrogen porosimetry (see Supporting Information File 1). It was found to contain mesopores and macropores with an adsorption average pore width of 5.2 nm, a BET surface area of 2.606 m^2^⋅g^−1^, a mesopore volume of 0.00093 cm^3^⋅g^−1^, a macropore volume of 0.00168 cm^3^⋅g^−1^, and a total pore volume of 0.00336 cm^3^⋅g^−1^. The porosimetry data could not be directly relevant to the catalytic activity of the composite as the framework as the porosimetry sample needed to be thoroughly degassed prior to the analysis. However, the framework **F-1** underwent “breathing” in organic solvent solutions (see below), which allowed catalytic sites to become available without the need for a pore structure in the desolvated material. The calculated acidity of the components of **F-1** (see above) indicated possible catalytic applications of the material. Thus, the catalytic properties of uncrystallized **F-1** and **F-1** with an **F-1a** phase were explored in a series of reactions typically promoted by Brønsted acids, such as epoxide ring openings with methanol and water (Scheme 2). The reactions were conducted at room temperature, and after several hours, the catalyst was removed by centrifugation or filtration through a dense paper filter. The filtrates were evaporated, and the residue was weighed and analyzed by ^1^H NMR spectroscopy. The experimental results are summarized in Table 1.

**Table 1:** The ring opening of styrene oxide (**2**) by MeOH or H_2_O, promoted by uncrystallized **F-1** or **F-1** with an **F-1a** phase at room temperature.

run	catalyst	nucleophile	*t* (h)	conversion (%)	yield (%)

1	none	MeOH (neat)	24	0	0
2^a^	**F-1**	MeOH (neat)	1	100	>98
3^b^	**F-1**	MeOH (neat)	1	100	>98
4^a,c^	**F-1** filtrate	MeOH (neat)	1	67	67
5^d^	**F-1**	MeOH in CH_2_Cl_2_	24	56	53–56
6^c,d^	**F-1** filtrate	MeOH in CH_2_Cl_2_	24	<1	<1
7^d^	**F-1a**	MeOH in CH_2_Cl_2_	24	24	22
8^e,f^	**F-1**	H_2_O/CH_2_Cl_2_	3	3	3
9^e,g^	**F-1**	H_2_O/CH_2_Cl_2_	3	3	3
10^e,h^	**F-1**	H_2_O/CH_2_Cl_2_	3	40	40
11^e,i^	**F-1**	H_2_O/CH_2_Cl_2_	3	52	52
12^e,j^	**F-1**	H_2_O/CH_2_Cl_2_	3	55	55
13^e,h,k^	**F-1** filtrate	H_2_O/CH_2_Cl_2_	3	45	45
14^e,h^	**F-1**	H_2_O/CH_2_Cl_2_	24	100	95
15^l^	IR-120	H_2_O/CH_2_Cl_2_	3	0	0

^a^Reaction conditions: **2** (0.2 mL, 1.83 mmol), MeOH (10 mL), uncrystallized **F-1** or **F-1** with an **F-1a** phase (0.023 g, 9.61 × 10^−5^ mol of ^+^NH_3_ groups, 5.3 mol %), stirred at 700 rpm (unless indicated otherwise). ^b^**F-1** was recovered, and reused five times in pure MeOH. Each of these reactions gave a full conversion after 1 hour, and the yield given is that from the 5th use of the catalyst. ^c^**2** (0.2 mL, 1.83 mmol), MeOH (1.5 mL, 36.6 mmol), CH_2_Cl_2_ (10 mL), uncrystallized **F-1** (or **F-1** with an **F-1a** phase, 0.023 g, 9.61 × 10^−5^ mol of ^+^NH_3_ groups). ^d^The same reaction conditions as in the runs 2 or 5, but the catalyst was filtered before the start of the reaction, and the filtrate was used as the catalyst. ^e^**2** (1 mL, 9.17 mmol), CH_2_Cl_2_ (25 mL), and H_2_O (50 mL), uncrystallized **F-1** (0.11 g, 0.46 mmol of ^+^NH_3_ groups). ^f^The reaction was not stirred. ^g^The reaction was stirred at 200 rpm. ^h^The reaction was stirred at 700 rpm. ^i^The reaction was stirred at 1000 rpm. ^j^The reaction was stirred at 1400 rpm. ^k^The same reaction conditions as in run 10, but the catalyst **F-1** was filtered 15 minutes after the reaction had started, and the filtrate was used as the catalyst. ^l^The same conditions as in run 5, but instead of **F-1**, IR-120 in an H^+^ form (0.15 g, 0.485 mmol) mixed with PhNH_2_ (0.485 mmol) was used as a catalyst.

Uncrystallized **F-1** promoted the ring opening of styrene oxide (**2**) with methanol. Within 1 hour at room temperature, the alcohol **3** was obtained in a quantitative yield and as a single regioisomer (Table 1, run 2). The CAHOF **F-1** was robust and retained the catalytic activity after being recovered from the reaction mixture five times (Table 1, run 3). In addition, its ^1^H NMR spectrum was unchanged after being used in five catalytic cycles (Figure S17, Supporting Information File 1). In the absence of a catalyst, no reaction occurred under the experimental conditions (Table 1, run 1). To determine if the reaction was being catalyzed by a homogeneous or heterogeneous species, the CAHOF **F-1** and methanol were mixed together and stirred for 15 minutes. Then, the remaining solid **F-1** was removed by filtration, and the filtrate was tested as a catalyst for the ring opening reaction. The alcohol **3** was obtained in 67% yield (Table 1, run 4), proving that some soluble components of **F-1** were catalytically active, and hence that the reaction was partly catalyzed heterogeneously and partly promoted by the leached catalyst under the experimental conditions. To investigate this in more detail, studies on the solubility of **F-1** in MeOH were conducted by UV–vis spectroscopy at 275 nm (ε = 5670), and it was found that **F-1** had a solubility of 0.25 g/L in MeOH. Hence, under the reaction conditions, 10% of **F-1** would be dissolved in the reaction mixture. Table 1, run 4 shows that the activity of the dissolved part of **F-1** was sufficient to bring the reaction to 67% completion but not to obtain the full conversion seen in Table 1, run 2 and run 3 where both the dissolved and undissolved parts of **F-1** coexisted. This indicated that both the dissolved and the heterogeneous parts of the catalyst were catalytically active. Once these reactions were complete, the filtrate was evaporated, and **F-1** was recovered from the residue by sedimentation by addition of dichloromethane. The structure of the recovered **F-1** was the same as that of the undissolved **F-1**, illustrating the self-healing properties of the framework.

The catalyst could be made completely heterogeneous by performing the same CAHOF **F-1**-catalyzed reaction in a less polar medium. For this purpose, the reaction was conducted in a mixture of methanol and dichloromethane (1.5/10 by volume), and the yield of the alcohol **3** was 53–56% after 24 hours (Table 1, run 5). The filtrate derived from the stirred **F-1** in this solvent mixture was catalytically inactive, and after 24 hours, the reaction contained the epoxide **2** and traces of **3** (less than 1% yield, Table 1, run 6). This observation clearly showed that dissolved (leached) parts of **F-1**, even if present, could not be responsible for the catalytic performance. To investigate if any leaching did occur, a sample of the filtrate was evaporated and then dissolved in DMSO-*d*_6_. No resonances corresponding to **F-1** were present in the ^1^H NMR spectrum of this sample, which supported the absence of any leaching of the catalyst into the reaction medium. The morphology of uncrystallized **F-1** and **F-1** with an **F-1a** phase (Figure 3 and Figure 4) had an influence on the performance of the catalysts. A ground sample of **F-1** with an **F-1a** phase was less active than uncrystallized **F-1** (Table 1, runs 5 and 7).

It is notable that the framework **F-1** (uncrystallized or with an **F-1a** phase), although possessing few or almost no pores, was still catalytically active. An explanation for this involves the potential capacity of the frameworks to react to external stimuli by increasing the distances between the crystal components by, for example, “breathing” in polar solvents. This may disrupt the nondirectional forces in the crystal whilst leaving the directional hydrogen bonds still present so that the framework remained heterogeneous. Notably, simple organic cages that exhibit guest-induced “breathing” and selective gas separation have been reported [29,39–41]. The reversible rearrangement of the crystal framework of a CAHOF derived from the salt of terephthalic acid and tetrakis(4-amidiniumphenyl)methane, in response to the addition of water or the application of heat, also suggested that this “breathing” was feasible [32]. Closely similar behavior was also detected in “flexible” MOFs, which contracted and expanded their pores in the presence of guest gases [42]. In the limiting case, the framework may even become partially dissolved in a polar solvent.

The CAHOF **F-1** was also catalytically active for the conversion of styrene oxide (**2**) into the diol **4** (Table 1, runs 8–14). This reaction was a three-phase system, including two immiscible liquid phases (dichloromethane and water) and solid **F-1**. As the CAHOF **F-1** was insoluble in dichloromethane and poorly soluble in water, the solid catalyst resided between the dichloromethane and water phases. The epoxide **2** was added to this mixture, and the reaction was stirred. After 3 (or 24) hours, the solid catalyst was filtered, the layers were separated, evaporated, and analyzed. The aqueous layer contained only the diol **4** and some dissolved **F-1**. The organic mixture contained a mixture of the epoxide **2** and the diol **4**. Therein, the catalyst was a homogeneous, water-soluble part of **F-1**. The filtered solution was catalytically active to the same extent as the initial heterogeneous one (Table 1, run 13). Thus, in this case, solid **F-1** served mostly as a reservoir for the production of the soluble catalyst, although the dissolved part could easily be recovered by evaporating the aqueous layer, adding dichloromethane to the residue and filtering the insoluble catalyst.

The efficiency of the multiphase reactions should depend on the rate of stirring the reaction. The runs 8–12 in Table 1 illustrated the dependence of the yield of the diol **4** on the stirring velocity over a three-hour reaction period. Expectedly, without any stirring, the hydrolysis did not proceed (Table 1, run 8), and the same poor performance occurred at a stirring rate of 200 rpm (Table 1, run 9). When the stirring rate was increased to 700 rpm, the yield of diol **4** increased to 40% (Table 1, run 10). There was little dependence of the product yield (40–55%) on the stirring rate above the threshold of 700 rpm (Table 1, runs 10–12). A complete conversion of the epoxide **2** into the diol **4** was observed after a reaction time of 24 hours at a stirring rate of 700 rpm (Table 1, entry 14).

Both the pH of the medium (specific acid catalysis) and general acid catalysis by the ammonium groups of **F-1** were potentially important for the ring opening reactions. The ring openings were conducted in three different media: neat methanol, a mixture of methanol and dichloromethane, and a mixture of water and dichloromethane. The methanol and dichloromethane mixture did not need to be considered as **F-1** was not soluble in this mixture. For the other two solvent mixtures, the four ammonium groups of the protonated form of TAPM had p*K*_a_ values in water (according to the calculations discussed above) ranging from 5.0 to 3.8. The solubility of **F-1** in water was determined by UV–vis spectroscopy to be 0.9 g/L. This corresponded to approximately 50% of the catalyst **F-1** being dissolved in the water phase of the reactions reported in Table 1, runs 12–14. The concentration of the ammonium groups was then 4.0 × 10^−2^ M in water. Assuming that the average p*K*_a_ value of the ammonium groups of the protonated form of TAPM was around 4.0, the pH value of the solution would be between 4 and 5. It is therefore most likely that **F-1** operated via the general acid catalysis mechanism in this solvent mixture. As the p*K*_a_ value of anilinium cations will only change to a small extent when the solvent is changed from water to methanol, the same general acid catalysis mechanism would be expected to occur in reactions carried out in methanol.

For comparison, the commercially available (and most often used heterogeneous Brønsted acid catalyst) cation exchange resin IR-120, which contains sulfonic acid functionalities, was mixed in its hydrogen form with aniline to give a model of **F-1**. An attempted use of the resulting compound with the same amount of ammonium groups as in **F-1** for the conversion of the epoxide **2** into the alcohol **3** was unsuccessful (Table 1, run 15). Evidently, the catalytic properties of the CAHOF **F-1** were superior to those of standard ion exchange materials under these reaction conditions.

**Scheme 3:**
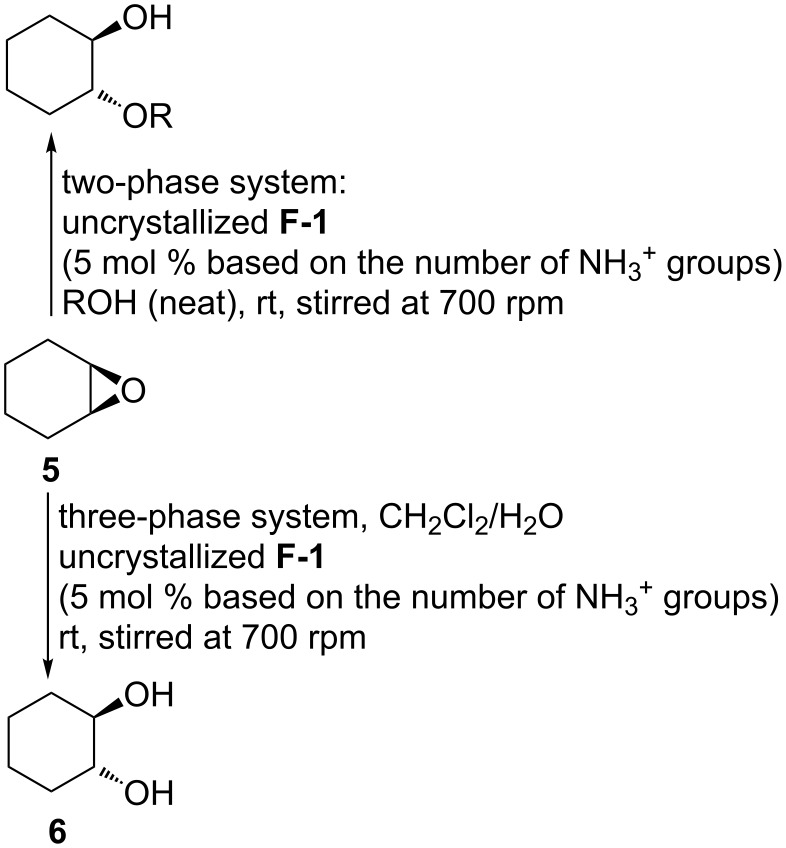
CAHOF **F-1**-promoted reactions of cyclohexene oxide (**5**) with alcohols and water.

The CAHOF **F-1** could also promote the ring opening of cyclohexene oxide (**5**) by alcohols (Scheme 3), with the efficiency of the reaction dropping as the size of the alcohol was increased and its polarity decreased (Table 2, runs 1–3). Water could also be used as the nucleophile (Table 2, runs 4 and 5) and led to the *trans*-cyclohexane diol **6** under the same experimental conditions used for the styrene oxide ring opening. Other epoxides were also studied as substrates for the three-phase ring-opening with water (Table 2, runs 6–9). Propylene oxide and butylene oxide were good substrates for the reaction (Table 2, runs 6 and 7), but hex-1-ene oxide was almost unreactive (Table 2, runs 8 and 9), indicating that some partitioning of the epoxide into the aqueous phase was necessary for reaction to occur. Cyclohexene oxide (**5**) is, however, a good substrate under the same reaction conditions (Table 2, entry 5), possibly due to the greater reactivity of its fused bicyclic ring system.

**Table 2:** Ring opening of epoxides by water or alcohols promoted by **F-1** at room temperature.^a^

run	epoxide	nucleophile	*t* (h)	yield (%) (by ^1^H NMR)

1	**5**	MeOH	4	98
2	**5**	EtOH	4	20
3	**5**	iPrOH	4	<1
4	**5**	H_2_O/CH_2_Cl_2_	3	20
5	**5**	H_2_O/CH_2_Cl_2_	24	80
6	propylene oxide	H_2_O/CH_2_Cl_2_	24	82
7^b^	butylene oxide	H_2_O/CH_2_Cl_2_	24	60
8^b^	hex-1-ene oxide	H_2_O/CH_2_Cl_2_	24	2
9^b^	hex-1-ene oxide	H_2_O/CH_2_Cl_2_	144	10

^a^The epoxide (1.83 × 10^−3^ mol) in 10 mL of alcohol or in a mixture of 5 mL CH_2_Cl_2_ and 10 mL H_2_O was stirred at 700 rpm with **F-1** (0.023 g, 9.61 × 10^−5^ mol of ^+^NH_3_ groups, 5.3 mol %). ^b^The yields were determined by ^1^H NMR spectroscopy, directly from the reaction mixture, by running the experiments in D_2_O.

**Scheme 4:**
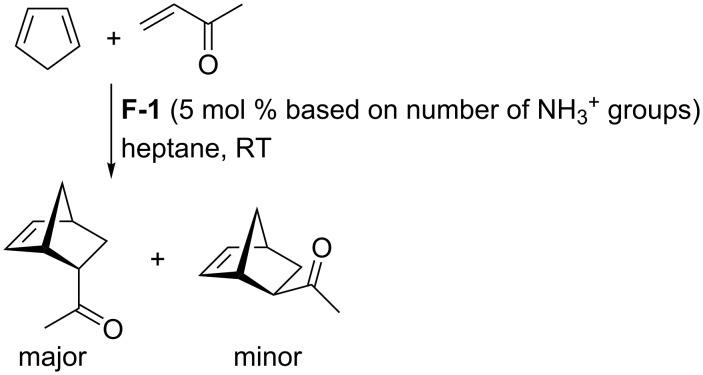
**F-1**-promoted Diels–Alder reaction.

The Diels–Alder reaction of methyl vinyl ketone with cyclopentadiene was also efficiently promoted by the CAHOF **F-1** in heptane at room temperature (Scheme 4). After three hours, the catalyst was filtered, and the filtrate was evaporated to give a mixture of the *endo* and *exo* adducts in a 3.5:1 ratio and a yield of 52%. A reaction carried out under the same conditions in the absence of **F-1** produced the Diels–Alder adduct in a yield of just 10%.

## Conclusion

In summary, by utilizing the acid–base neutralization reaction between two equivalents of NDS and one equivalent of the tetrahydrochloride salt of TAPM in water, a novel three-dimensional material **F-1** was prepared and characterized by X-ray diffraction, TGA-DSC, elemental analysis, and ^1^H NMR spectroscopy. One important role played by NDS was that the crystalline three-dimensional CAHOF **F-1** was supported by hydrogen bonds between the sulfonate anions and the ammonium cations of NDS and TAPM, respectively. By virtue of the three oxygen atoms of each sulfonate of NDS, through which the negative charge was distributed, NDS could support different crystalline arrangements, as shown in Figure 1 and Figure 2.

The framework **F-1** was able to reversibly absorb solvents and water in a process called “breathing”. The material served as a new type of Brønsted acid catalyst in a series of reactions, including epoxide ring opening reactions and a Diels–Alder reaction. A second role for NDS was that one of its sulfonate oxygen atoms could form hydrogen bonds with water whilst leaving the other two oxygen atoms to engage the ammonium groups of TAPM (see the crystal structure of the **F-1a** phase). This structure was thermodynamically stable and hinted at a possible activation of water or methanol as nucleophiles by the sulfate anions during the ring opening of epoxides. When the coordinated water was removed by drying at higher temperatures, another phase, **F-1a’**, was formed (Figure 2). A greater amount of vacant space appeared in the crystal, and the structure became thermodynamically unstable. It reverted to the original **F-1a** phase over a few hours when water was present in the surrounding atmosphere.

Depending on the polarity of the solvent mixture, **F-1** could function as a purely heterogeneous catalyst or as a reservoir, providing some soluble **F-1** as the real catalyst. In all cases the catalyst could easily be recovered and recycled. The system has the potential for future elaboration, for example, by incorporating multitopic tectons with a greater number of negatively charged sulfonate groups mutually rigidly fixed in space. Such arrangement should produce large pores within the framework and further reduce the framework’s solubility in water and organic solvents. Additionally, the acidity of these frameworks could be tuned by varying the ratio of anion/cation multitopic components and the basicity of the cation component.

## Supporting Information

File 1 Experimental, characterization, and p*K*_a_ calculation details as well as SEM and TGA analyses.

File 2 X-ray details for **F-1** (**F-1a** phase).

File 3 X-ray details for **F-1** (**F-1a’** phase).
