# Supplementary material for: The charge-assisted hydrogen-bonded organic framework (CAHOF) self-assembled from the conjugated acid of tetrakis(4-aminophenyl)methane and 2,6-naphthalenedisulfonate as a new class of recyclable Brønsted acid catalysts
Source: Beilstein J Org Chem. 2020 May 26;16:1124–34. doi: 10.3762/bjoc.16.99 (PMC7277948; doi:10.3762/bjoc.16.99)
Supplement: File 1 — Experimental, characterization, and pKa calculation details as well as SEM and TGA analyses. [file Beilstein_J_Org_Chem-16-1124-s001.pdf]

BEILSTEIN JOURNAL OF ORGANIC CHEMISTRY

Supporting Information

for

The charge-assisted hydrogen-bonded organic framework (CAHOF) self-assembled from the conjugated acid of tetrakis(4-aminophenyl)methane and 2,6-naphthalenedisulfonate as a new class of recyclable Brønsted acid catalysts

Svetlana A. Kuznetsova, Alexander S. Gak, Yulia V. Nelyubina, Vladimir A. Larionov, Han Li, Michael North, Vladimir P. Zhereb, Alexander F. Smol'yakov, Artem O. Dmitrienko, Michael G. Medvedev, Igor S. Gerasimov, Ashot S. Saghyan and Yuri N. Belokon

Beilstein J. Org. Chem. **2020,** *16,* 1124–1134. doi:10.3762/bjoc.16.99

Experimental, characterization, and pK_a calculation details as well as SEM and TGA analyses

License and Terms: This is a supporting information file under the terms of the Creative Commons Attribution License (http://creativecommons.org/licenses/by/4.0). Please note that the reuse, redistribution and reproduction in particular requires that the authors and source are credited.
The license is subject to the *Beilstein Journal of Organic Chemistry* terms and conditions: (https://www.beilstein-journals.org/bjoc)

Supporting Information

1. General Information

Solvents were distilled under an argon atmosphere from calcium hydride (CH_2Cl_2) and magnesium turnings/iodine (MeOH). All chemicals were purchased at Sigma-Aldrich and used without further purification. If not stated otherwise, column chromatography was performed with silica gel 60 M from Macherey-Nagel.

2. Instrumentation and methods

Proton nuclear magnetic resonance (^{1}H NMR) spectra and carbon nuclear magnetic resonance (^{13}C NMR) spectra were recorded on a Bruker Avance 400 NMR spectrometer (operating at 400 and 101 MHz, respectively, for ^{1}H and ^{13}C nucleii). Chemical shifts are reported in ppm relative to the residual solvent peak ($CDCl_3$: δ = 7.26 ppm for ^{1}H NMR, δ = 77.2 for ^{13}C NMR; DMSO-d_6: δ = 2.50 ppm for ^{1}H NMR, δ = 39.5 for ^{13}C NMR; D_2O: δ = 4.79 ppm for ^{1}H NMR). NMR data are reported as follows: chemical shift, multiplicity (br = broad, s = singlet, d = doublet, t = triplet, q = quartet, m = multiplet), coupling constant, integration, and nucleus.

High-resolution mass spectra (HRMS) were recorded on a Bruker micrOTOF II instrument using electrospray ionization (ESI). The measurements were done in a positive ion mode (interface capillary voltage – 4500 V) or in a negative ion mode (3200 V); mass range from *m*/*z* 50 to *m*/*z* 3000; external or internal calibration was done with ESI Tuning Mix, Agilent. A syringe injection was used for solutions in acetonitrile, methanol, or water (flow rate 3 mL/min). Nitrogen was applied as a dry gas; interface temperature was set at 180 °C.

Nitrogen-physisorption adsorption measurements were carried out at 77 K using a Micromeritics ASAP 2010 volumetric adsorption analyser. Before measuring, the powdered samples were dried under vacuum for 6 h at 100 °C.

Elemental analyses were carried out in the Laboratory of Microanalysis of A.N. Nesmeyanov Institute of Organoelement Compounds of the Russian Academy of Sciences.

X-ray crystallography. Single crystals of **F-1a** suitable for the X-ray diffraction study were obtained by water diffusion into a DMSO solution of **F-1**. The crystals of **F-1a’** were derived by drying **F-1a** at 100 °C over P_2O_5 in vacuo. X-ray diffraction experiments were carried out at 120 K with a Bruker APEX2 DUO CCD diffractometer, using graphite monochromated Mo Kα radiation (λ = 0.71073 Å). Using the Olex2 structure solution program,[S1] the structures were

solved by Intrinsic Phasing and refined with the olex2.refine[S2] refinement package using Least-Squares minimization against F^2 in anisotropic approximation for non-hydrogen atoms. Hydrogen atoms of NH groups and solvent water molecules were located from difference Fourier synthesis; positions of other hydrogen atoms were calculated. All hydrogen atoms were then refined in isotropic approximation within the riding model. Crystal data and structure refinement parameters for **F-1a** and **F-1a'** are given in Table **S1**.

Table S1. Crystal data and structure refinement parameters for **F-1a** and **F-1a'**.

	**F-1a**	**F-1a'**
Empirical formula	$C_{40}H_{48}N_4O_{15}S_3$	$C_{90}H_{98}N_8O_{33}S_8$
Formula weight	921.00	2076.24
Crystal system	Triclinic	Monoclinic
Space group	P-1	$P2_1/c$
Z	2	4
a, Å	12.0359(14)	20.6034(8)
b, Å	13.3668(16)	20.1330(8)
c, Å	15.0583(18)	22.4357(8)
A	72.938(2)	90
β, °	76.234(2)	91.9890(10)
Γ	69.757(2)	90
V, $Å^3$	4401.2(5)	9300.9(6)
D_{calc} (g cm^{-1})	1.424	1.483
Linear absorption, μ (cm^{-1})	2.47	2.83
F(000)	968	4344
$2\theta_{max}$, °	55	52
Reflections measured	23485	91375
Independent reflections	9855	18284
Observed reflections [I > 2σ(I)]	6266	13065
Parameters	620	1416
R1	0.0627	0.0578
wR2	0.1856	0.1719
GOF	1.017	1.028
$\Delta\rho_{max}$/ $\Delta\rho_{min}$ (e $Å^{-3}$)	1.243/-0.687	1.367/-0.482

Table S2. Hydrogen bonds in **F-1a**.

**D, donor**	**H, hydrogen**	**A, aceptor**	**d(D-A)/Å**	**D-H-A/°**
N1	H1A	O5W[1]	2.789(4)	164(3)
N1	H1C	O3[2]	2.748(4)	176(5)
N2	H2A	O7W[1]	2.711(4)	168(4)
N2	H2B	O13[3]	2.766(4)	167(4)
N2	H2C	O24[1]	2.849(4)	179(4)
N3	H3A	O8[4]	2.768(4)	172(4)
N3	H3B	O11	2.792(4)	168(4)
N3	H3C	O6	2.757(4)	166(4)
N4	H4A	O2W	2.738(4)	160(4)
N4	H4C	O5[2]	2.782(4)	120(4)
N4	H4C	O4W[2]	2.803(4)	152(4)
N5	H5A	O7[4]	2.785(4)	176(3)
N5	H5B	O12	2.754(4)	164(3)
N5	H5C	O18[4]	2.730(4)	168(3)
N6	H6A	O17[5]	2.745(4)	144(4)
N6	H6B	O6W[4]	2.770(4)	166(4)
N6	H6D	O22[4]	2.779(4)	171(4)
N7	H7A	O2[6]	2.774(4)	170(4)
N7	H7B	O8W[5]	2.723(4)	165(4)
N7	H7D	O21[7]	2.84(2)	173(4)
N8	H8A	O15[5]	2.739(4)	171(3)
N8	H8B	O9W	2.748(4)	160(3)
O2W	H2WB	O9	2.780(4)	177.5
O4W	H4WA	O3[8]	2.809(4)	152.7
O4W	H4WB	O20'[4]	2.81(3)	162.4
O5W	H5WB	O24[1]	2.776(4)	162.0
O6W	H6WB	O15	2.837(3)	174.7
O7W	H7WB	O11	2.773(3)	133.1
O8W	H8WB	O7[4]	2.812(3)	142.9
O9W	H9WA	O1[8]	2.856(3)	166.7
O9W	H9WB	O21'[4]	2.804(12)	159.8
O9W	H9WB	O21[4]	2.771(13)	152.1

The atoms are generated from the basic ones by the symmetry operations [1]3-X,1-Y,-Z; [2]3-X,-1/2+Y,-1/2-Z; [3]1+X,+Y,+Z; [4]+X,3/2-Y,1/2+Z; [5]2-X,2-Y,-Z; [6]-1+X,3/2-Y,1/2+Z; [7]2-X,1/2+Y,-1/2-Z; [8]3-X,1/2+Y,-1/2-Z.

Table S3. Hydrogen bonds in **F-1a'**.

**D, donor**	**H, hydrogen**	**A, aceptor**	**d(D-A)/Å**	**D-H-A/°**
N1	H1A	O18[1]	2.841(13)	161.7
N2	H2A	O16	2.745(13)	151.1
N2	H2B	O22[2]	2.756(12)	164.3
N2	H2C	O12[2]	2.831(13)	157.7
N3	H3B	O9	2.811(12)	161.6
N3	H3C	O3	2.799(13)	169.2
N4	H4B	O4	2.826(12)	173.2
N4	H4C	O5[3]	2.774(11)	166.8
N5	H5A	O8[4]	2.798(12)	138.6
N5	H5B	O21	2.787(12)	172.0
N6	H6A	O6	2.801(13)	171.8
N6	H6B	O23	2.721(14)	158.8
N7	H7A	O1[5]	2.775(11)	162.5
N7	H7B	O14[6]	2.814(12)	171.3
N7	H7C	O2	2.720(12)	172.2
N8	H8C	O20[6]	2.886(12)	159.1

The atoms are generated from the basic ones by the symmetry operations [1]-1+X,+Y,+Z; [2]1-X,1-Y,1-Z; [3]-X,2-Y,1-Z; [4]1-X,1-Y,-Z; [5]-X,2-Y,-Z; [6]+X,1+Y,+Z.

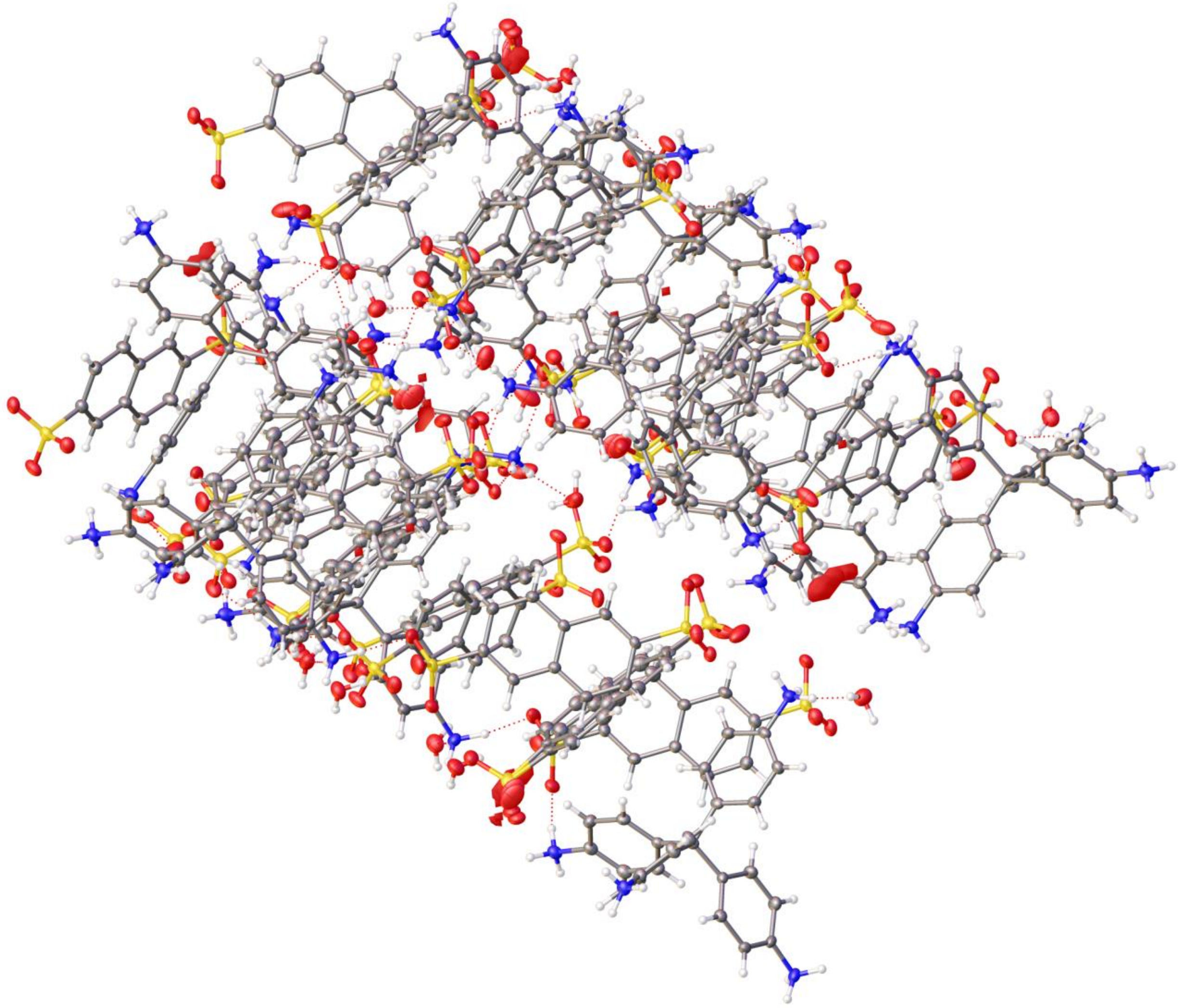

Figure S1. A fragment of the crystal packing in **F-1a** showing solvent-accessible voids by red isosurfaces, as calculated by *calcvoid* routine implemented in Olex2[S1]; the volume of the largest spherical void is 7.24 Å^3.

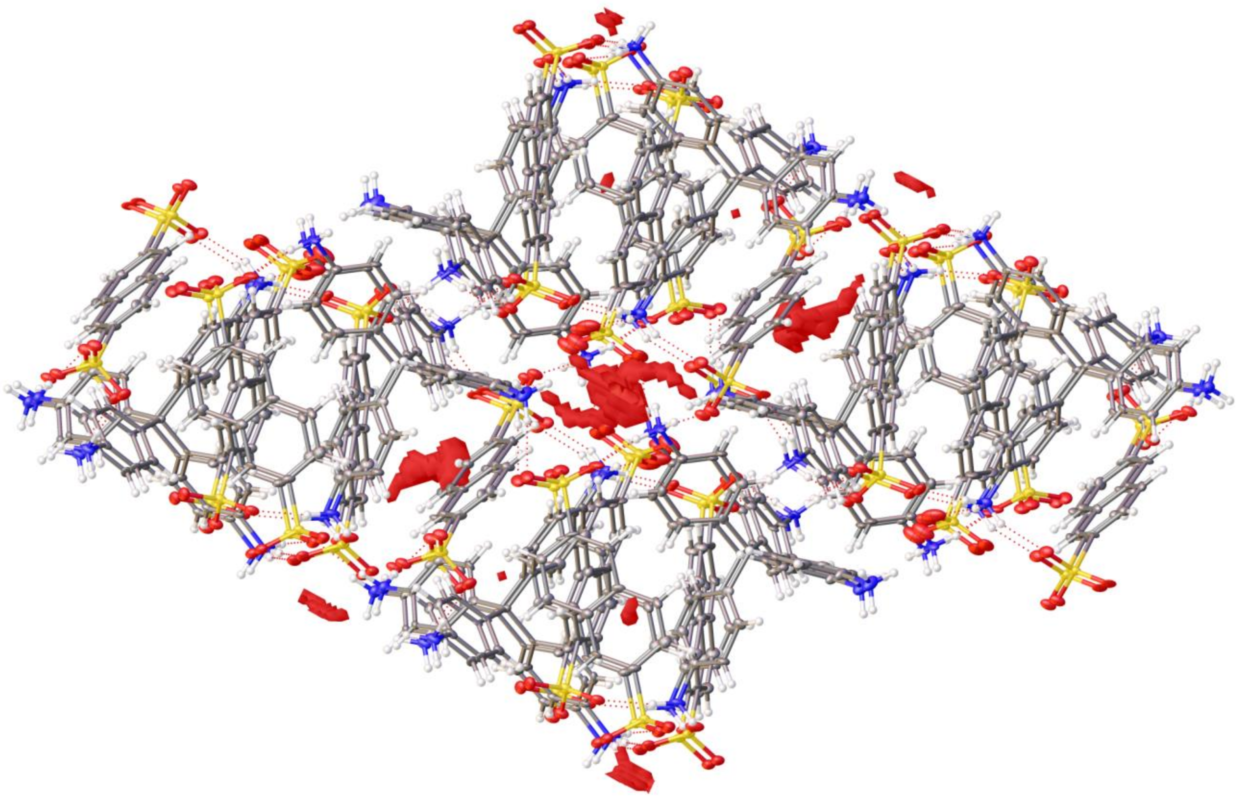

Figure S2. A fragment of the crystal packing in **F-1a'** showing solvent-accessible voids by red isosurfaces, as calculated by *calcvoid* routine implemented in Olex2[S1]; the volume of the largest spherical void is 11.49 Å^3.

PXRD patterns. Samples **F-1** and **F-1b** were placed between two polyimide films and mounted on a Bruker AXS D8 Advance Vario X-ray powder diffractometer equipped with primary monochromator (Ge111, Cu Kα1, λ = 1.54056 Å) and LynxEye PSD. Data were collected at room temperature. For **F-1**, the range was 2–90° 2θ. For **F-1b**, the range was 2–60° 2θ, and the sequence of short scans (≈20 min per full range) was performed to observe the phase transformations. The step size was 0.01° 2θ for both samples. The powder data were processed with Bruker Topas5 software. Phase composition of **F-1** was confirmed by Rietveld method (Figure S1). R values were: Rwp/Rwp'/Rp/Rp'/RBragg = 8.14/9.65/5.99/ 7.23/5.73%. Cell parameters of **F-1** were determined with Pawley method (Figure S2): *a* = 20.9609(10)Å, *b* = 19.7563(9)Å, *c* = 22.6642(10)Å, β = 92.694(3)°, *V* = 9375.1(8)Å3. The samples of **F-1b** were prepared from **F-1** by drying in vacuo at 100 °C for several hours placed between two polymide films. An additional effort was made to limit sample contact with air and water vapor. Film edges were greased with fluorinated grease and vacuumed. Diffraction data were collected in a sequence of short (about 20 minutes) runs. The first run is referenced as **F-1b start**. After 8 hours the diffraction pattern stopped changing. All the runs collected after that were summed up,

and the resulting pattern was referenced as **F-1b 8h**. We expected that without films and grease the phase transformations will be much faster. We were unable to index the pattern **F-1b start**; the forms of the diffraction peaks with close 2θ and different halfwidths suggested that the sample was a mixture (Figure S3). The phase composition of **F-1b 8h** was confirmed by Rietveld method (Figure S4). R values were: Rwp/Rwp'/Rp/Rp'/RBragg = 7.82/8.76/5.88/ 6.68/5.15%. Cell parameters of **F-1b 8h** were determined with Pawley method (Figure S5): a = 20.8677(13)Å, b = 20.0951(13)Å, c = 22.6324(15)Å, β = 92.432(5)°, V = 9482.1(11) Å^3. Significant differences in X-ray diffraction patterns of the two powder samples and relatively high R values of the Rietveld fits suggested that some structural parameters (such as water content) varied with the preparation method.

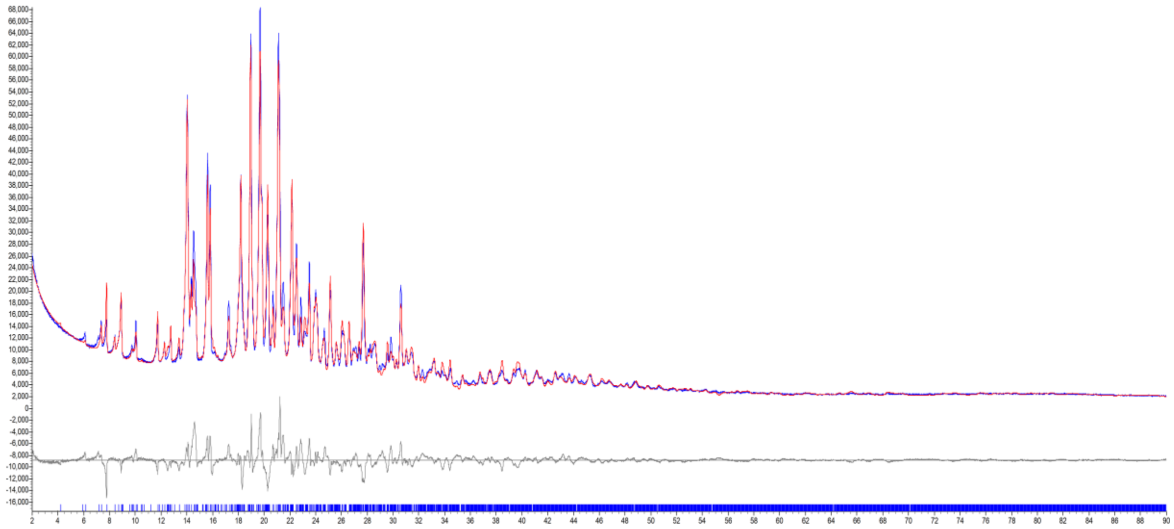

Figure S3. Observed (blue), calculated (red) and difference (grey) profiles for the Rietveld refinement of **F-1**.

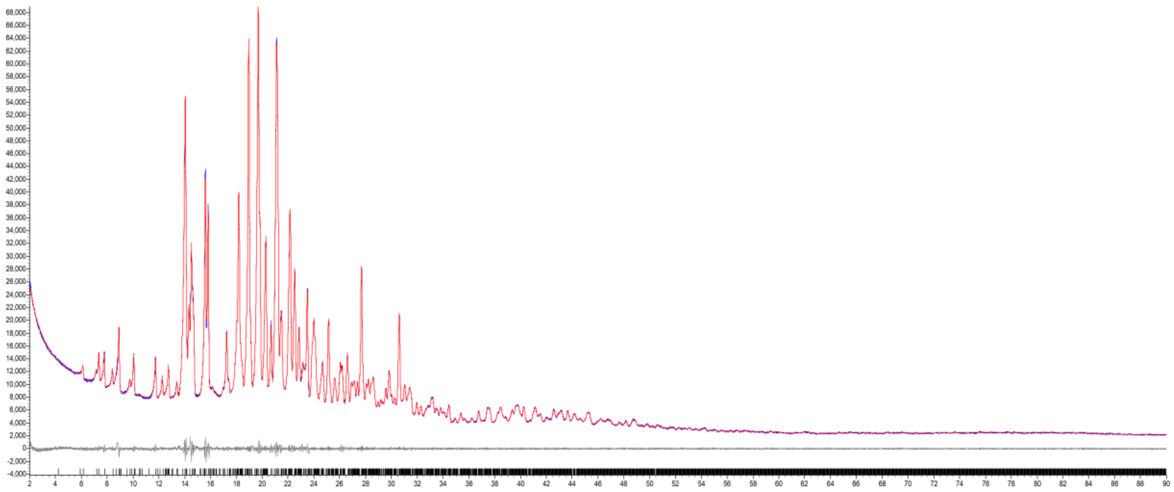

Figure S4. Observed (blue), calculated (red) and difference (grey) profiles for the Pawley refinement of **F-1**.

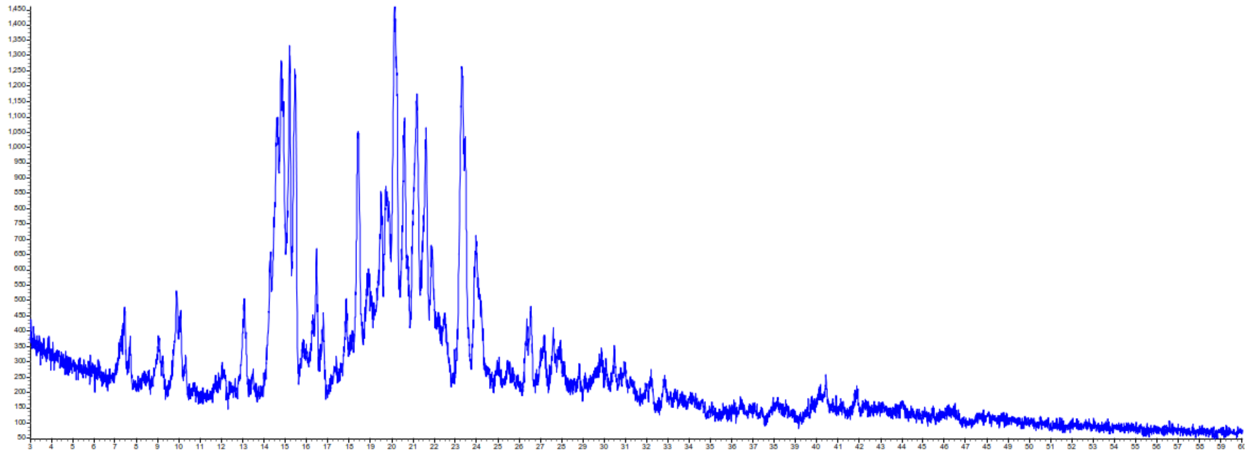

Figure S5. Observed (blue) profile for **F-1b start**.

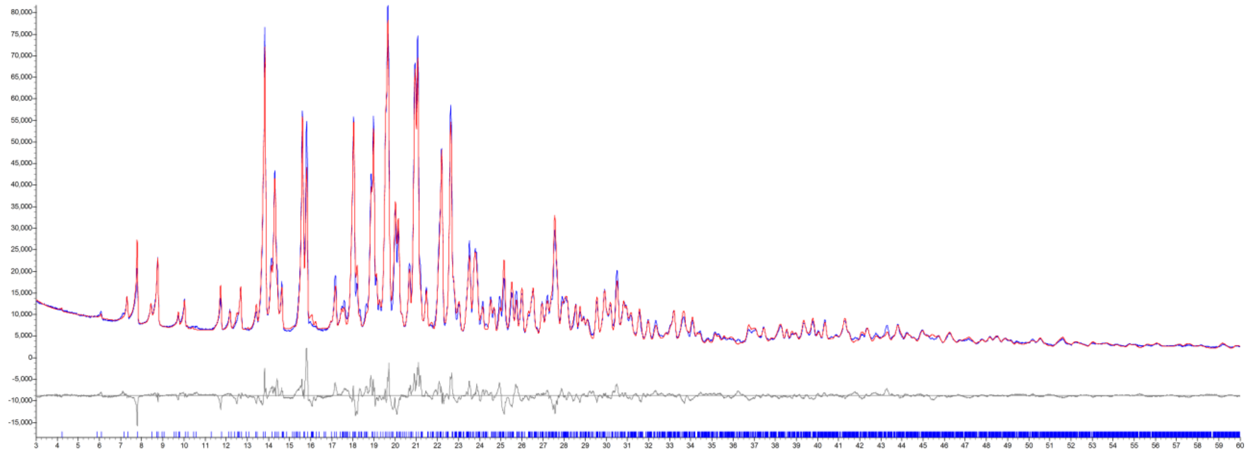

Figure S6. Observed (blue), calculated (red) and difference (grey) profiles for the Rietveld refinement of **F-1b 8h**.

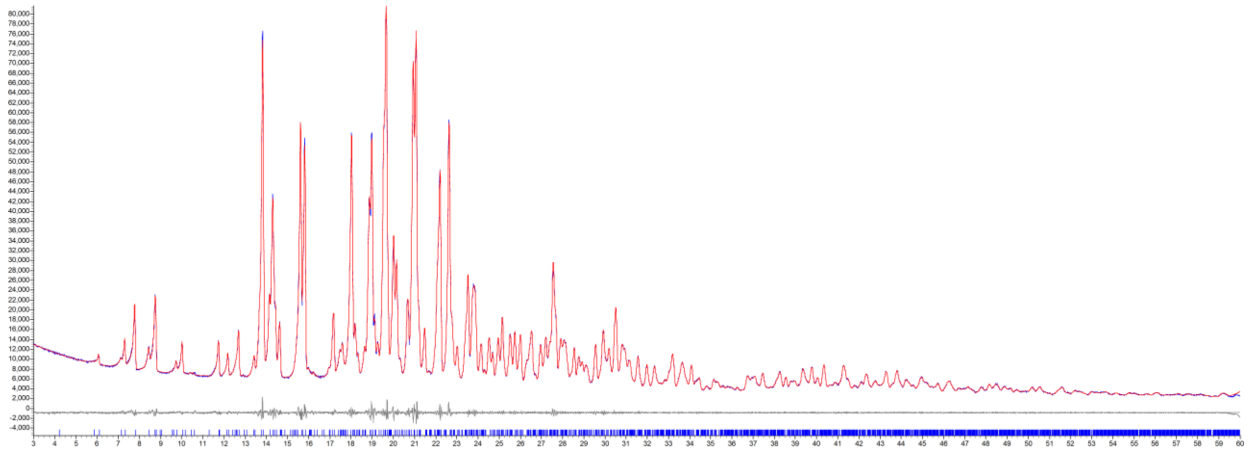

Figure S7. Observed (blue), calculated (red) and difference (grey) profiles for the Pawley refinement of **F-1b 8h**.

Calculation of the four pK_a's of TAPM. To check whether the four amonium groups of the aniline moieties affected the pK_a's of each other, pK_a's of all four protonated states of tetrakis(4-aminophenyl)methane were calculated. All calculations were carried out in Gaussian16 Revision A.03[S3] at M062X[S4]/def2TZVP[S5] and APFD[S6]/def2TZVP levels of theory, using the general methodology described in ref.[S7]. We used scaled SAS model[S8] with water as a solvent and parameters from[S7], which were obtained for M062X/6-31+G(d,p) level of theory. Proton solvation free energies were fitted individually for each level of theory to provide correct pK_a of aniline (4.61[S9]); the obtained values are: −267.086 and −270.097 kcal/mol for M062X and APFD, respectively. Calculated pK_a's are given in Table S4 and shown in Figure S8.

Table S4. The calculated pK_a's of different forms of protonated tetrakis(4-aminophenyl) methane.

step		method	
		APFD	M062X
first	(0–1)	5.29	5.00
second	(1–2)	4.68	4.52
third	(2–3)	4.14	4.04
fourth	(3–4)	3.88	3.82
aniline		4.61	4.61

According to Figure S8, pK_a's of all four transitions between the protonated states are quite close (within 1.3 pK_a units) at both used levels of theory, and we conclude that aniline fragments affect each other rather weakly. This makes the tetrakis(4-aminophenyl)methane the most condensed host of weakly interacting aniline moieties.

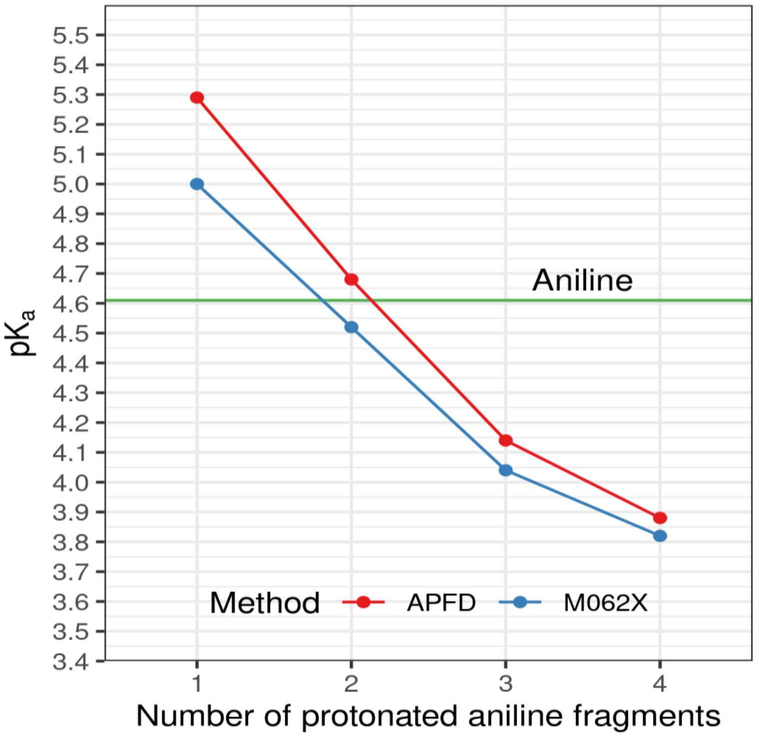

Figure S8. The calculated pK_a's of different forms of protonated tetrakis(4-aminophenyl)-methane.

SEM images were obtained with a scanning electron microscope Hitachi TM-1000 with a detector TM1000 EDS, allowing to run local roentgen-spectral analysis (speeding voltage – 15 kV). The sulfur atom uniformity of distribution was assessed and proved for different parts of a single particle with a precision of 0.15–0.20%.

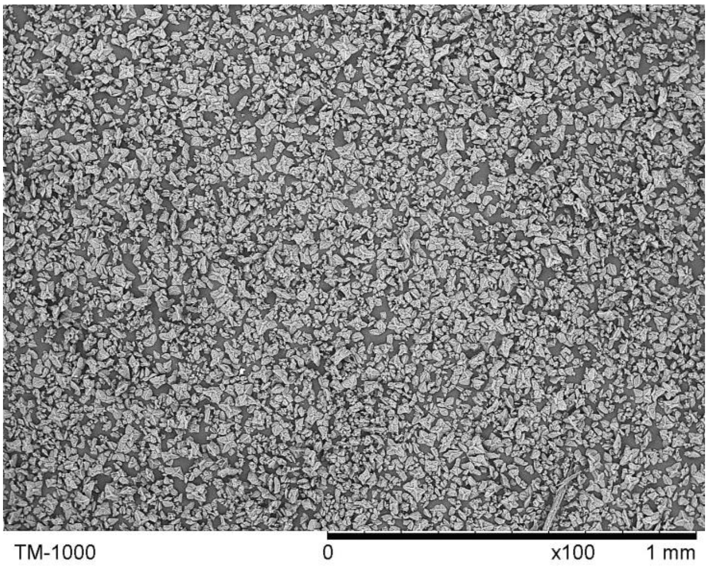

Figure S9. The particles of **F-1** precipitated from the mixture of TAPM and NDS in water.

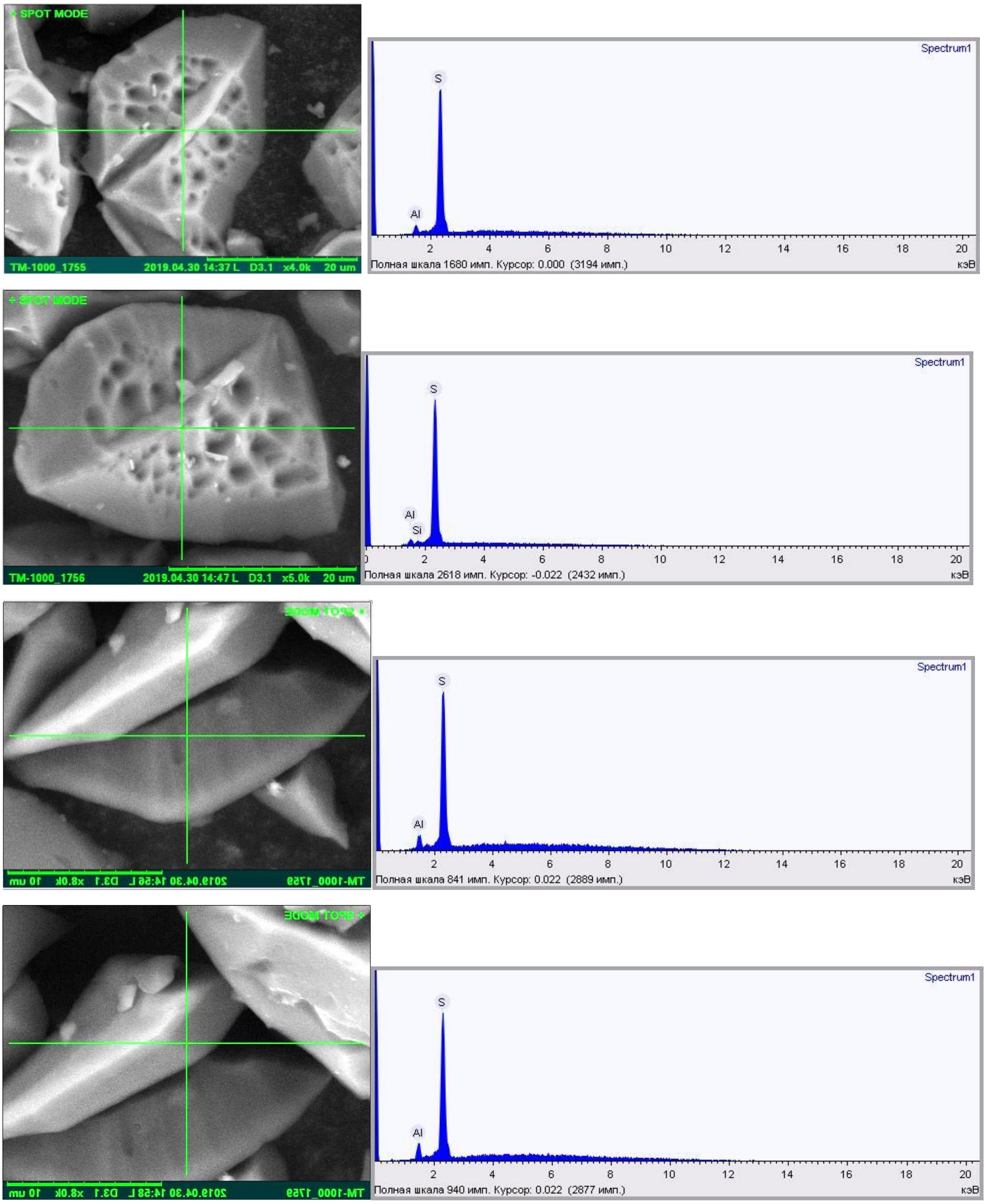

a) b)

Figure S10. Sulfur atom distribution according to local roentgen-spectral analysis. a) The spot at the particle where the determination was made. b) The spectra of the characteristic roentgen radiation.

TGA analysis experiments were run on SDT Q600 V20.5 Build 15 «TA Instruments» (USA) equipped with IR – Furrier analyzer of the evolving gases. The samples were heated at a rate of 20 degrees / minutes in an Al_2O_3 crucible.

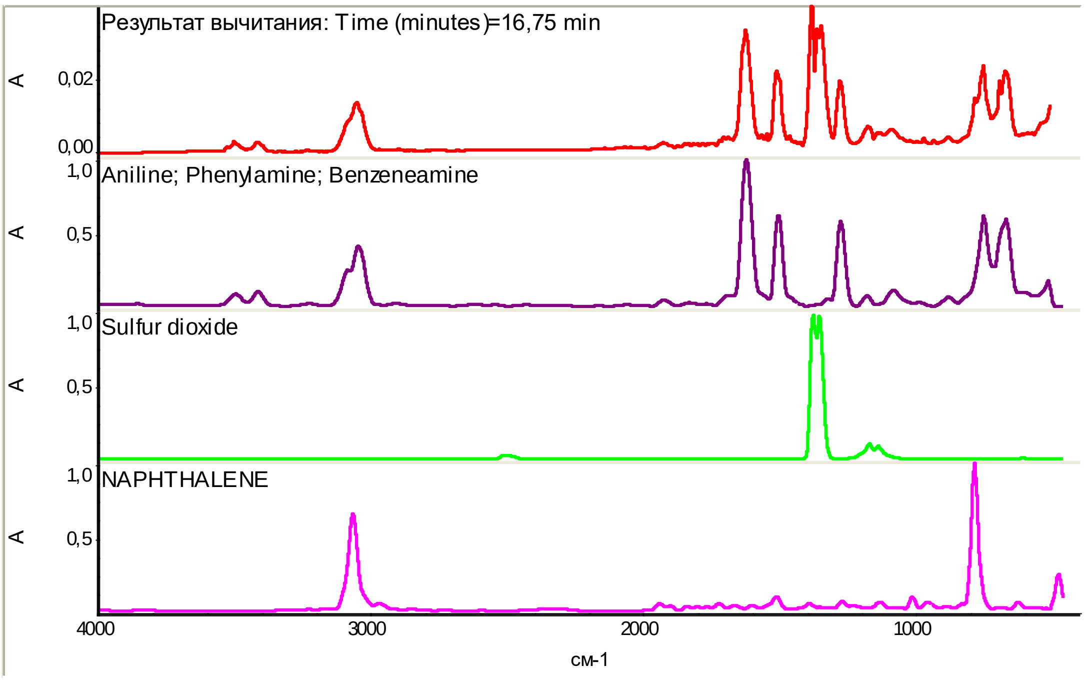

Figure S11. The results of IR spectroscopy of the evolving gases, 16.75 min after the heating was commenced.

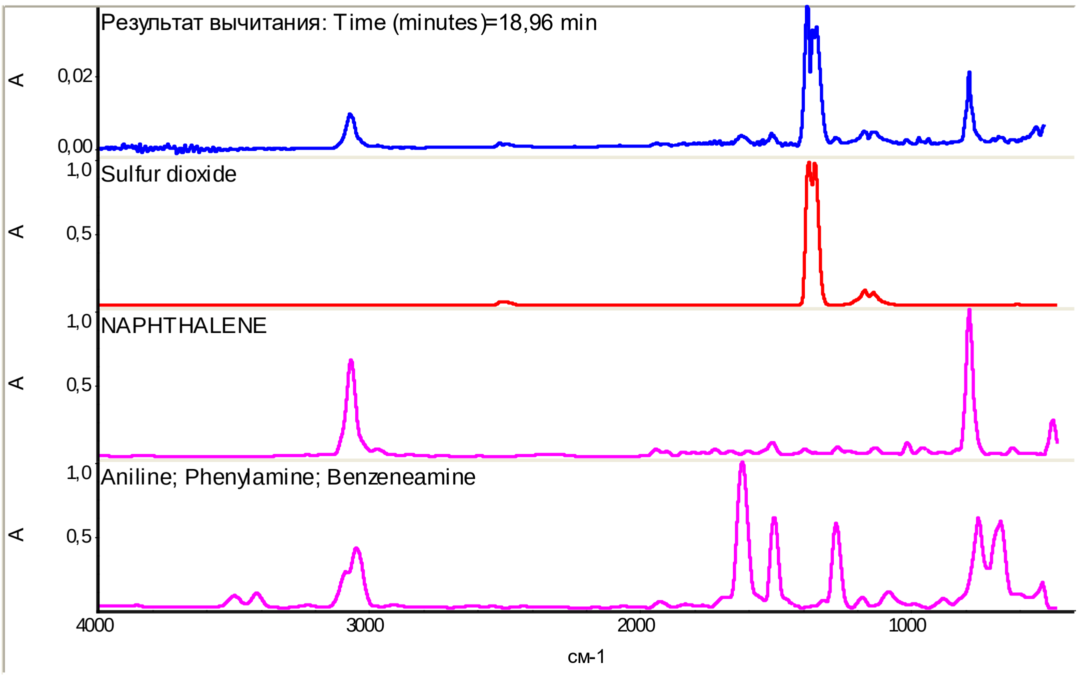

Figure S12. The results of IR spectroscopy of the evolving gases 18.96 min after the heating was commenced.

3. Synthesis of F-1

1.50 g (3.95 mmol) of tetrakis(4-aminophenyl)methane was protonated using 48 equiv of HCl aq. solution acid (18.5%, 36 mL) and the mixture was evaporated at a reduced pressure to remove the excess HCl. The resulted TAPM was well soluble in water and **F-1** was prepared by simple fast mixing of its aqueous solution (2.07 g, 3.95 mmol in 50 mL of H_2O) with an aqueous solution of the disodium salt of NDS (2.62 g, 7.9 mmol in 50 mL of H_2O) under vigorous stirring. A white precipitate was formed immediately and the stirring was continued for 24 hours, then the precipitate was filtered, washed with H_2O and dried over P_2O_5 to give **F-1** in a yield of 95% (3.59 g, 3.75 mmol). M.p. >300ºC.^{1}H NMR (400 MHz, DMSO-d$_6$, 25 °C): δ = 8.20 (s, 4H), 7.97 (d, J=8.5 Hz, 4H), 7.76 (d, J=8.4 Hz, 4H), 7.27 (d, J=8.4 Hz, 8H), 7.16 (d, J=8.3 Hz, 8H) ppm. ^{13}C NMR (101 MHz, DMSO-d$_6$, 25 °C): δ = 145.67, 132.48, 131.76, 130.43, 128.64, 124.64, 124.50, 123.36, 63.81 ppm. Anal. Calcd for $C_{45}H_{40}N_4O_{12}S_4$*$3H_2O$: C, 53.45; H, 4.59; N, 5.54; S, 12.68; Found: C, 53.60, H, 4.61; N, 5.79; S, 12.86. IR (KBr): ν = 2854(NH_3^+), 2601(C-H), 1172 (SO_3^-).

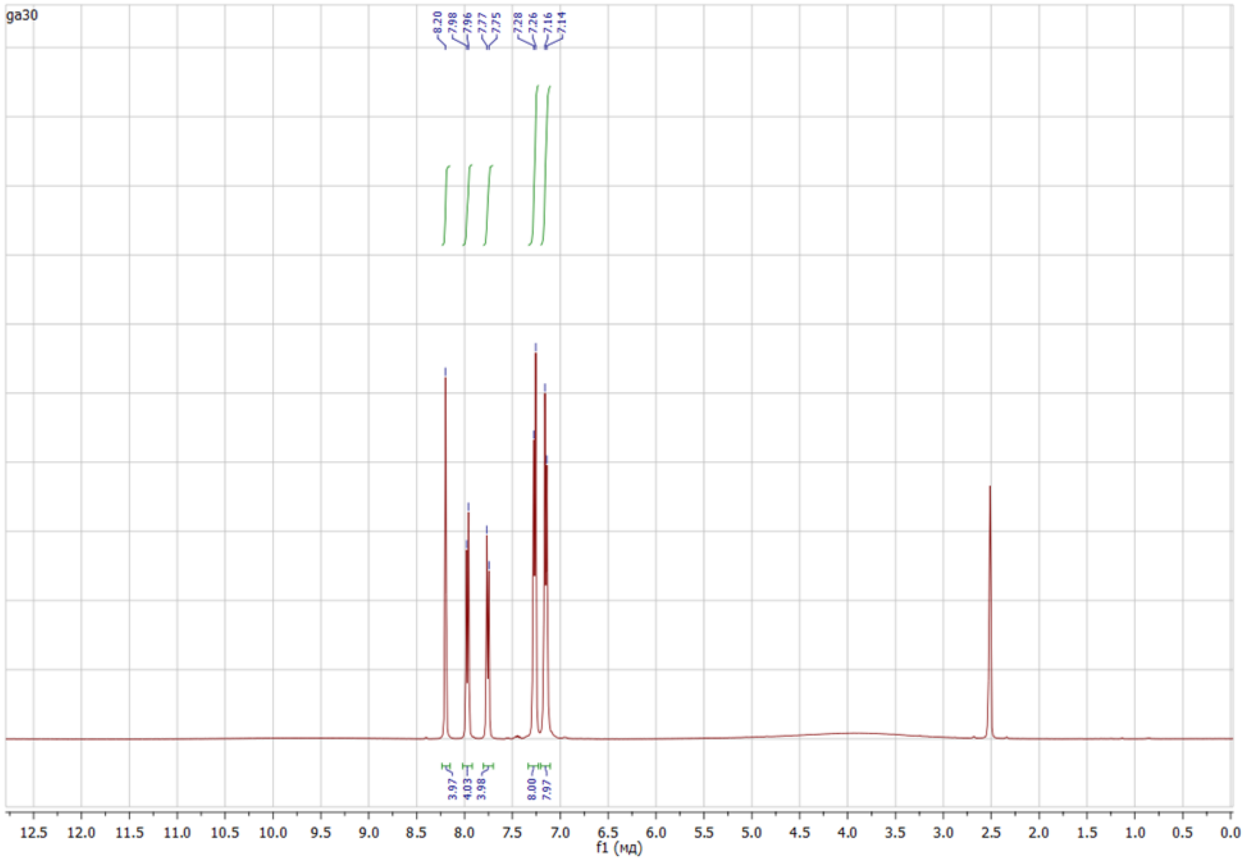

Figure S13. ^{1}H NMR (400 MHz) spectra of **F-1** in DMSO-d_6.

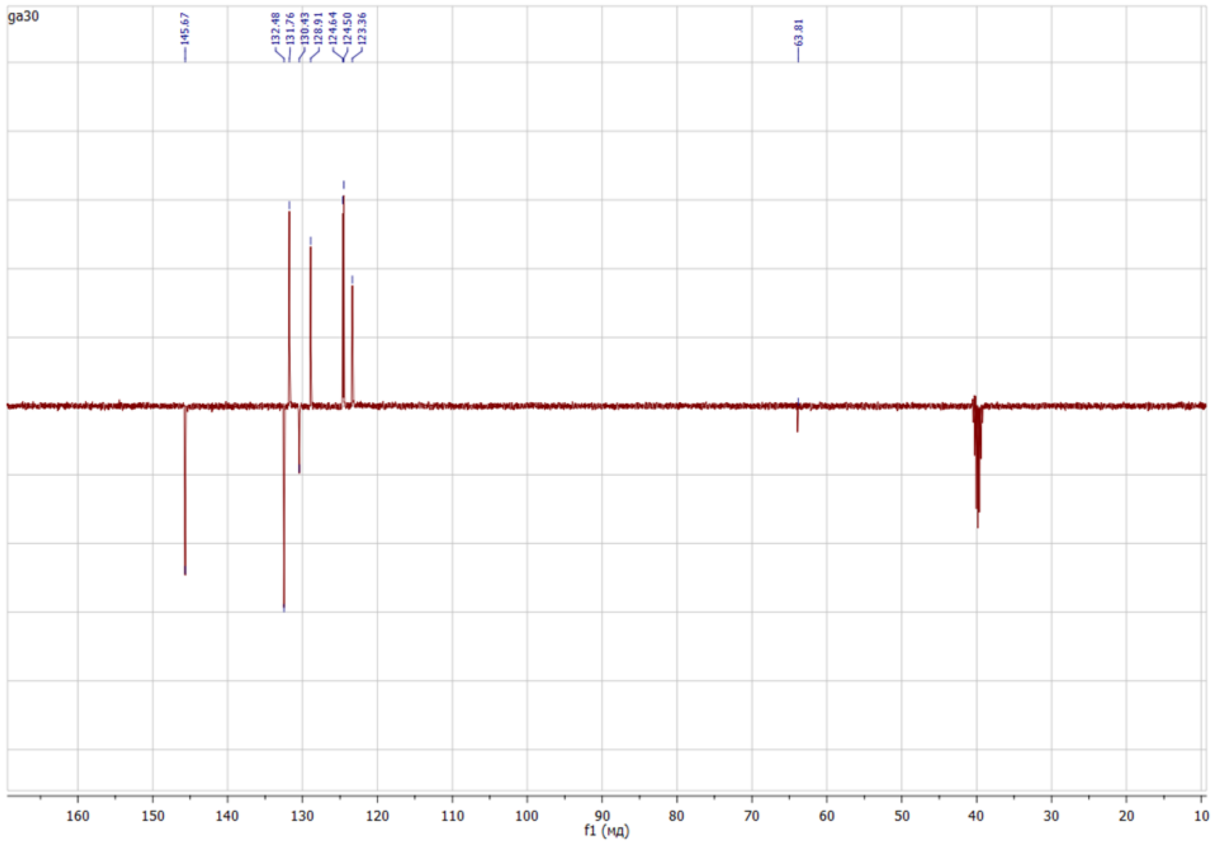

Figure S14. ^{13}C NMR spectrum of **F-1** in DMSO-d_6.

4. Opening of styrene oxide (2) with MeOH

To a solution of **2** (1.1 g, 9.16 mmol) in 50 mL of MeOH was added **F-1** (0,11 g, 0.12 mmol). As one unit of framework contained 4-ammonium groups, the real amount of the catalytic units corresponded to 5 mol % relative to the substrate. The heterogeneous reaction mixture was stirred for 1 hour at a room temperature and then the catalyst was filtered. The filtrate was evaporated and, according to its NMR spectra, did not contain any unreacted **2**. The residue was filtered through a pad of SiO_2 (EtOAc/hexane, 1/1) and the filtrate evaporated to give **3** as a single isomer in a yield of 80% (1.11 g, 7.3 mmol). ^1H NMR (400 MHz, $CDCl_3$, 25 °C): δ = 7.48-7.26 (m, 5H), 4.34 (dd, J=3.86, 8.41, 1H), 3.68 (ddd, J=8.48, 11.59, 19.56, 2H), 3.34 (s, 3H), 2.28 (br. s, 1H) ppm. The spectrum was identical to the earlier described one for **3**[S10] and differed from that for its β-isomer.[S11] ^{13}C NMR (101 MHz, $CDCl_3$, 25 °C): δ = 138.44, 128.56, 128.23, 126.94, 84.92, 67.28, 56.89. m/z 175.0731 calculated for $C_9H_{12}O_2$ $[M+Na]^+$ was m/z: 175.08

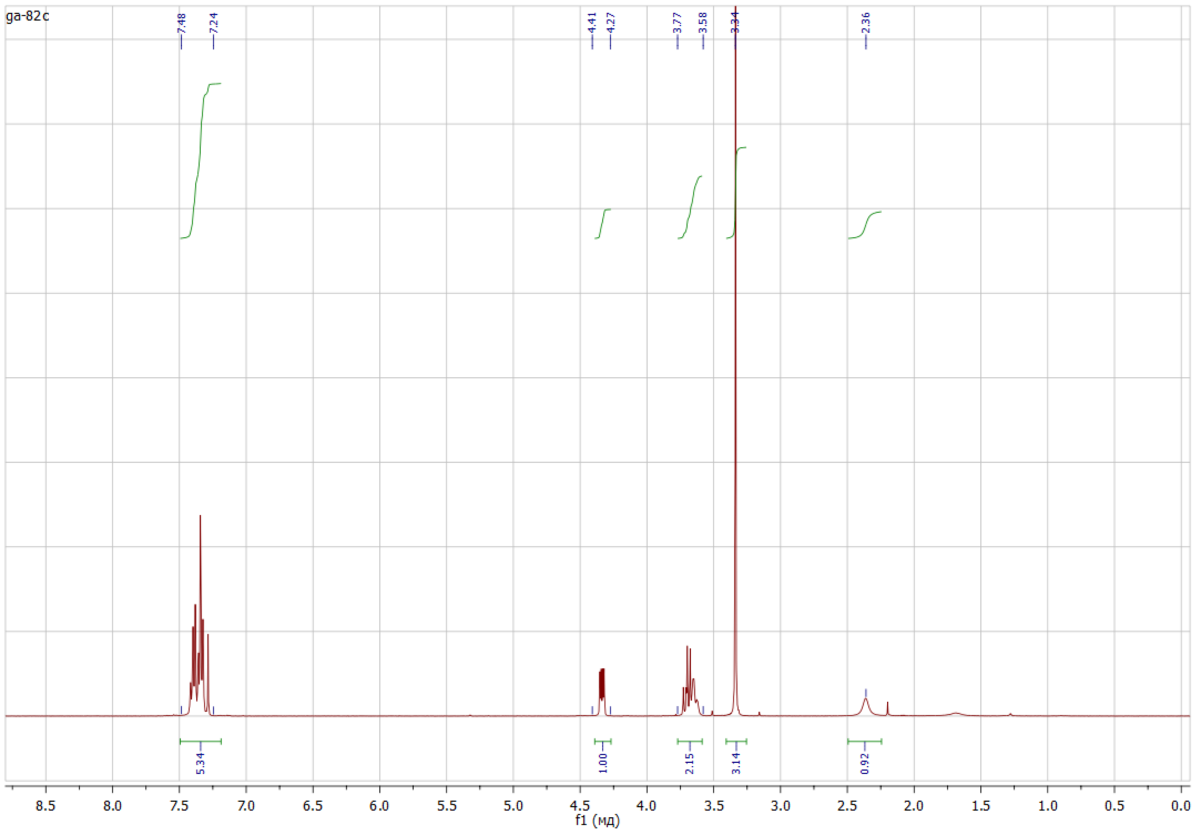

Figure S15. ^{1}H NMR (400 MHz) spectra of **3**.

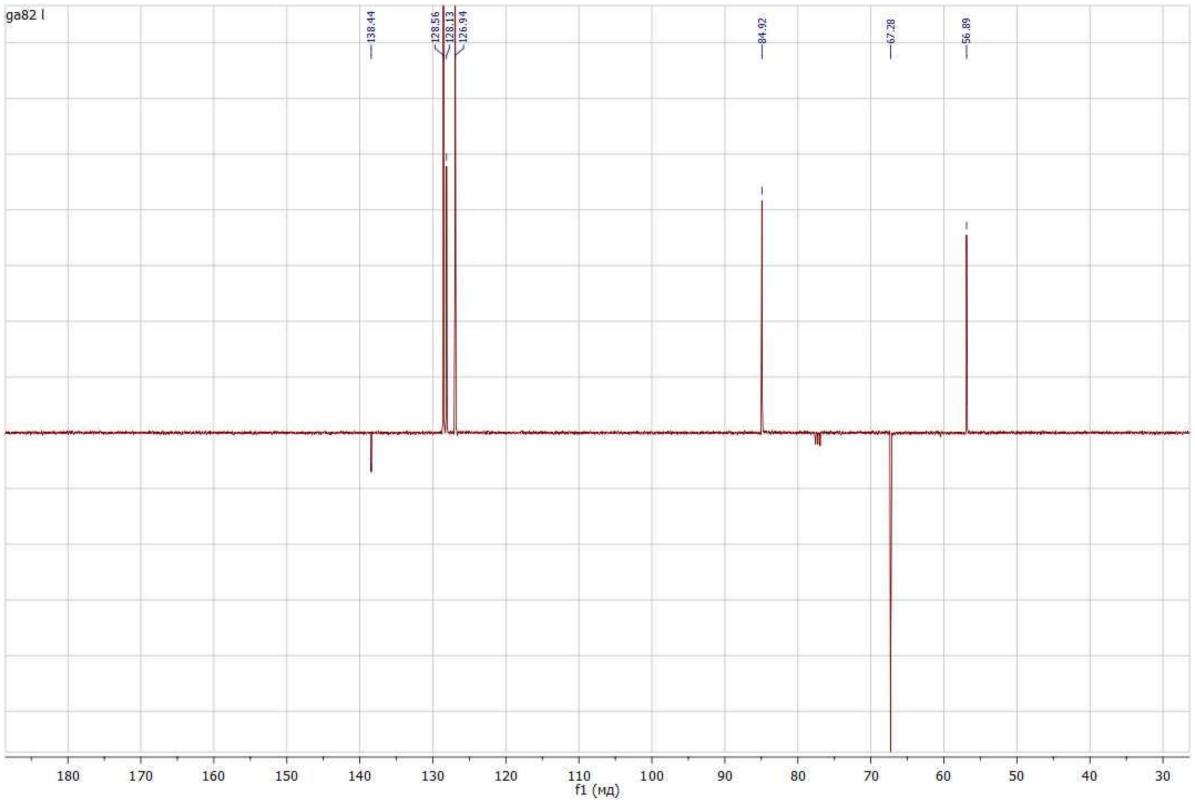

Figure S16. ^{13}C NMR spectrum of **3** in $CDCl_3$.

The stability of the catalyst was checked in a series of experiments and the spectrum of the recovered catalyst after 5-th run is shown in Figure S17.

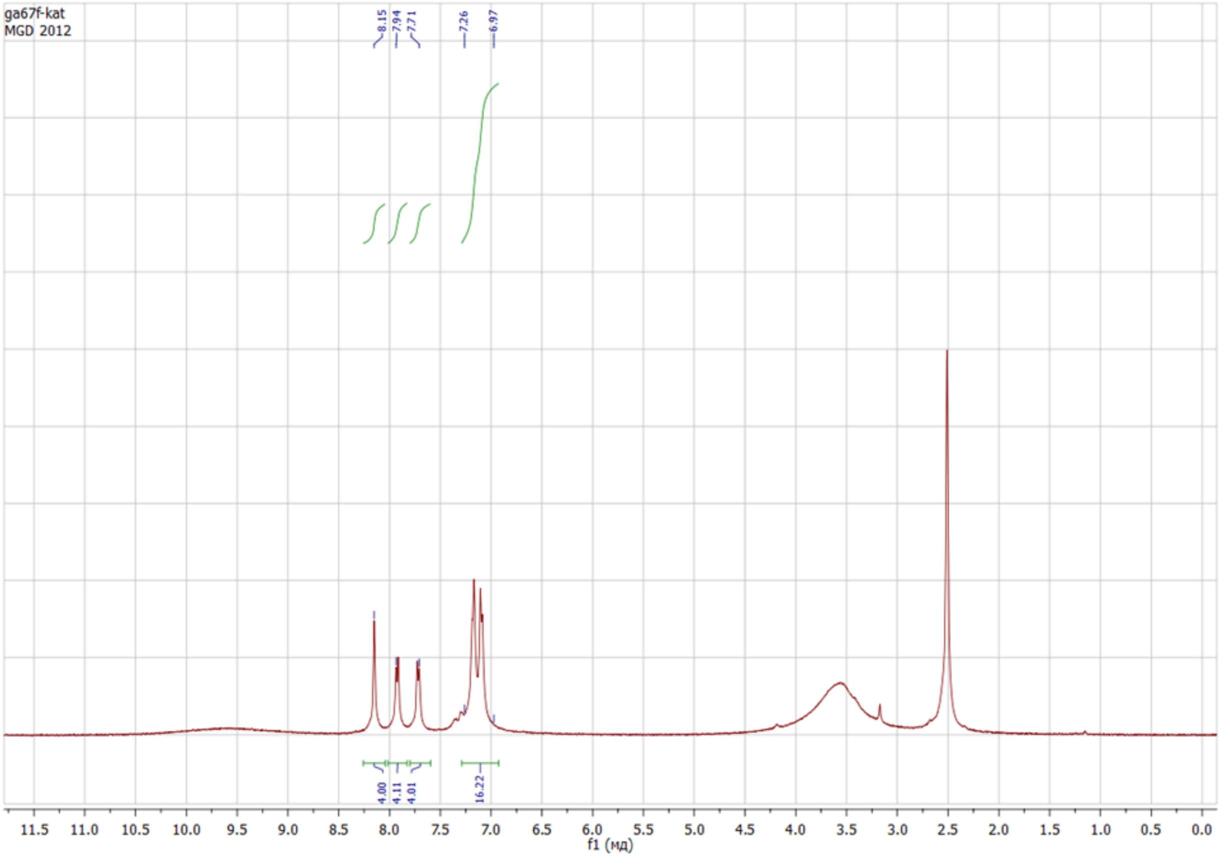

Figure S17. ^{1}H NMR (400 MHz) spectra in DMSO-d_6 of **F-1** recovered after after 5 cycles of **2** to **3** conversion in neat MeOH.

The same reaction was conducted in a mixture of CH_2Cl_2 and MeOH at room temperature. **2** (0.2 mL, 1.83 mmol), MeOH (1.48 mL, 36.6 mmol), CH_2Cl_2 (10 mL), **F-1** (0.023 g) were mixed and stirring (700 rpm) was continued for 24 hours. After 24 hours the mixture was filtered and the filtrate was evaporated. The ratio of **2** to **3** was determined by ^{1}H NMR spectra.

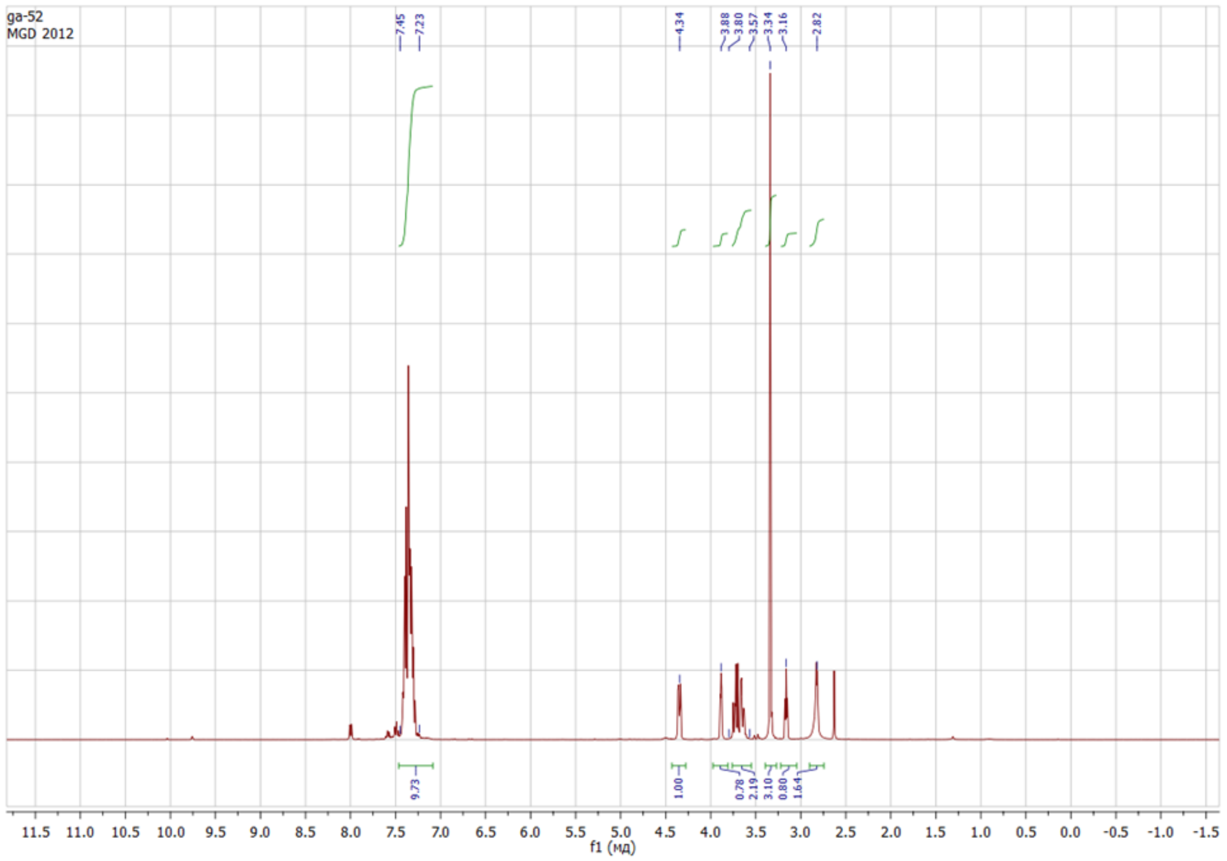

Figure S18. ^{1}H NMR spectrum ($CDCl_3$) of a mixture of **2** and **3** obtained in an experiment run in a CH_2Cl_2/MeOH solution promoted by **F-1**.

5. Opening of styrene oxide (2) with H_2O.

The catalyst **F-1** (0.23 g) was added to a solution of styrene oxide (2 mL, 1.83×10^{-2} mol) in 50 mL of CH_2Cl_2 with stirring, then 100 mL of twice distilled H_2O was added, and the mixture stirred (700–1400 rpm) for 24 h at rt. Then the mixture was filtered to remove the insoluble catalyst (0.14 g) and the layers were separated. The aqueous layer was evaporated, the residue taken up in a portion of CH_2Cl_2 and filtered to remove the insoluble catalyst (0.09 g). The filtrate was evaporated to give 1-phenylethane-1,2-diol (**4**, 1.8 g, 1.3×10^{-2} mol, 72%). The organic layer was evaporated to give additionally 23% (0.55 g, 2.1×10^{-3} mol) of **4**. ^{1}H NMR (400 MHz, $CDCl_3$, 25 °C): δ = 7.44-7.17 (m, 5H), 4.77 (dd, J=3.26, 8.48 Hz, 1H), 3.84 (s, 2H), 3.65 (ddd, J=5.96, 11.57, 20.10, 2H) ppm. ^{13}C NMR (101 MHz, $CDCl_3$, 25 °C): δ = 140.49, 128.49, 127.90, 126.13, 74.75, 68.02 ppm. M.P.= 65-67°C (Lit. 67°C)[S12] and also the ^{1}H NMR and ^{13}C NMR-spectra are identical to those described earlier.[S12] m/z 161.0639, calculated for $C_8H_{10}O_2$ $[M+Na]^+$ 161.07. Anal. Calcd for $C_8H_{10}O_2$: C, 69.54; H, 7.30; Found: C, 69.93, H, 7.20.

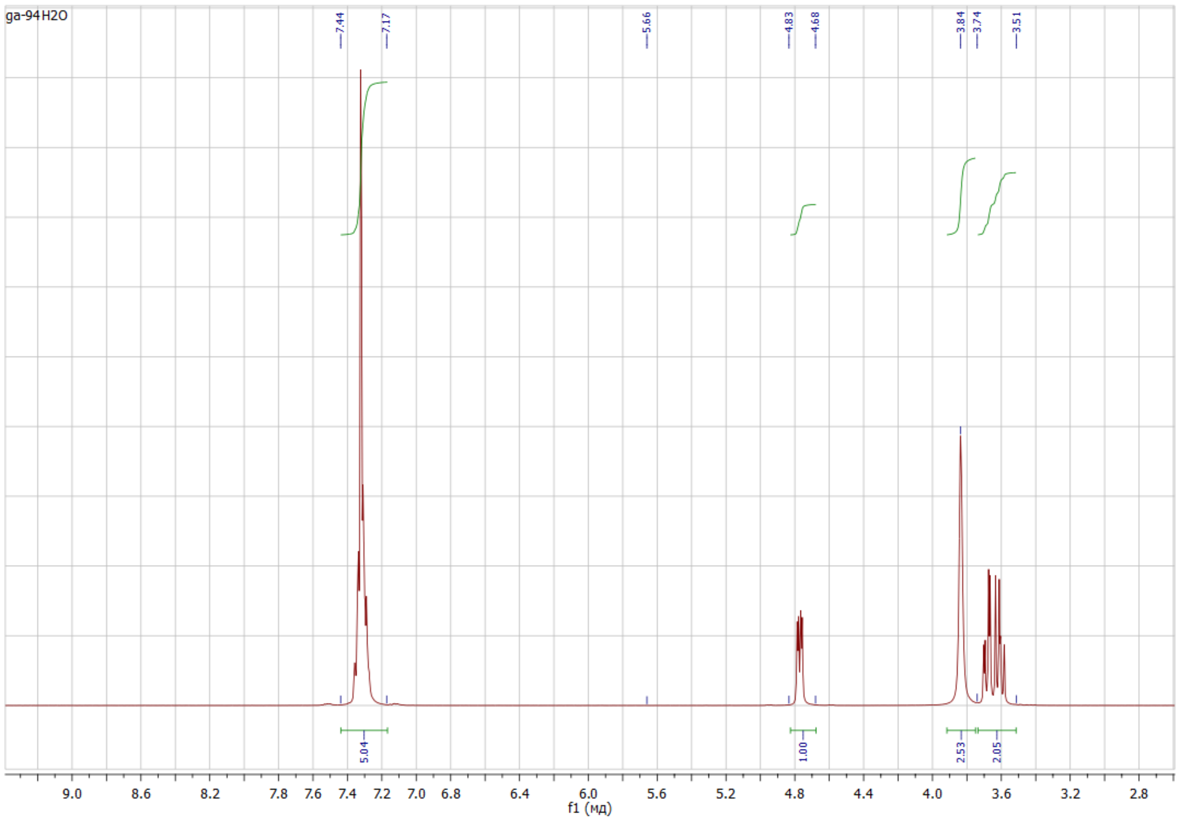

Figure S19. ^{1}H NMR (400 MHz) spectra of **4** in $CDCl_3$.

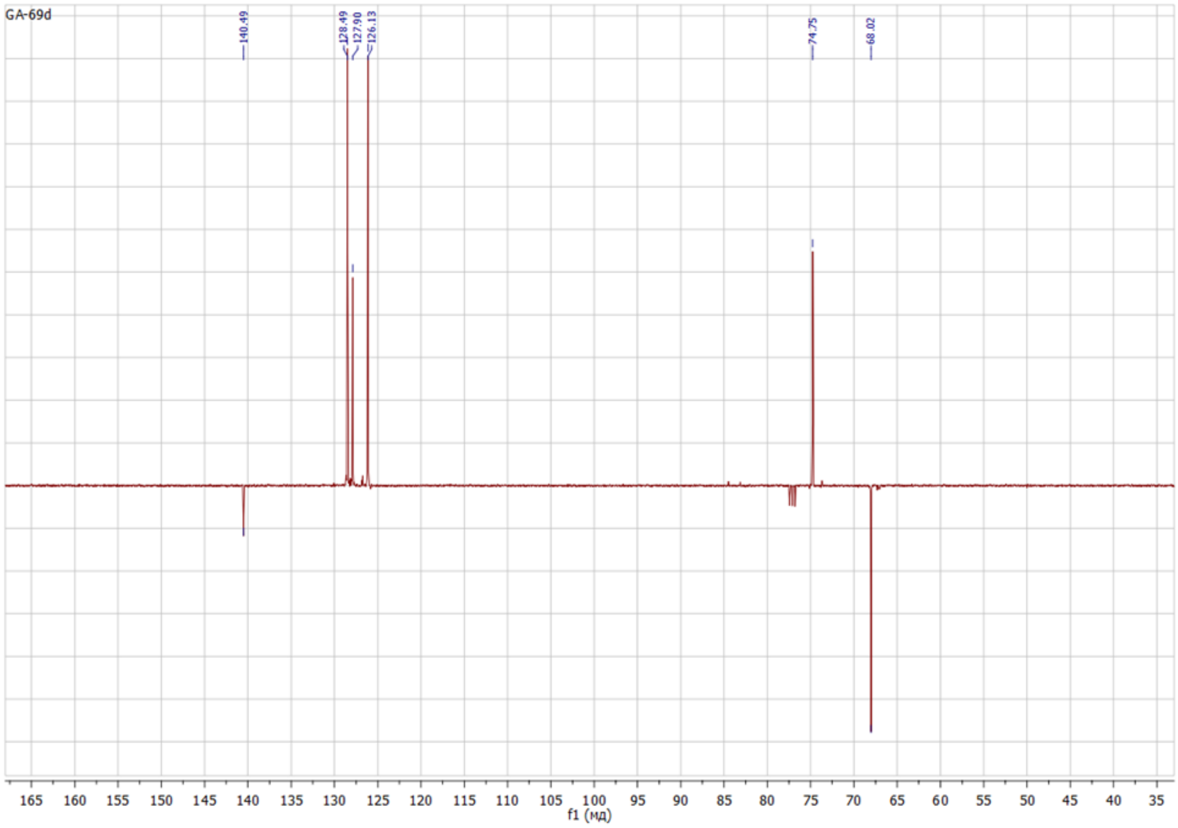

Figure S20. ^{13}C NMR (101 MHz) of **4** in $CDCl_3$.

Catalysis by leached **F-1**. The catalyst (0.11 g) was added to a stirred mixture of 25 mL of CH_2Cl_2 and 50 mL of twice distilled H_2O, and the resulting mixture stirred (1000 rpm) for 15 min at rt. Then the catalyst was filtered through a dense ceramic filter. To the resulting transparent two-layer mixture was added styrene oxide (**2**, 1 mL, 1.1 g, 9.17×10^{-3} mol) and the mixture stirred for 3 hours (700 rpm). The layers were separated and each was evaporated distinctly. The aqueous residue contained 35% (0.45 g, 3.2×10^{-3} mol) of **4**. The organic layer contained 60% (0.667 g, 5.56×10^{-3} mol) of **2** and 0.056 g (4.06×10^{-4} mol, 4%) of **4**.

6. Opening of *trans*-cyclohexeneoxide (5) with water.

To a solution of cyclohexene oxide (0.92 g, 9.38×10^{-3} mol) in 25 mL of CH_2Cl_2 was added **F-1** (0.11 g) and then 50 mL of H_2O with stirring (700 rpm). The stirring was continued for another 24 hours at a room temperature and then filtered. The transparent organic and aqueous layers were separated and each was evaporated. The aqueous layer residue was dissolved in CH_2Cl_2 and the mixture was filtered to remove the remaining catalyst. The evaporation of the filtrate gave cyclohexane-1,2-diol in a yield of 80% (0.87 g, 7.5×10^{-3} mol). M.p. 104-106°C (lit 107.5-108.5°C),[S13] m/z 139.0735, calculated for $C_6H_{12}O_2$, $[M+Na]^+$ m/z: 139.08. ^1H NMR (400 MHz, $CDCl_3$, 25 °C): δ = 4.05 (br.s, 2H), 3.41-3.23 (m, 2H), 2.05-1.84 (m, 2H), 1.79-1.56 (m, 2H), 1.35-1.12 (m, 4H) ppm. ^{13}C NMR (101 MHz, $CDCl_3$, 25 °C): δ = 75.64, 32.90, 24.36 ppm. The spectra corresponded to those assigned to *trans*-cyclohexane-1,2-diol.[S14] The CH_2Cl_2 solution was also evaporated and the spectra revealed absence of any epoxide. The evaporation of the solvent gave additionally another 0.1 g (8.6×10^{-4} mol, 9% mol) of **6**.

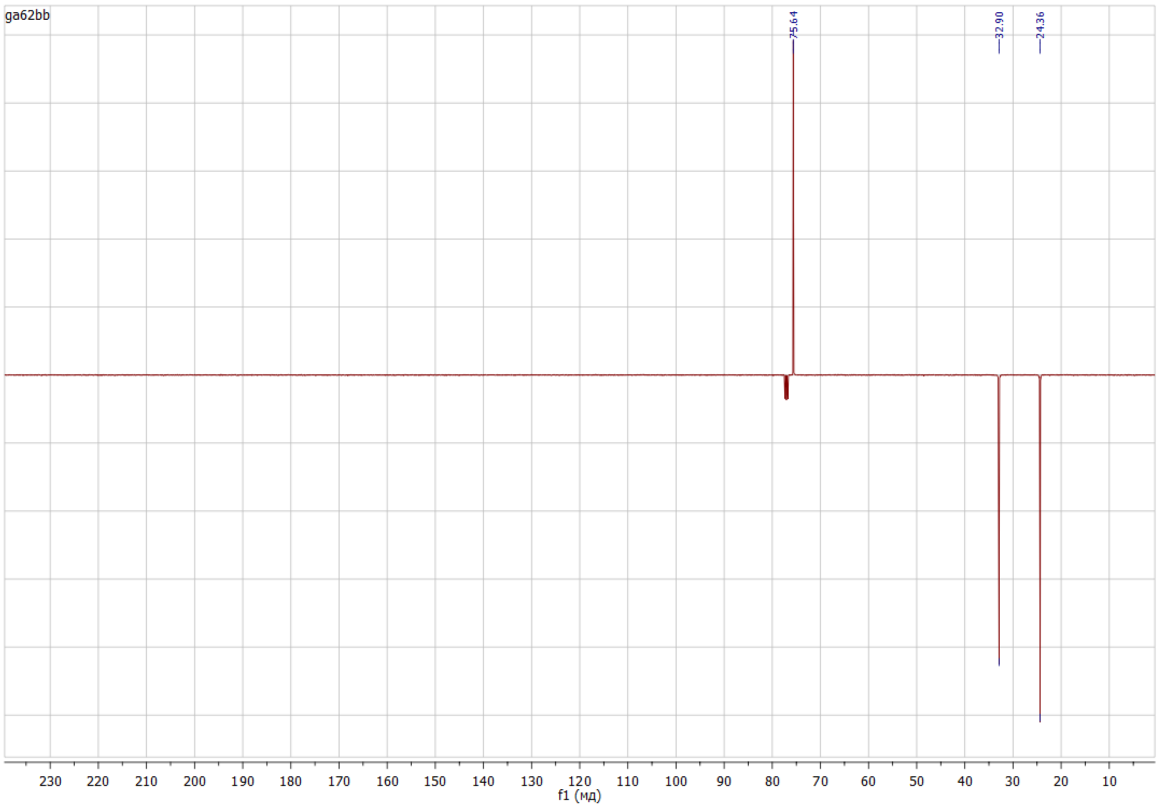

Figure S21. ^{13}C NMR (101 MHz) of **6**.in $CDCl_3$.

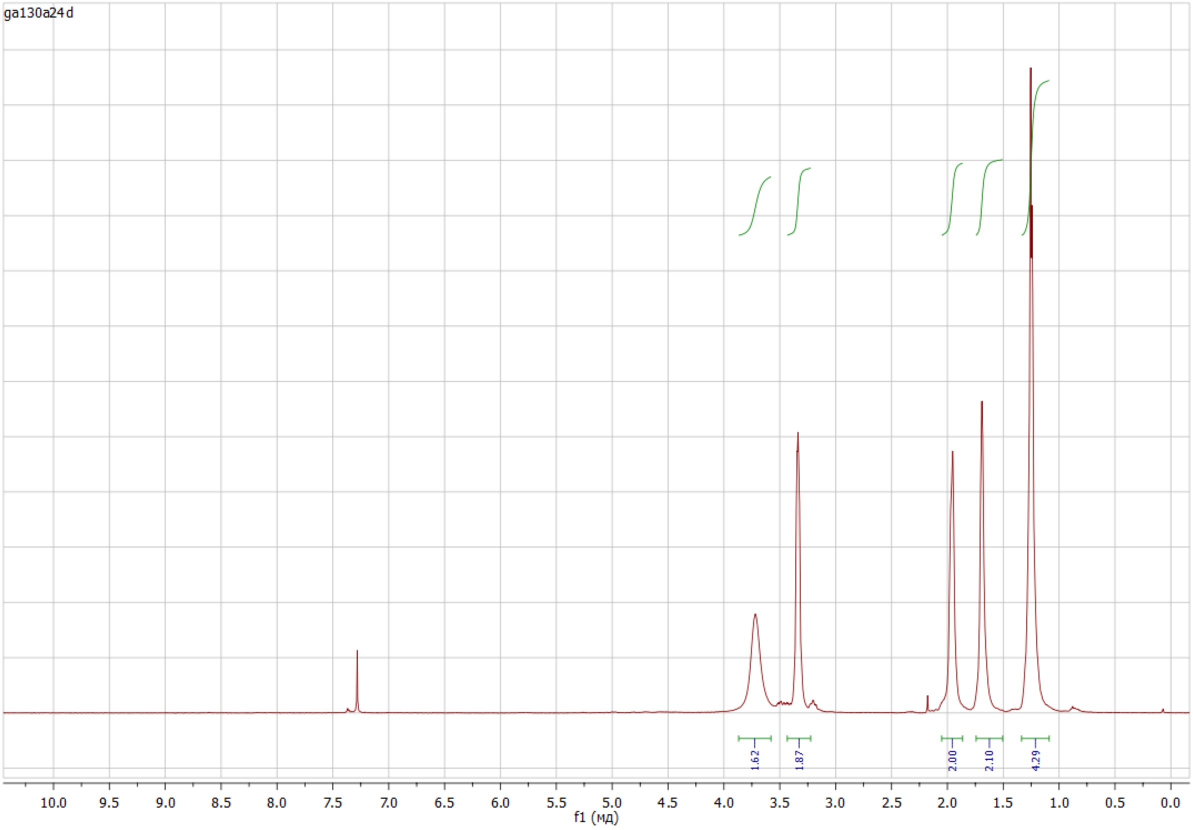

Figure S22. ^{1}H NMR (400 MHz) spectra of **6** recovered from the CH_2Cl_2-solution after its evaporation.

7. Opening of *trans*-cyclohexeneoxide (5) with alcohols

7.1 Opening of *trans*-cyclohexeneoxide (5) with MeOH. To a solution of **5** (0.185 mL, 1.83 × 10^{-3} mol) in MeOH (10 mL) was added **F-1** (0.023 g, 2.4 × 10^{-5} mol). The reaction mixture was stirred (700 rpm) for 4 h at room temperature. The insoluble **F-1**was removed by filtration and the filtrate evaporated. To the residue were added *p*-dinitrobenzene (as a standard, 0.009 g, 5.3 × 10^{-5} mol) and $CDCl_3$ (1 mL). The remaining insoluble in $CDCl_3$ **F-1**was filtered and the ^1H and ^{13}C NMR spectra of the residue were run (Figure S23 and S24). The spectra corresponded to the literature data.[S15] According to the data the chemical yield of the final product was >98%. ^1H NMR (400 MHz, DMSO-d_6, 25 °C): δ = 3.42-3.28 (m, 4H), 3.13 (br.s, 1H), 2.94-2.82 (m, 1H), 2.12-1.99 (m, 1H), 1.99-1.88 (m, 1H), 1.74-1.54 (m, 2H), 1.30-1.09 (m, 3H), 1.09-0.94 (m, 1H) ppm. ^{13}C NMR (101 MHz, DMSO-d_6, 25 °C): δ = 84.83, 73.51, 56.04, 32.12, 28.14, 23.91 ppm.

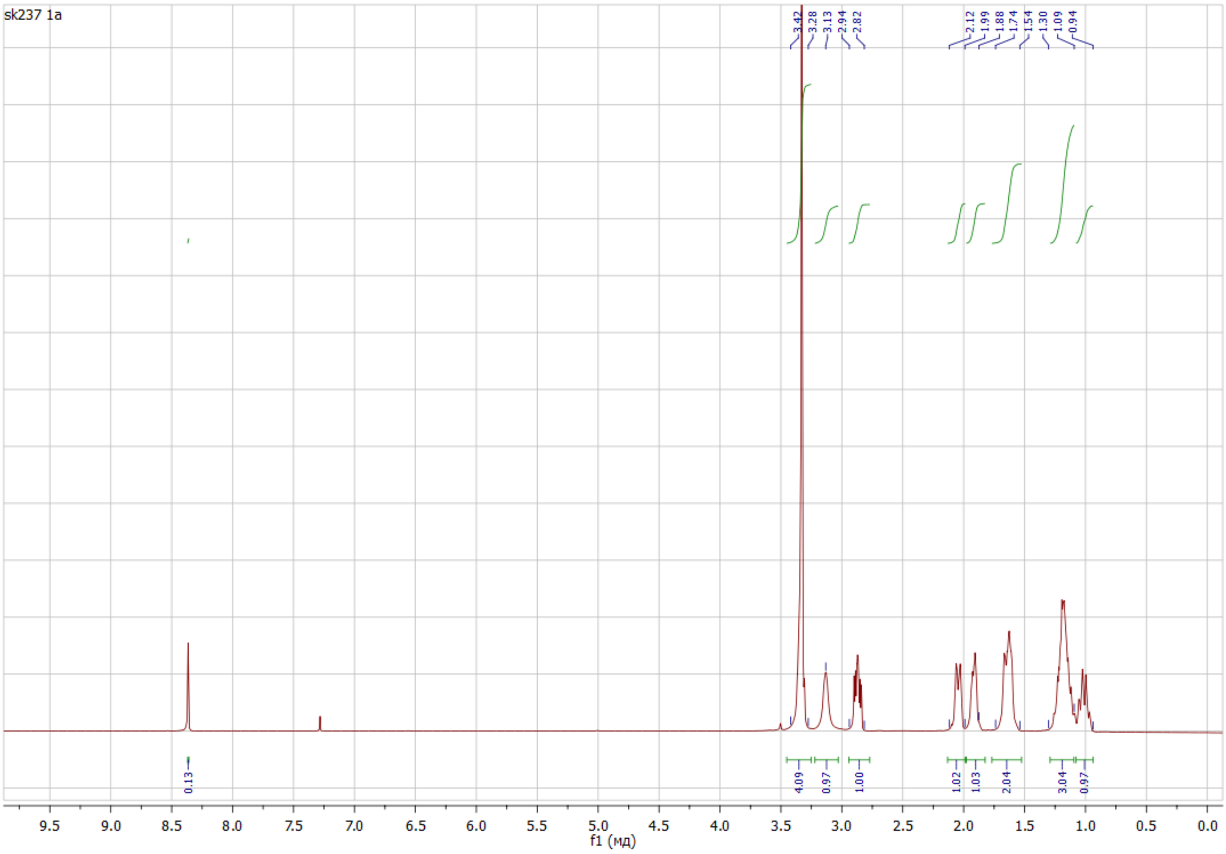

Figure S23. ^1H NMR (400 MHz) spectra of 2-methoxycyclohexanol-1 in $CDCl_3$.

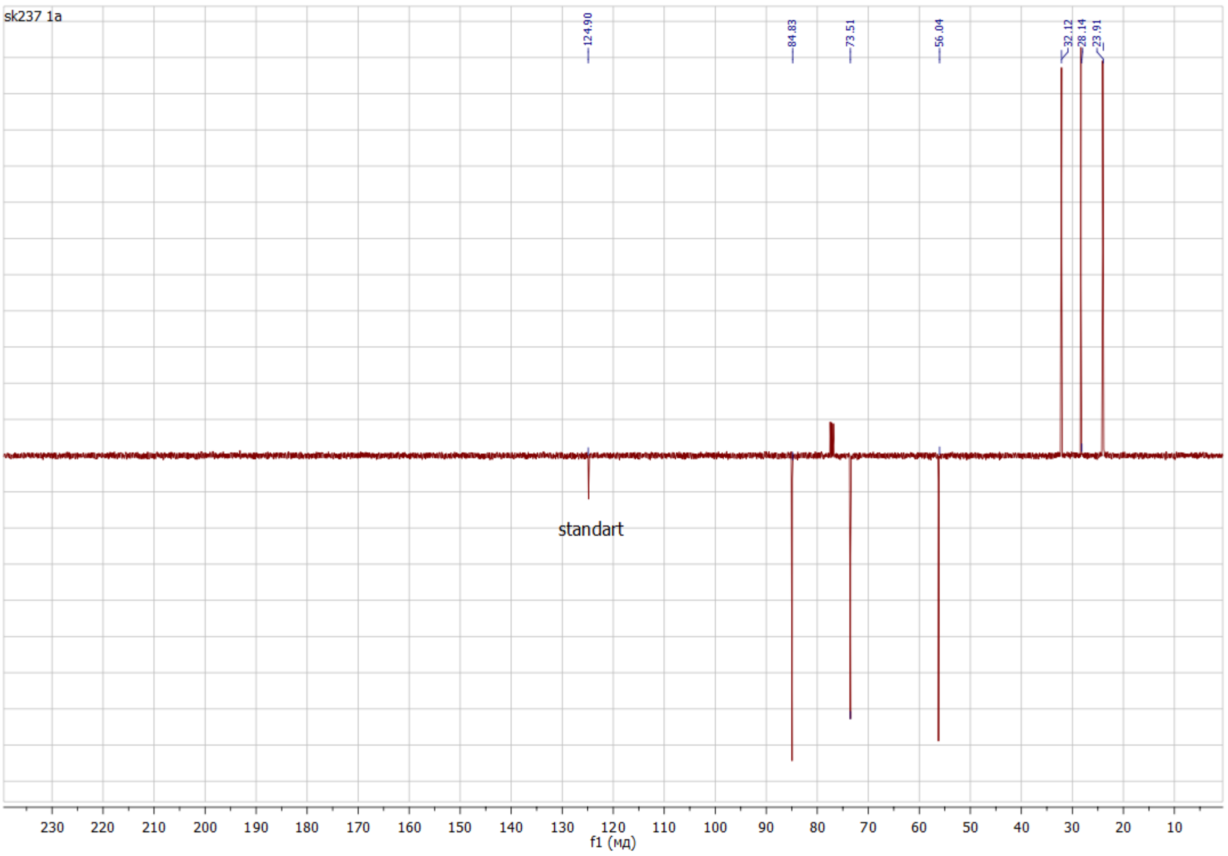

Figure S24. ^{13}C NMR (101 MHz) of 2-methoxycyclohexanol-1 in $CDCl_3$.

7.2 Opening of *trans*-cyclohexeneoxide (5) with EtOH. The same synthetic protocol as in section 7.1 was used. The only exception was that EtOH (10 mL) was employed instead of MeOH. The ^{1}H and ^{13}C spectra (Figures S25 and S26) corresponded to the literature data.[S15] According to the data the chemical yield of the final product was 20%. ^{1}H NMR (400 MHz, DMSO-d_6, 25 °C): δ = 3.78-3.66 (m, 1H), 3.51-3.33 (m, 2H), 3.08-2.96 (m, 1H), 2.84 (br.s, 1H), 2.16-1.90 (m, 2H), 1.81-1.63 (m, 2H), 1.32-1.05 (m, 7H) ppm. ^{13}C NMR (101 MHz, DMSO-d_6, 25 °C): δ = 83.46, 73.71, 64.00, 31.99, 29.21, 24.26, 23.97, 15.64 ppm.

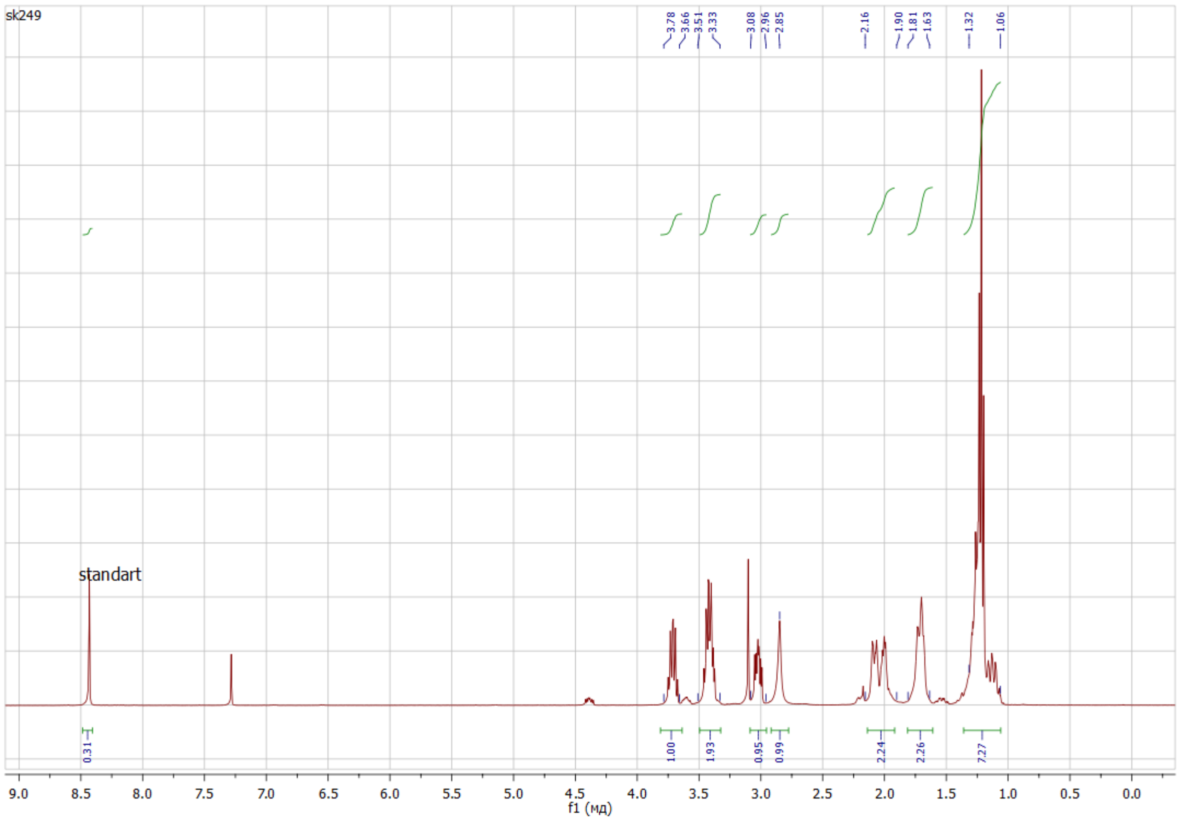

Figure S25. ^{1}H NMR (400 MHz) spectra of 2-ethoxycyclohexanol-1 in $CDCl_3$.

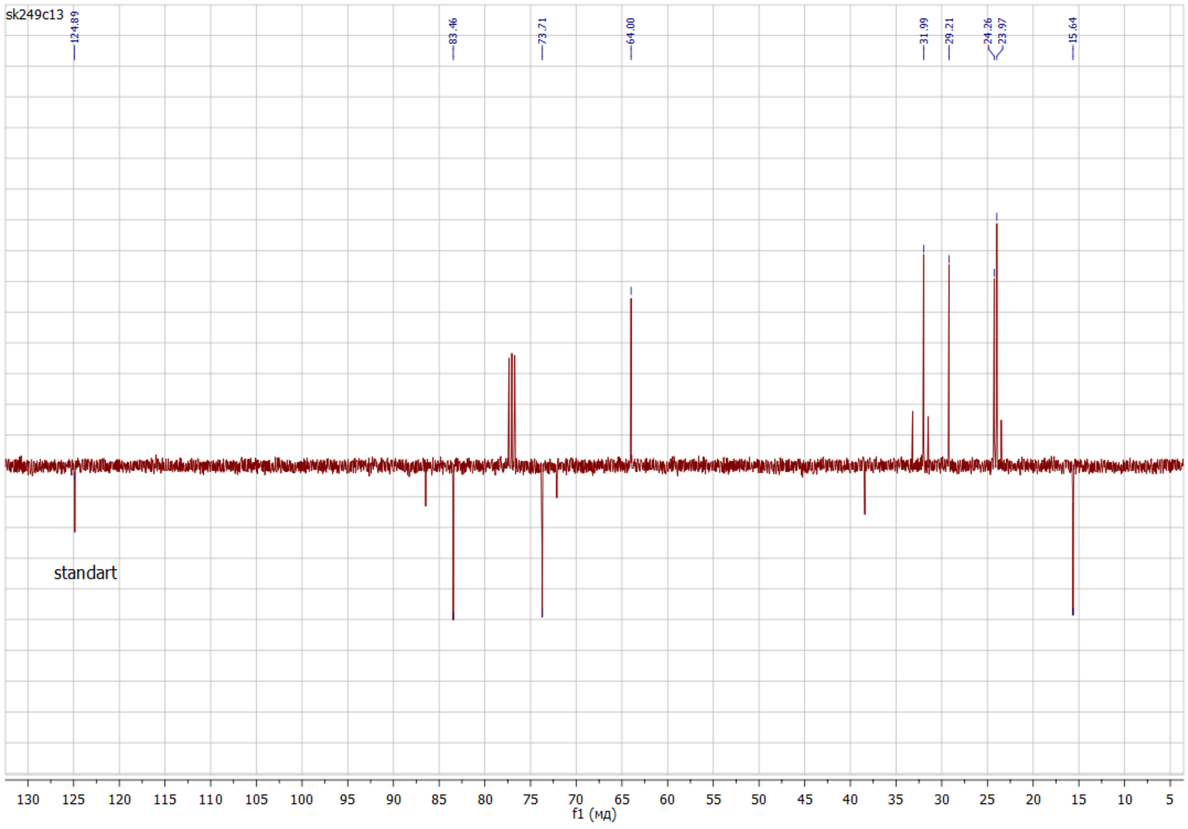

Figure S26. ^{13}C NMR (101 MHz) of 2-ethoxycyclohexanol-1 in $CDCl_3$.

8. Opening of volatile epoxides with D_2O in the three phase D_2O/CH_2Cl_2/F-1 system

In order to assess the real ratios of the products and the amounts of the remaining initial reagents the evaporation of the layers and the filtration of **F-1** had to be avoided. The amounts of the products and the epoxides were estimated directly in the reaction solutions by ^{1}H NMR with different standrrds to each layer added. The typical experiment is described in 8.1. section.

8.1. Opening of 1,2-propoxide with D_2O. The catalyst **F-1** (0.011 g, 1.15×10^{-5} mol) was added to a solution of 1,2-propoxide (0.06 mL, 0.91×10^{-3} mol) in 1.5 mL of CH_2Cl_2 with stirring, then 3 mL of D_2O was added, and the mixture stirred (700-1400 rpm) for 24 h at rt. Then the reaction mixture was left for a period of time to let the layers part. Then the layers were carefully separated and to the upper D_2O layer was added *t*-BuOH (0.011 g, 1.48×10^{-4} mol). To the bottom CH_2Cl_2 layer was added (0.0047 g, 2.8×10^{-5} mol) of *p*-dinitrobenzene and a small portion was taken from the solution and diluted with $CDCl_3$. It was the sample used to estimate by ^{1}H NMR the amount of the product in the layer (Figure S27). The same amount of *p*-dinitrobenzene was added to the middle layer followed by 1 mL of DMSO-d_6. Each solution was analyzed by ^{1}H NMR. The upper layer contained 67% of the diol, the middle fraction had 15% of the diol (Figure S28) and the bottom layer contained only 13% of the initial epoxide (Figure S29). Total yield of 1,2-propanediol was 82%.

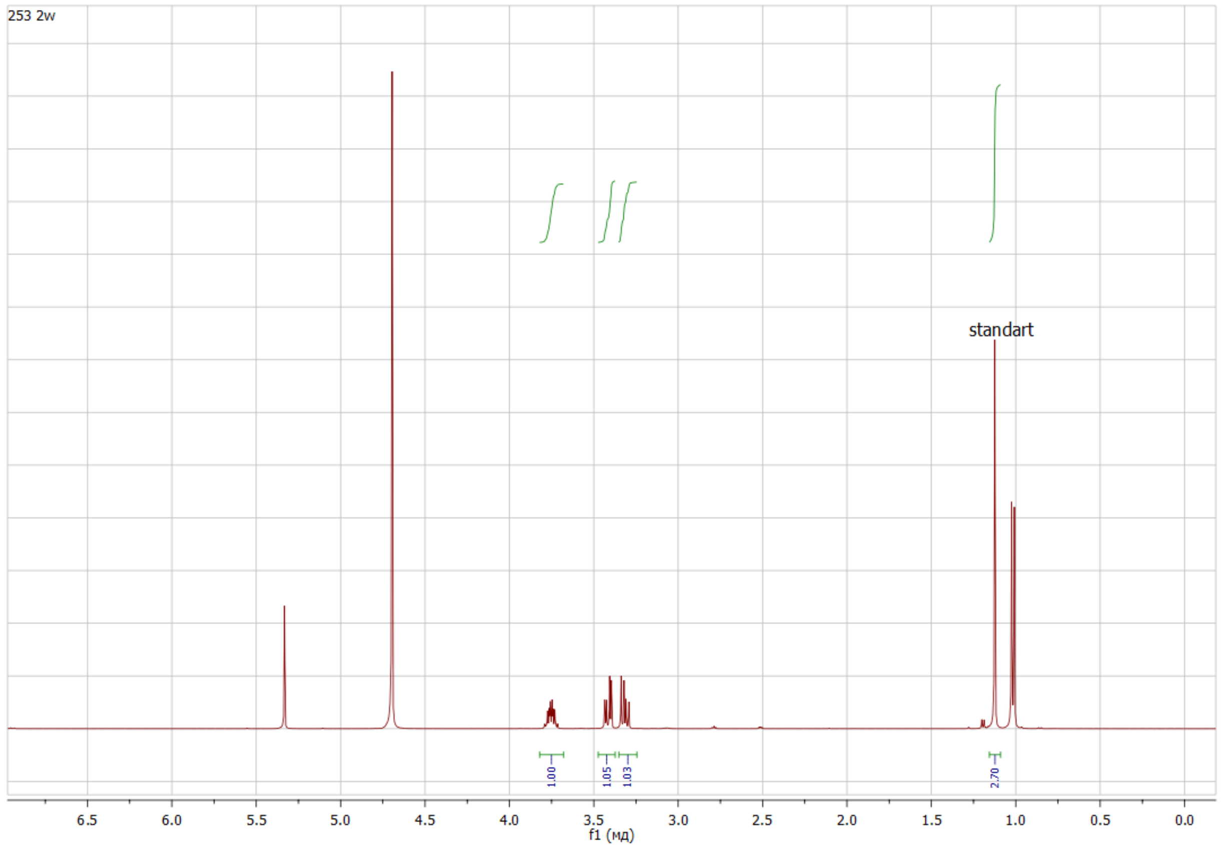

Figure S27. ^{1}H NMR spectra of 1,2-propanediol in D_2O (upper layer).

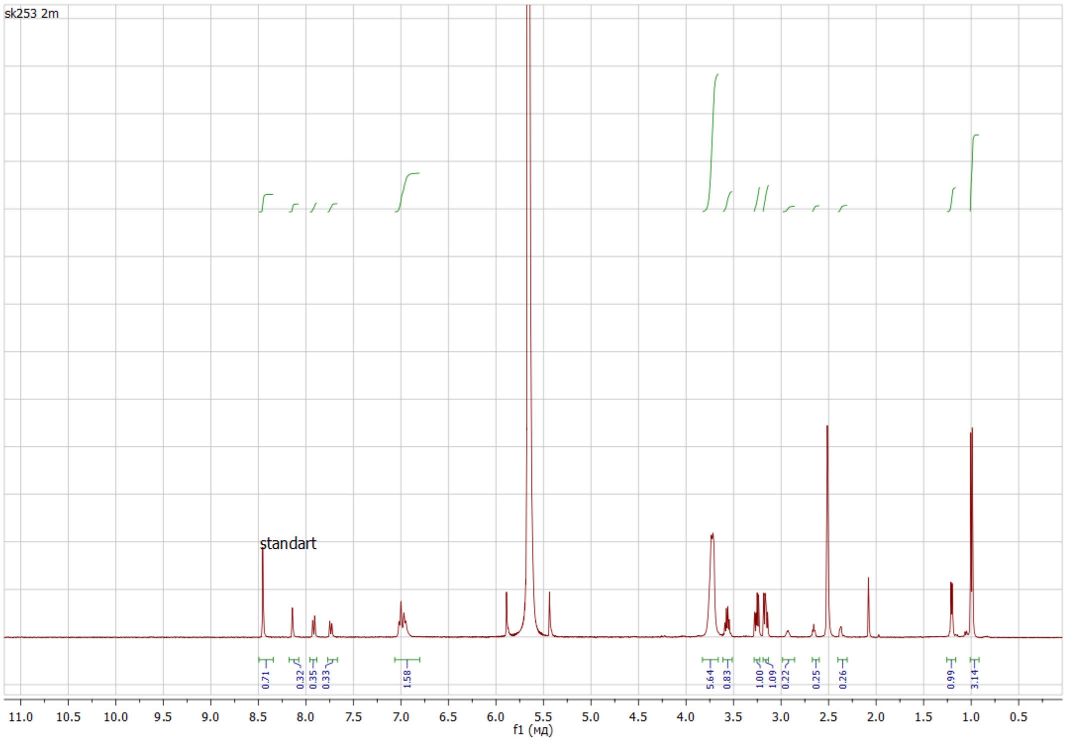

Figure S28. ^{1}H NMR spectra of 1,2-propanediol in DMSO-d_6 (middle layer). The region 6.9–8.3 ppm is related to the resonances of **F-1**. The resonances of the initial epoxides are also observed.

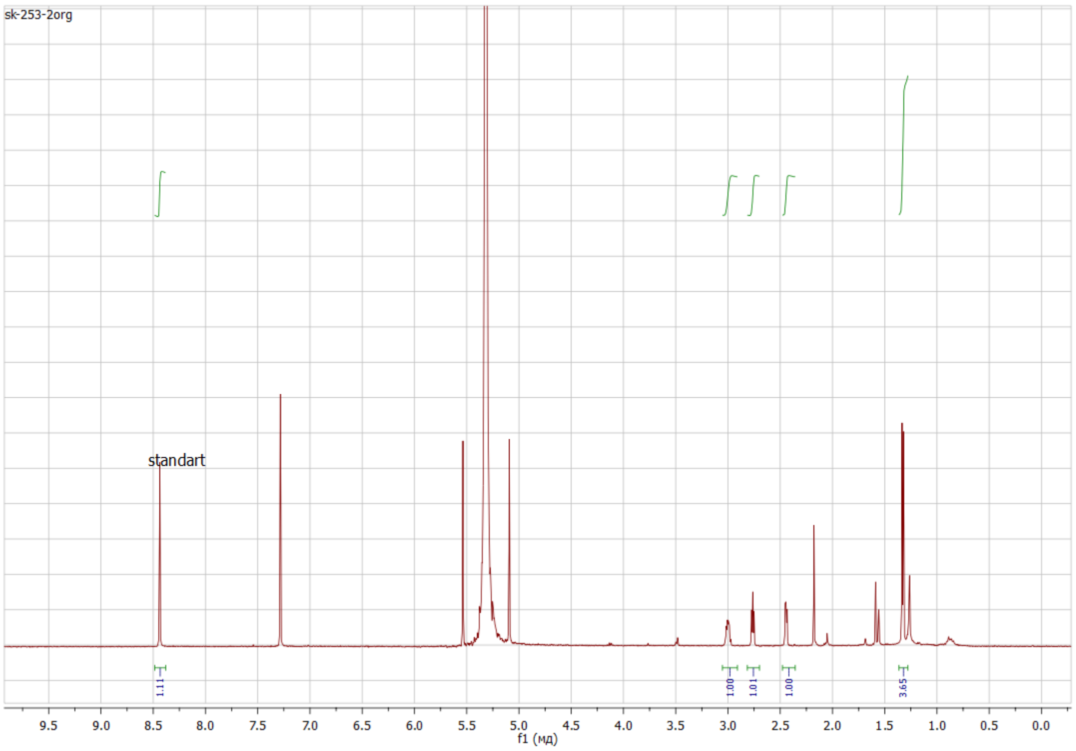

Figure S29. ^{1}H NMR spectra (in $CDCl_3$) of a sample of propene oxide opening (bottom CH_2Cl_2 layer).

8.2. Opening of 1,2-butene oxide with D_2O. The experiment was run as described in section 8.1. ^{1}H NMR spectra of the layers are presented in Figures S30–S32. The product yields were 60% in the upper layer, 10% in the middle one and almost no product was found in the bottom layer.

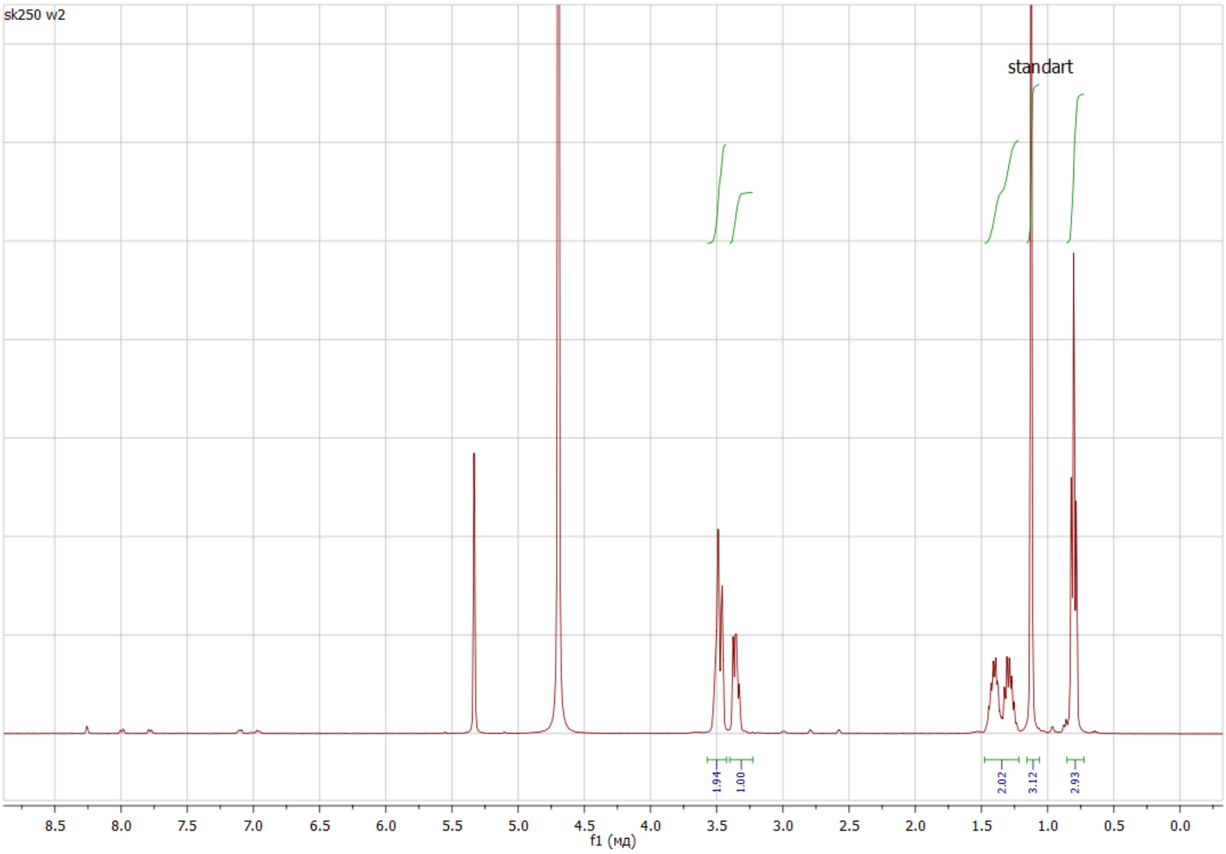

Figure S30. ^{1}H NMR spectra of 1,2-butanediol in D_2O (upper layer).

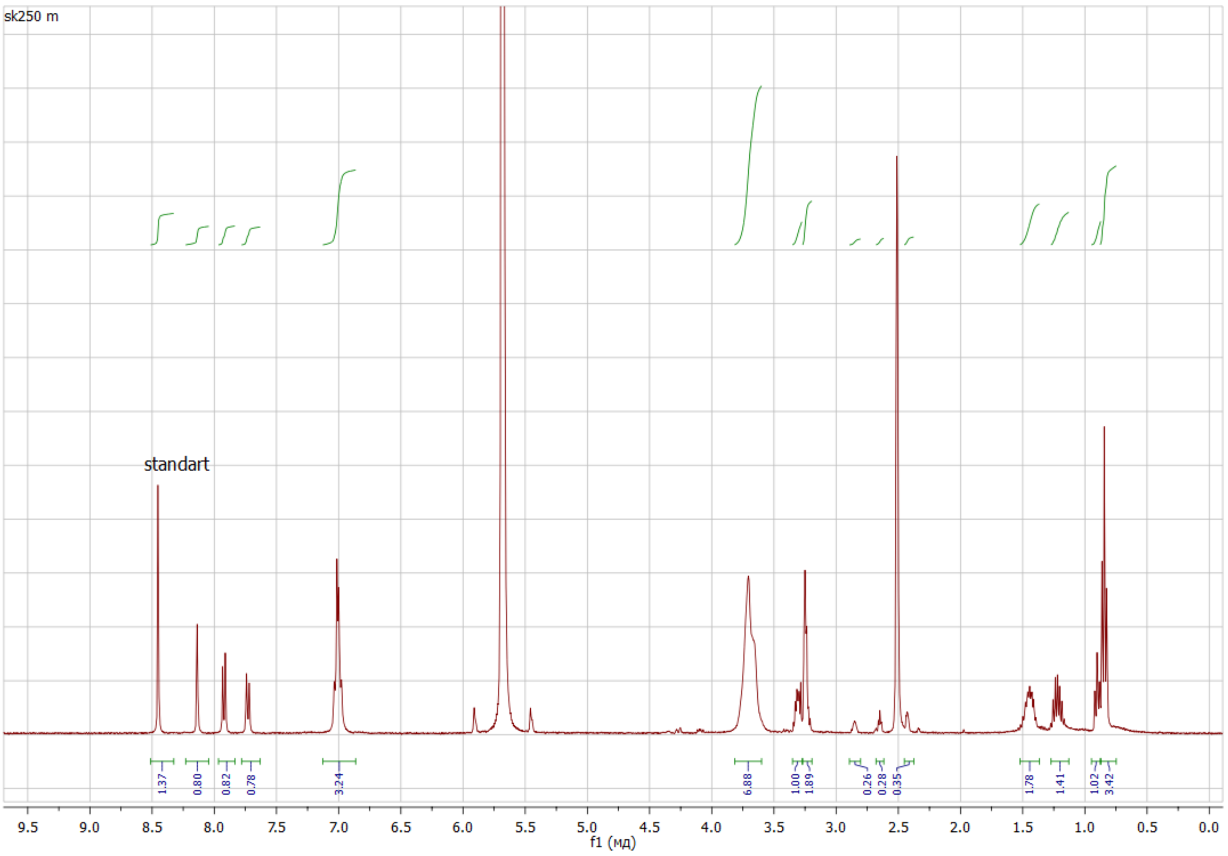

Figure S31. ^{1}H NMR (400 MHz) spectra of 1,2-butanediol in DMSO-d_6 (middle layer).

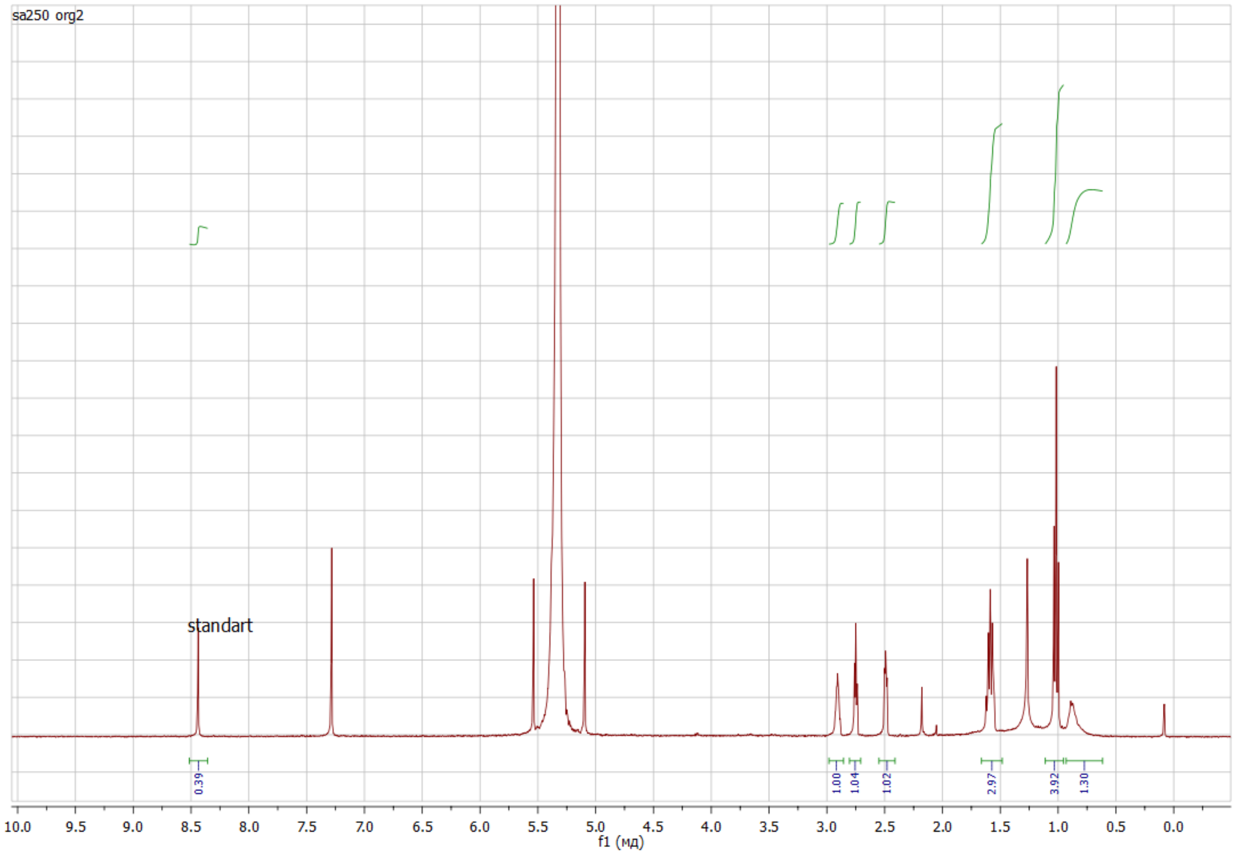

Figure S32. ^{1}H NMR spectra of a sample of the bottom CH_2Cl_2 layer in $CDCl_3$.

8.3. Opening of 1,2-hexene oxide with D_2O. The experiment was run as described in the section 8.1. ^{1}H NMR spectra of the layers are presented in Figures S33–S35. The product yield was only 2%.

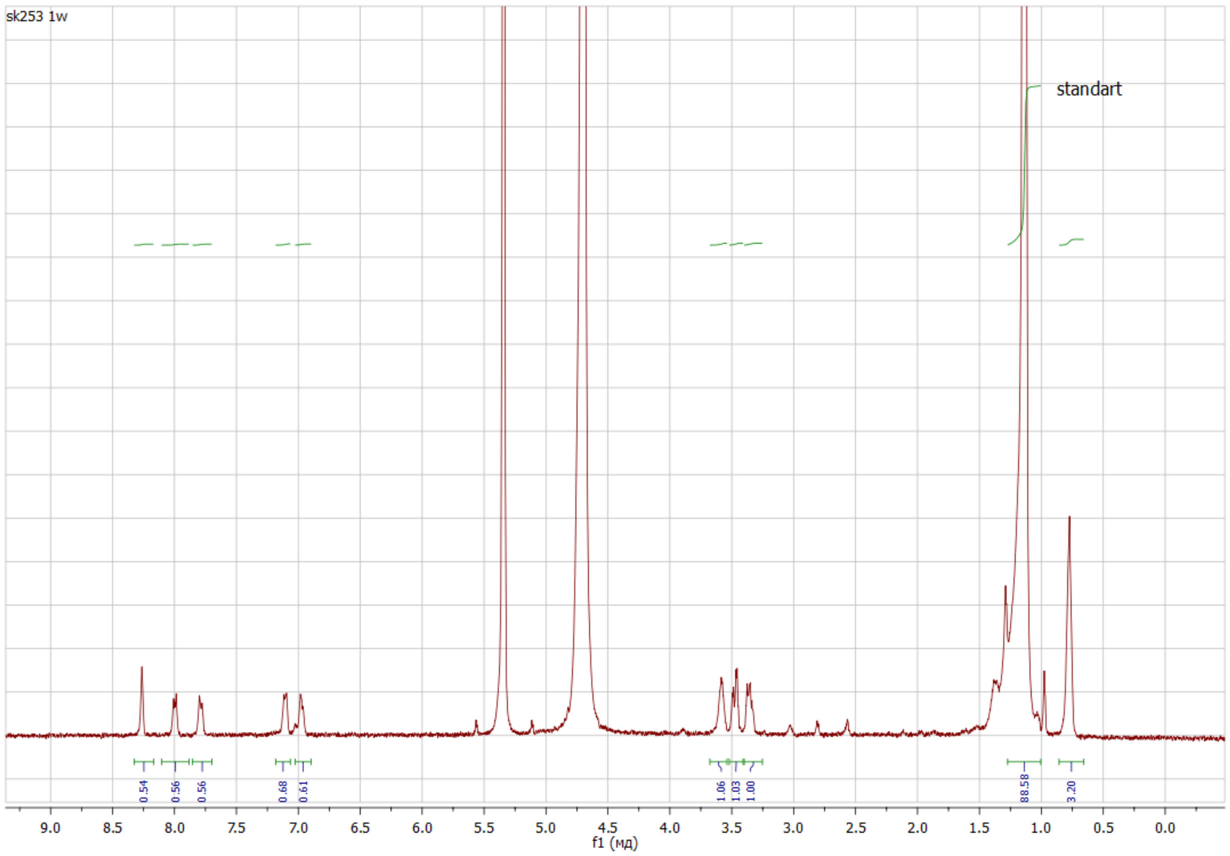

Figure S33. ^{1}H NMR spectra of 1,2-hexanediol in D_2O (upper layer). The region 6.9–8.3 ppm is related to the resonances of **F-1**. The resonances of the initial epoxides are also observed.

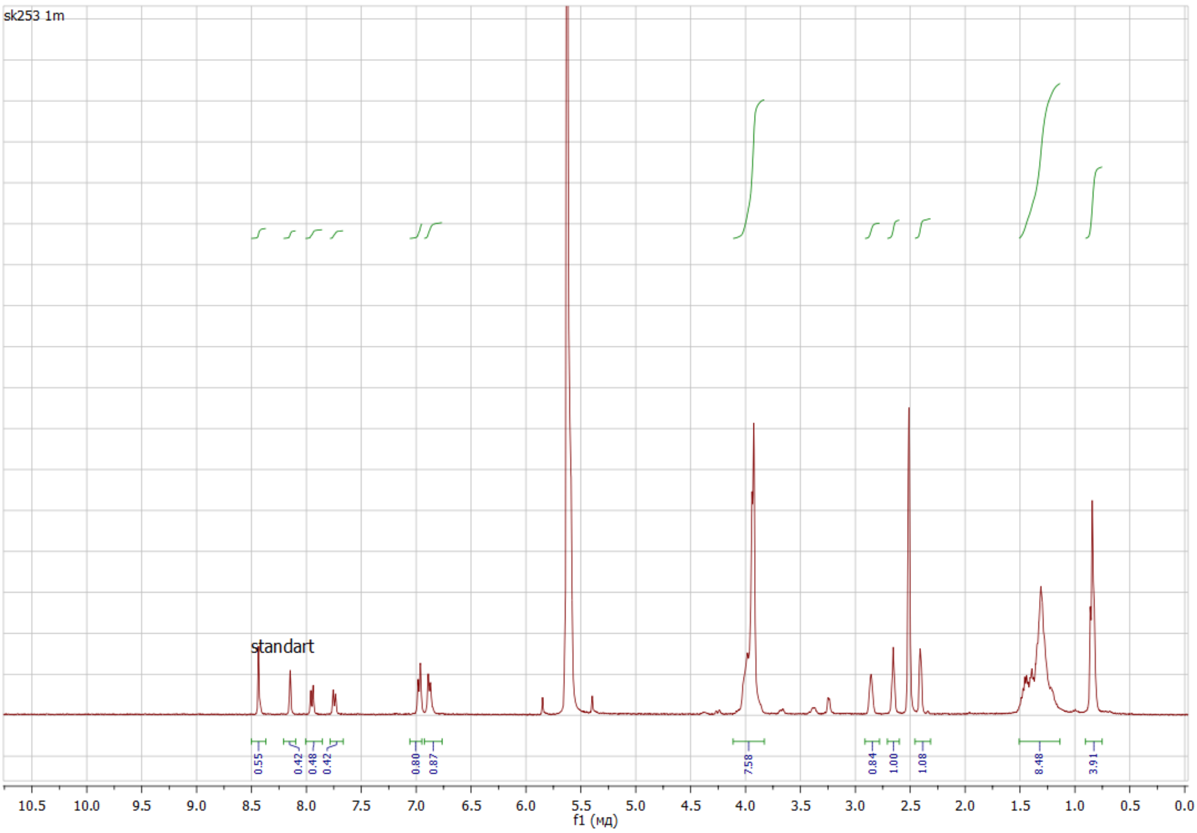

Figure S34. ^{1}H NMR (in DMSO-d_6) spectra of the middle layer of 1,2-hexene oxide opening.

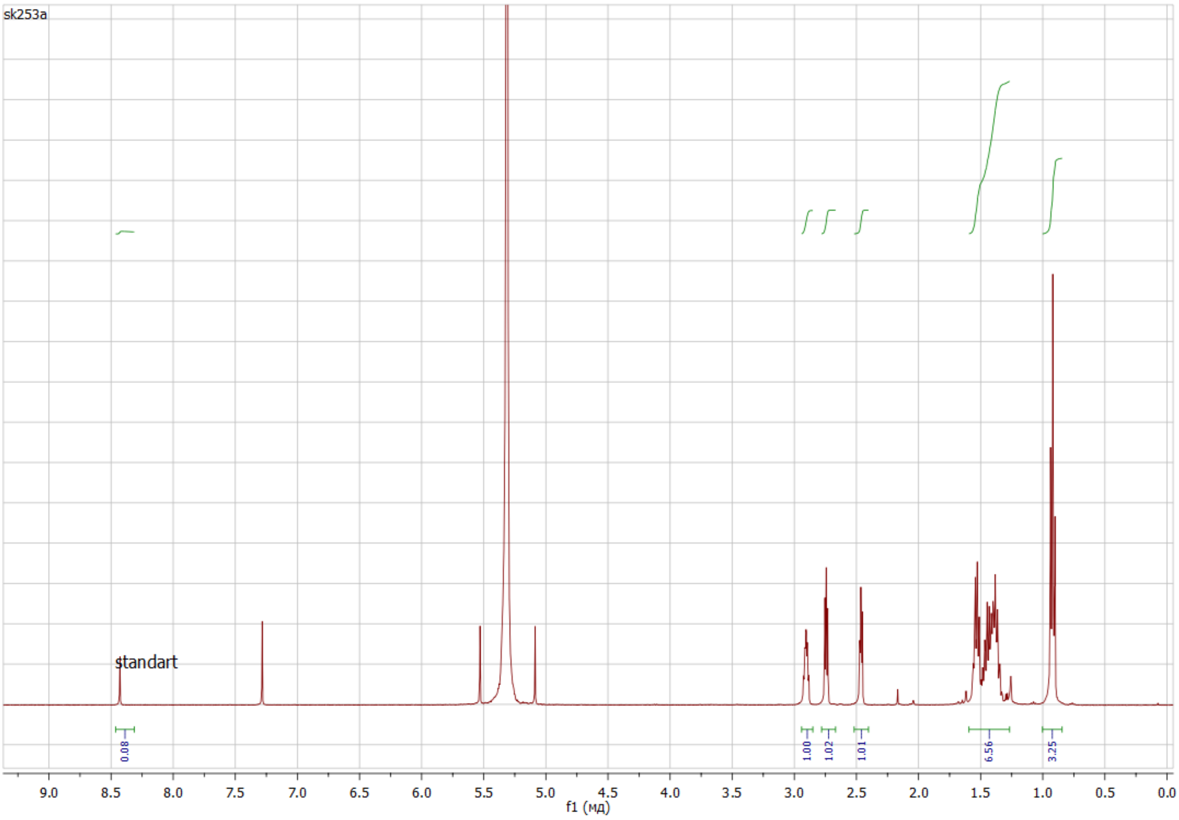

Figure S35. ^{1}H NMR spectra (in $CDCl_3$) of a sample of 1,2-hexene oxide opening (bottom CH_2Cl_2 layer).

9. Opening of propene oxide with H_2O. The catalyst **F-1** (0.023 g) was added to a solution of 1,2-propene oxide (0.12 mL, 1.83×10^{-3} mol) in 5 mL of CH_2Cl_2 with stirring, then 10 mL of twice distilled H_2O was added, and the mixture stirred (700–1400 rpm) for 24 h at rt. Then the mixture was filtered and the layers were separated. The insoluble **F-1**was removed by filtration and the filtrate evaporated. To the residue were added p-dinitrobenzene (as a standard, 0.009 g, 5.3×10^{-5} mol) and $CDCl_3$ (1 mL). The remaining insoluble in $CDCl_3$ **F-1**was filtered and the ^1H and ^{13}C NMR spectra of the residue were run (Figures S36 and S37). The spectra corresponded to the literature data.[S16] According to the data the chemical yield of the final product was 56%. ^1H NMR (400 MHz, $CDCl_3$) δ 8.43 (s, 1H), 4.08–3.76 (m, 3H), 3.58 (dd, J = 11.3, 2.6 Hz, 1H), 3.37 (dd, J = 11.3, 8.0 Hz, 1H), 1.13 (d, J = 6.4 Hz, 3H). ^{13}C NMR (101 MHz, DMSO-d_6, 25 °C): δ = 68.32, 67.88, 18.67 ppm.

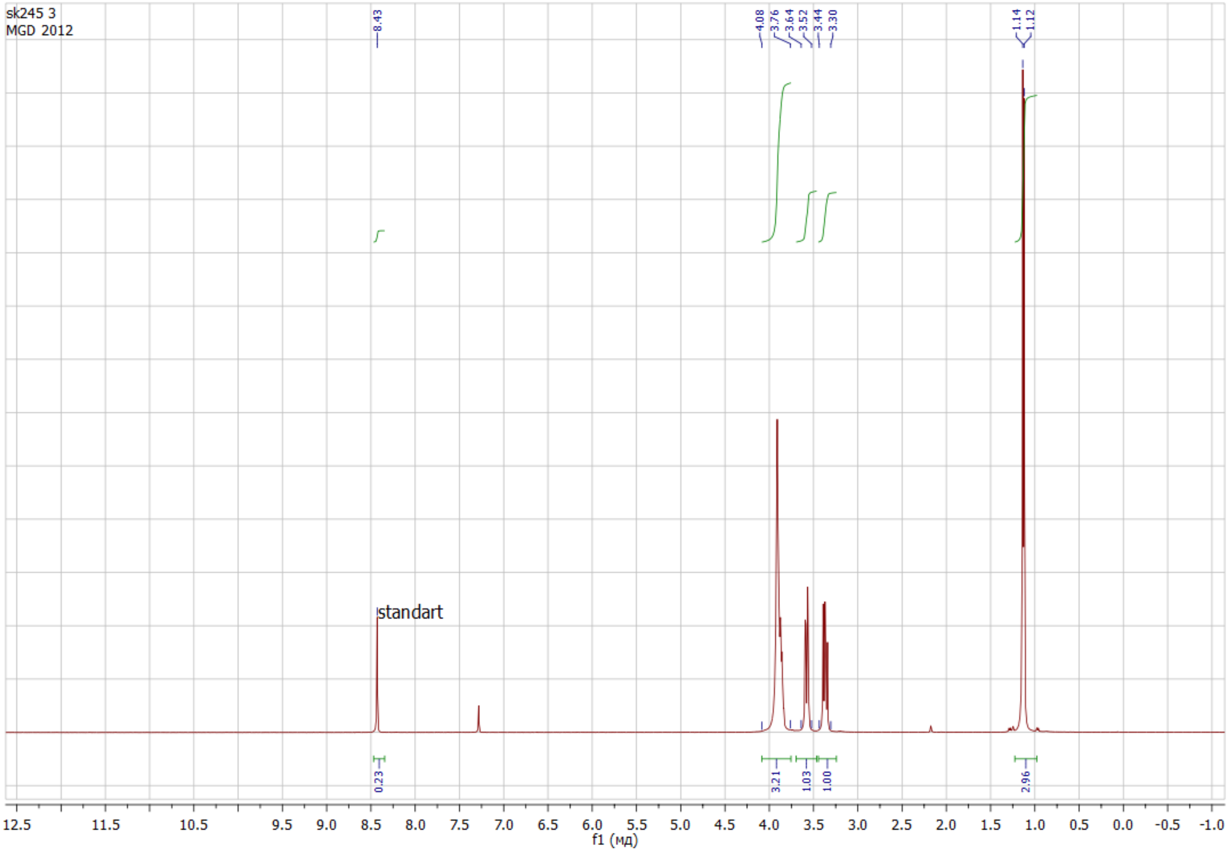

Figure S36. ^1H NMR (400 MHz) spectra of 1,2-propanediol in $CDCl_3$.

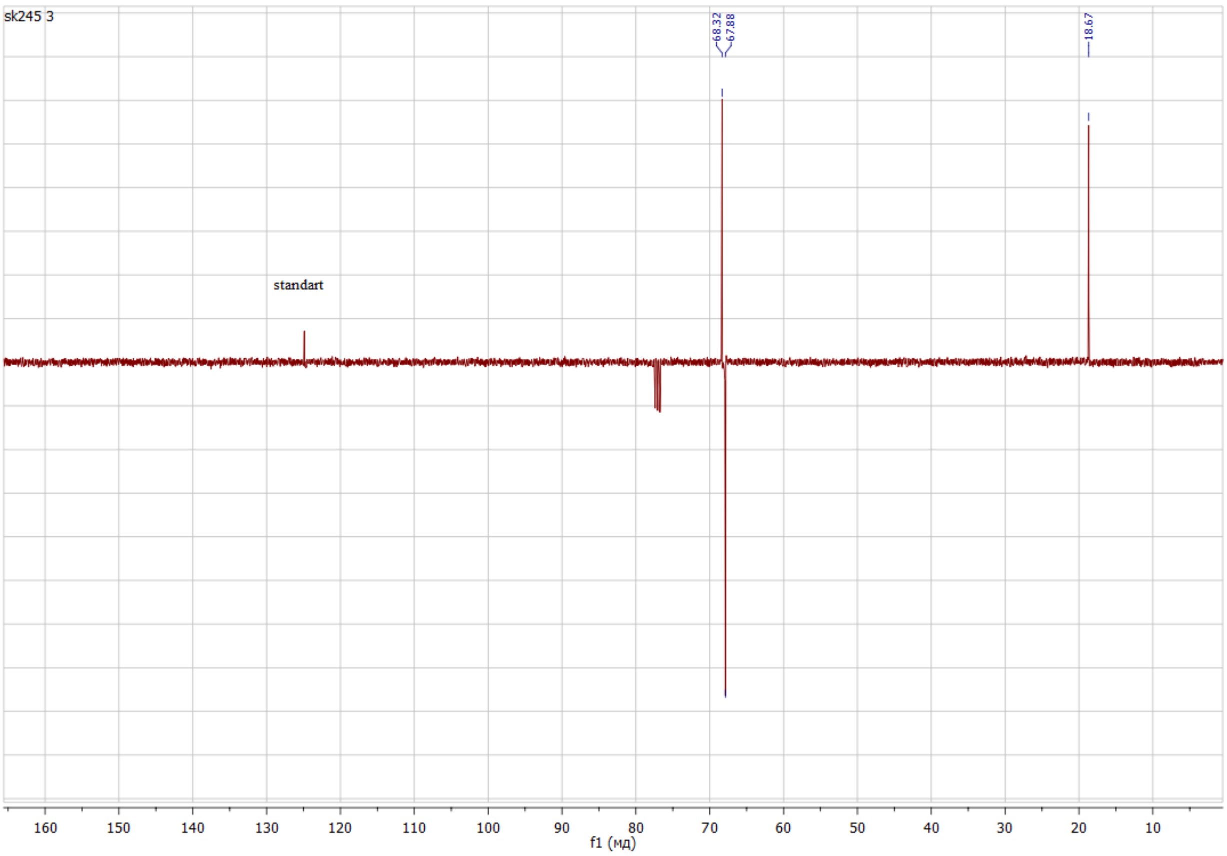

Figure S37. ^{13}C NMR (101 MHz) of 1,2-propanediol in $CDCl_3$.

10. Opening of 1,2-butene oxide with H_2O. The same protocol was used as in section 8 with the exception that 1,2-butene oxide (0.009 g, 5.3×10^{-5} mol) was used. According to the data the 1,2-butane diol was obtained in a yield of 11%. ^{1}H NMR (400 MHz, DMSO-d_6, 25 °C): δ = 3.71-3.59 (m, 2H), 3.51-3.40 (m, 1H), 2.75 (br.s, 2H), 1.54-1.42 (m, 2H), 0.98 (t, J=7.48, 3H) ppm. ^{13}C NMR (101 MHz, DMSO-d_6, 25 °C): δ = 73.72, 66.43, 26.07, 9.92 ppm. The spectra corresponded to the literature data.[S17]

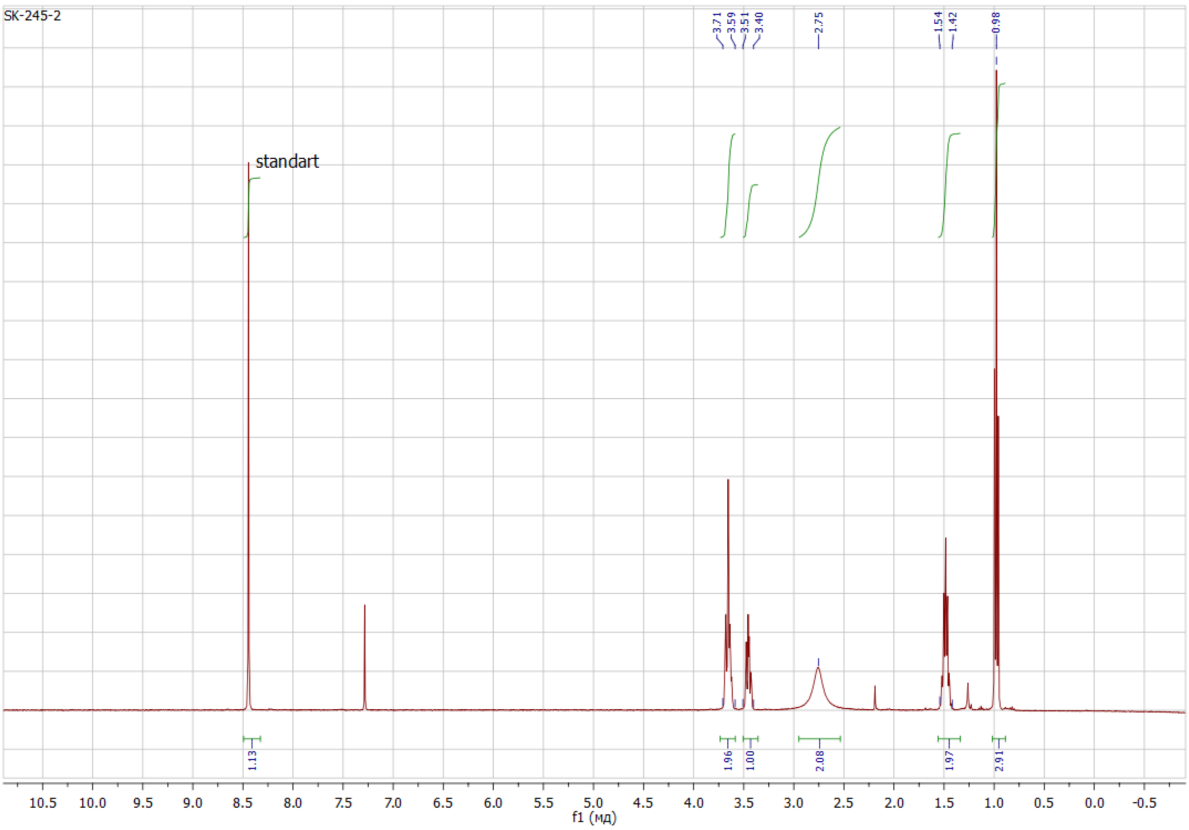

Figure S38. ^{1}H NMR (400 MHz) spectra of 1,2-butanediol in $CDCl_3$.

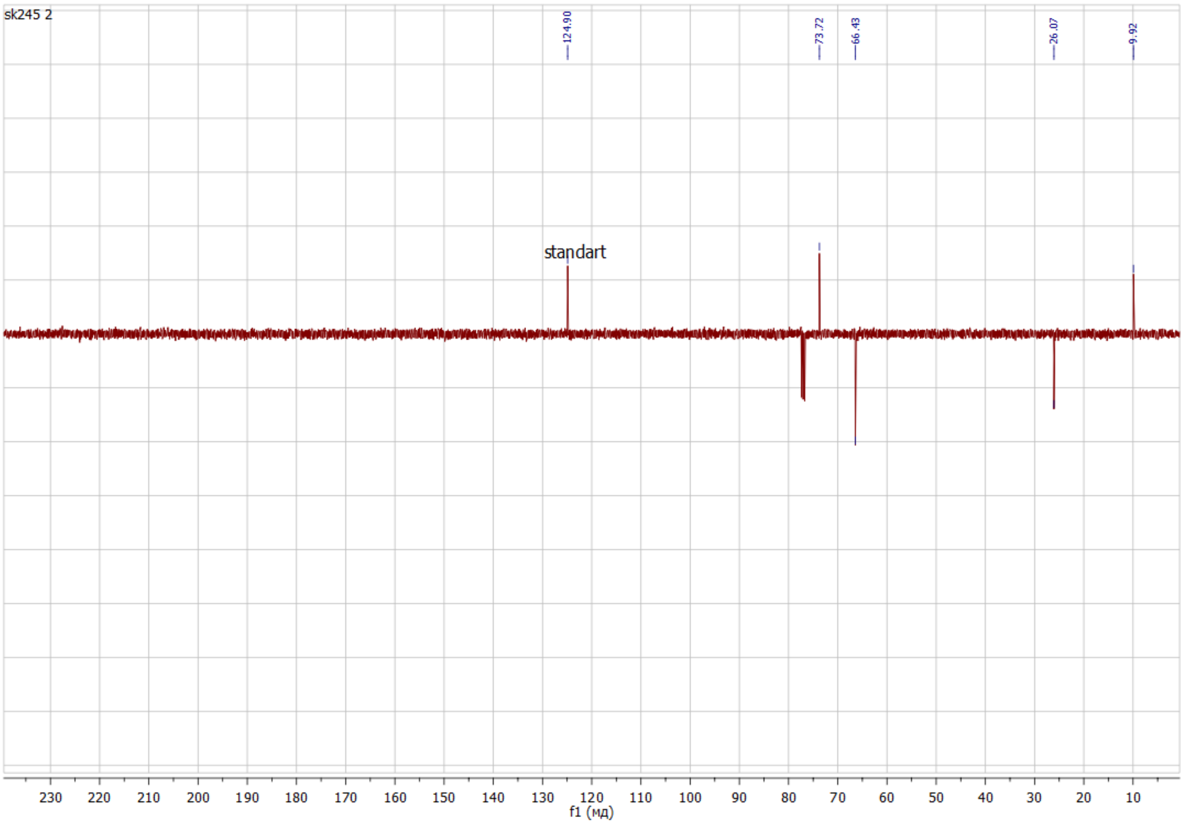

Figure S39. ^{13}C NMR (101 MHz) of 1,2-butanediol in $CDCl_3$.

11. Opening of 1,2-hexene oxide with H_2O. The same protocol was used as that of sections 8 and 9 with the exception of 1,2-hexene oxide (0.22 mL, 1.83×10^{-3} mol) being used instead of the previous epoxides and the reaction was run for 6 days at rt. According to its ^1H NMR spectra the yield of 1,2-hexanediol was 10%. ^1H NMR (400 MHz, DMSO-d_6, 25 °C): δ = 3.76-3.70 (m, 1H), 3.66 (dd, J=2.90, 11.10, 1H), 3.44 (dd, J=7.74, 11.10, 1H), 2.49 (s, 2H), 1.51-1.26 (m, 6H), 0.92 (t, J=6.95, 3H) ppm. ^{13}C NMR (101 MHz, DMSO-d_6, 25 °C): δ = 72.35, 66.82, 32.84, 27.71, 22.71, 14.00 ppm. The spectra corresponded to the literature data.[S18]

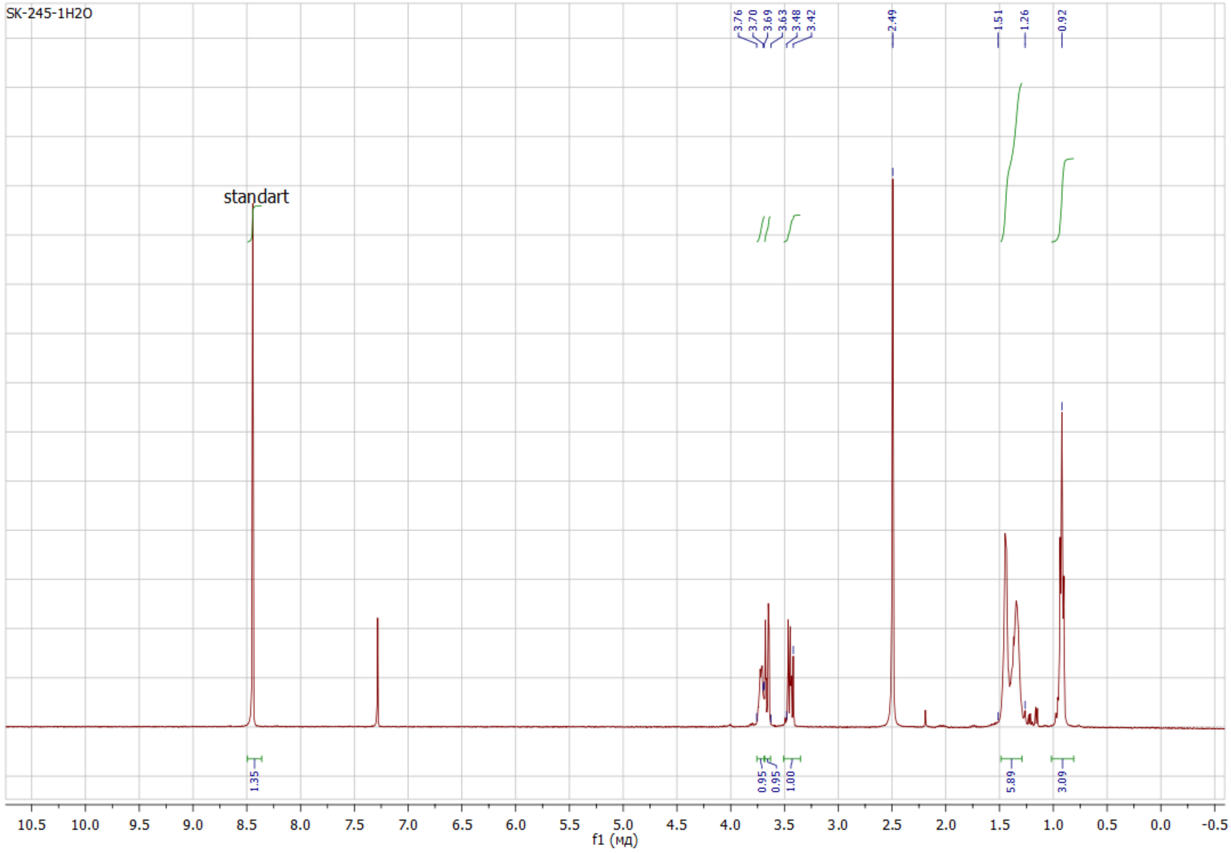

Figure S40. ^1H NMR (400 MHz) spectra of 1,2-hexanediol in $CDCl_3$.

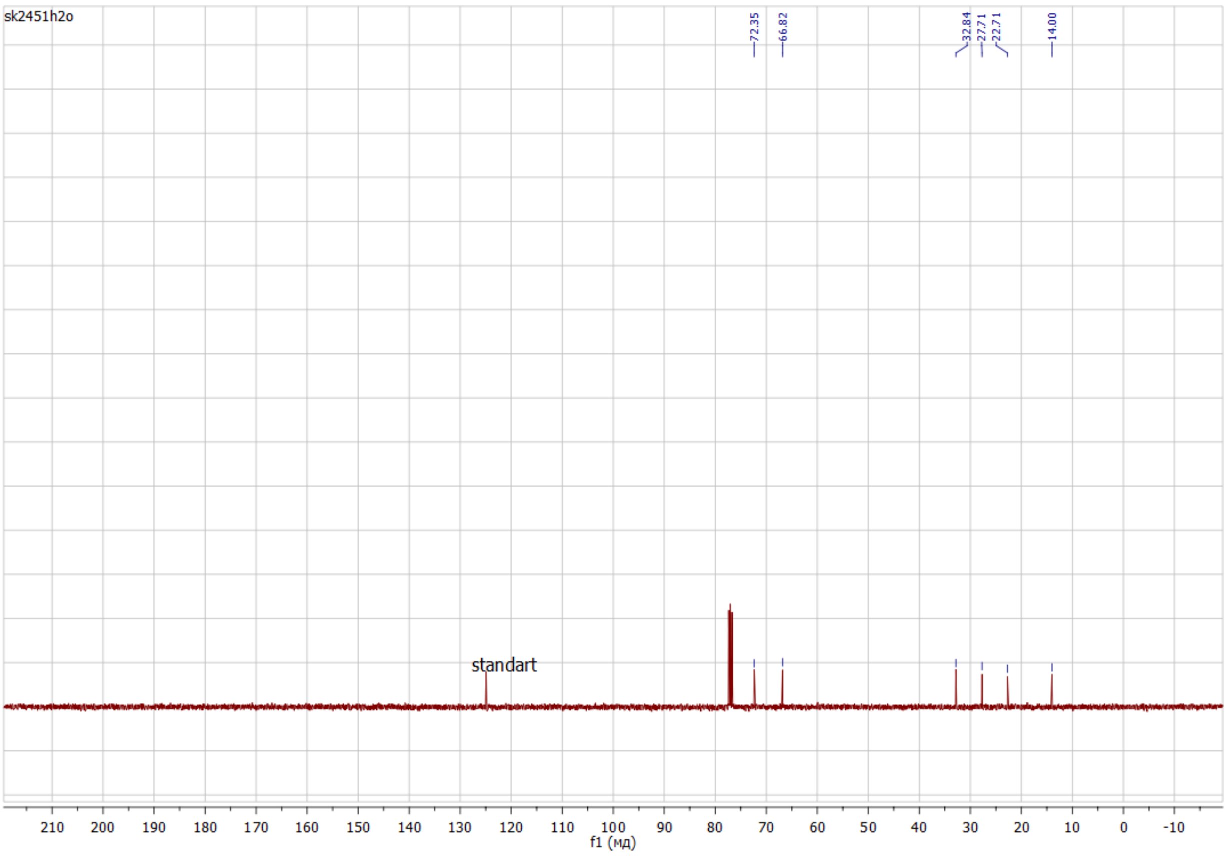

Figure S41. ^{13}C NMR (101 MHz) of 1,2-hexanediol in $CDCl_3$.

12. Catalysis of a Diels–Alder reaction. Cyclopentadiene (0.9 mL, 10.1 × 10^{-3} mol) was added to a solution of butenone (0.6 mL, 0.51 g, 7.4 × 10^{-3} mol) in dry heptane (40 mL) at stirring, and then **F-1** (0.094g, 5.3% mol, 9.8 × 10^{-5} mol) was added. The mixture was stirred for 3 h at rt. Then the catalyst was filtered and the filtrate evaporated to remove the initial unreacted reagents and the solvent at a reduced pressure to give the mixture of exo- and endo-products (Scheme 3) in a yield of 52% (0.52 g, 3.8 × 10^{-3} mol) as a mixture of endo/exo-isomers in a ratio 78/22. ^{1}H NMR (400 MHz, $CDCl_3$, 25 °C) corresponded to the literature data.[S19] m/z 159.0798, calculated for $C_9H_{12}O$, $[M+Na]^+$ 159.09.

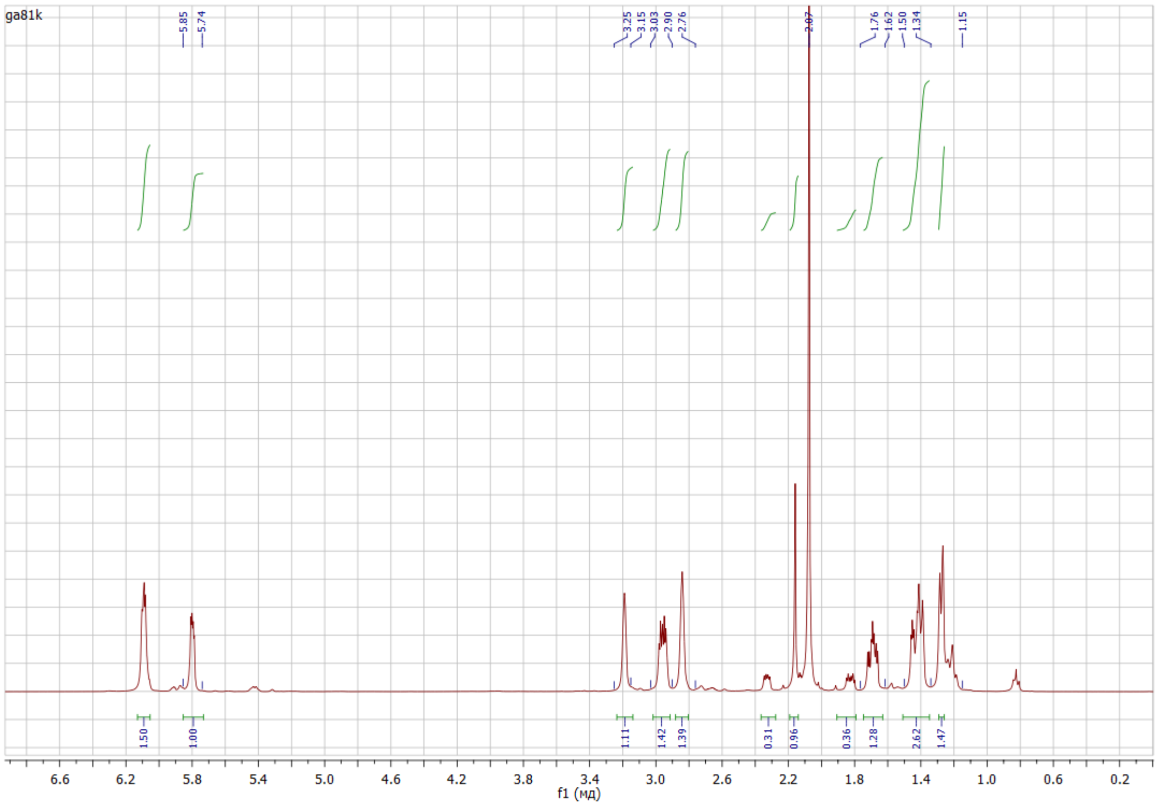

Figure S42. ^{1}H NMR (400 MHz) spectra of a mixture of endo and exo isomers of the Diels–Alder reaction.

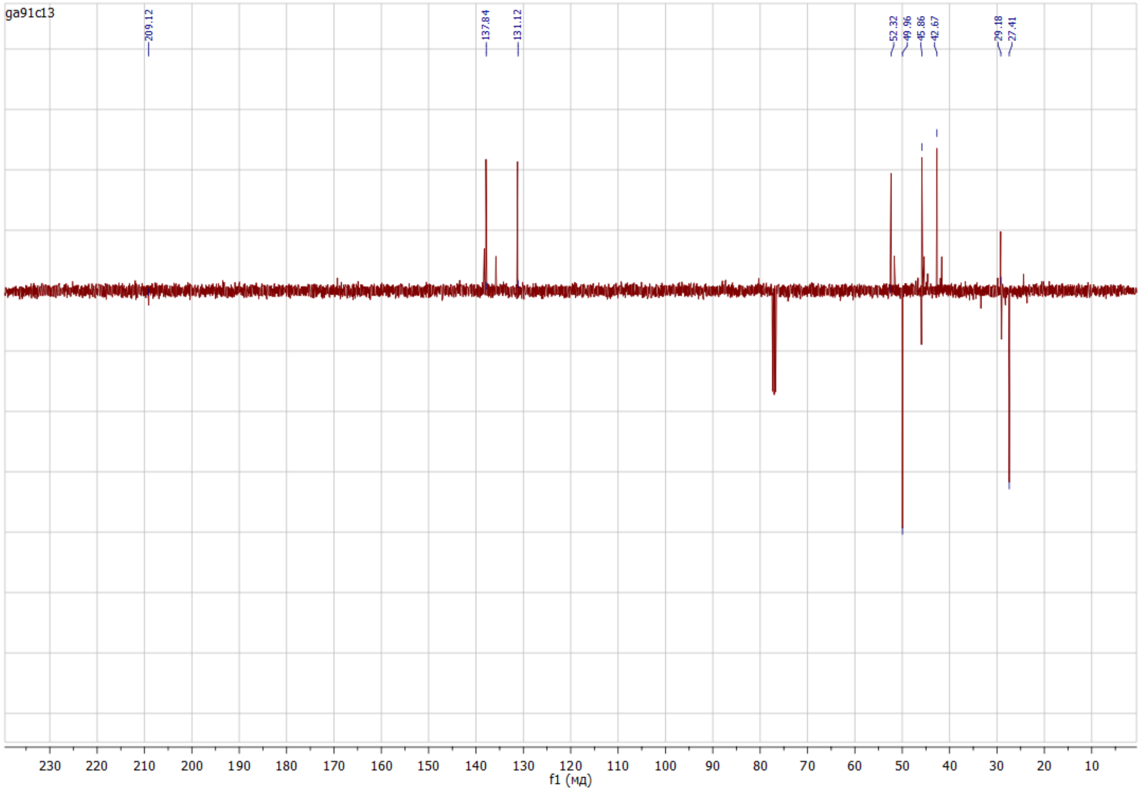

Figure S43. ^{13}C NMR (101 MHz, $CDCl_3$, 25 °C) of a mixture of endo and exo isomers of the Diels–Alder reaction.

References

S1. Dolomanov, O.V., Bourhis, L.J., Gildea, R.J, Howard, J.A.K. & Puschmann, H. *J. Appl. Cryst*. **2009**, *42*, 339-341, the structures were solved with the ShelXT [Sheldrick, G.M. *Acta Cryst*. **2015**, A71, 3-8.

S2. Bourhis, L.J.; Dolomanov, O.V.; Gildea, R.J.; Howard, J.A.K.; Puschmann, H. *Acta Cryst*. **2015**, A71, 59-75.

S3. Frisch, M. J.; Trucks, G. W.; Schlegel, H. B.; Scuseria, G. E.; Robb, M. A.; Cheeseman, J. R.; Scalmani, G.; Barone, V.; Petersson, G. A.; Nakatsuji, H.; et al. *Gaussian16 Revision A.03*; 2016.

S4. Zhao, Y.; Truhlar, D. G. The M06 Suite of Density Functionals for Main Group Thermochemistry, Thermochemical Kinetics, Noncovalent Interactions, Excited States, and Transition Elements: Two New Functionals and Systematic Testing of Four M06-Class Functionals and 12 Other Functionals. *Theor. Chem. Acc.* **2008**, *120* (1–3), 215–241. https://doi.org/10.1007/s00214-007-0310-x.

S5. Weigend, F.; Ahlrichs, R. Balanced Basis Sets of Split Valence, Triple Zeta Valence and Quadruple Zeta Valence Quality for H to Rn: Design and Assessment of Accuracy. *Phys. Chem. Chem. Phys.* **2005**, *7* (18), 3297. https://doi.org/10.1039/b508541a.

S6. Austin, A.; Petersson, G. A.; Frisch, M. J.; Dobek, F. J.; Scalmani, G.; Throssell, K. A Density Functional with Spherical Atom Dispersion Terms. *J. Chem. Theory Comput.* **2012**, *8* (12), 4989–5007. https://doi.org/10.1021/ct300778e.

S7. Lian, P.; Johnston, R. C.; Parks, J. M.; Smith, J. C. Quantum Chemical Calculation of pK_as of Environmentally Relevant Functional Groups: Carboxylic Acids, Amines, and Thiols in Aqueous Solution. *J. Phys. Chem. A* **2018**, *122* (17), 4366–4374. https://doi.org/10.1021/acs.jpca.8b01751.

S8. Marenich, A. V.; Cramer, C. J.; Truhlar, D. G. Universal Solvation Model Based on Solute Electron Density and on a Continuum Model of the Solvent Defined by the Bulk Dielectric Constant and Atomic Surface Tensions. *J. Phys. Chem. B* **2009**, *113* (18), 6378–6396. https://doi.org/10.1021/jp810292n.

S9. Avdeef, A. *Absorption and Drug Development: Solubility, Permeability, and Charge State*; John Wiley & Sons, 2012.

S10. Shah, S. T. A. ; Singh, S.; Guiry, P. J. A Novel, Chemoselective and Efficient Microwave-Assisted Deprotection of Silyl Ethers with Selectfluor, J. *Org. Chem*. **2009**, *74,* 2179–2182.

S11. Łowicki, D.; Bezłada, A.; Mlynarski, J. Asymmetric Hydrosilylation of Ketones Catalyzed by Zinc Acetate with Hindered Pybox Ligands. *Adv. Synth. Catal.* **2014**, *356*, 591–595.

S12. Plietker, B.; Niggemann, M.; Pollrich, A. The Acid Accelerated Ruthenium-Catalysed Dihydroxylation. Scope and Limitations. *Org. Biomol. Chem*., **2004**, *2*, 1116–1124.

S13. Caron, G.; Kazlauskas, R.J. An Optimized Sequential Kinetic Resolution of trans-1,2-Cyclohexanediol. *J. Org. Chem*. **1991**, *56*, 7251–7256.

S14. Córdova, A.; Sunden, H.; Anders Bøgevig, A.; Johansson, M.; Fahmi Himo, F. The Direct Catalytic Asymmetric a-Aminooxylation Reaction: Development of Stereoselective Routes to 1,2-Diols and 1,2-Amino Alcohols and Density Functional Calculations. *Chem. Eur. J.* **2004**, *10*, 3673–3684.

S15. Robinson, M. W. C.; Davies, A. M.; Buckle, R.; Mabbett, I.; Taylor, S. H.; Graham, A. E. Epoxide ring-opening and Meinwald rearrangement reactions of epoxides catalyzed by mesoporous aluminosilicates. *Org. Biomol. Chem.*, **2009,** *7*, 2559–2564.

S16. Troev, K.; Koseva, N.; Hägele, G. Novel Routes to Aminophosphonic Acids: Interaction of Dimethyl H-Phosphonate with Hydroxyalkyl Carbamates. *Heteroatom Chemistry*, **2008**, *19*, 119–124.

S17. Liu, X.; de Vries, J. G.; Werner, T. Transfer hydrogenation of cyclic carbonates and polycarbonate to methanol and diols by iron pincer catalysts. *Green Chemistry*, **2019**, *21*, 5248–5255.

S18 Lloyd-Jones, G. C.; Wall, Ph. D.; Slaughter, J. L.; Parkerb A. J.; Laffanc D. P. Enantioselective homoallyl-cyclopropanation of dibenzylideneacetone by modified allylindium halidereagents—rapid access to enantioenriched 1-styryl-norcarene. *Tetrahedron*, **2006,** *62*, 11402–11412.

S19. Rickerby, J.; Vallet, M.; Bernardinelli, G.; Viton, F.; Kündig, E. P. Ruthenium–Lewis Acid Catalyzed Asymmetric Diels–Alder Reactions between Dienes and α,β-Unsaturated Ketones. *Chem. Eur. J.*, **2007**, *13,* 3354–3368.
